# Minimally invasive biomarkers in human and non‐human primate evolutionary biology: Tools for understanding variation and adaptation

**DOI:** 10.1002/ajhb.23811

**Published:** 2022-10-07

**Authors:** Samuel S. Urlacher, Elizabeth Y. Kim, Tiffany Luan, Lauren J. Young, Brian Adjetey

**Affiliations:** ^1^ Department of Anthropology Baylor University Waco Texas USA; ^2^ Human Evolutionary Biology and Health Lab Baylor University Waco Texas USA; ^3^ Child and Brain Development Program CIFAR Toronto Ontario Canada; ^4^ Department of Biology Baylor University Waco Texas USA

## Abstract

**Background:**

The use of minimally invasive biomarkers (MIBs – physiological biomarkers obtained from minimally invasive sample types) has expanded rapidly in science and medicine over the past several decades. The MIB approach is a methodological strength in the field of human and non‐human primate evolutionary biology (HEB). Among humans and our closest relatives, MIBs provide unique opportunities to document phenotypic variation and to operationalize evolutionary hypotheses.

**Aims:**

This paper overviews the use of MIBs in HEB. Our objectives are to (1) highlight key research topics which successfully implement MIBs, (2) identify promising yet under‐investigated areas of MIB application, and (3) discuss current challenges in MIB research, with suggestions for advancing the field.

**Discussion and Conclusions:**

A range of MIBs are used to investigate focal topics in HEB, including energetics and life history variation/evolution, developmental plasticity, and social status and dominance relationships. Nonetheless, we identify gaps in existing MIB research on traits such as physical growth and gut function that are central to the field. Several challenges remain for HEB research using MIBs, including the need for additional biomarkers and methods of assessment, robust validations, and approaches that are standardized across labs and research groups. Importantly, researchers must provide better support for adaptation and fitness effects in hypothesis testing (e.g., by obtaining complementary measures of energy expenditure, demonstrating redundancy of function, and performing lifetime/longitudinal analyses). We point to continued progress in the use of MIBs in HEB to better understand the past, present, and future of humans and our closest primate relatives.

## 1 INTRODUCTION

Minimally invasive biomarkers (MIBs) are physiological biomarkers obtained from sample types that minimize degree of disturbance to subjects or participants. Over the past several decades, MIB approaches have advanced considerably and have become widely utilized in the sciences and medicine. In the sciences, MIB application is flourishing in disciplines as diverse as nutrition science (Picó et al., 2019), neurobiology (Clow & Smyth, 2020), and conservation biology (Narayan, 2013). In medicine, MIBs are increasingly measured in surveillance and diagnostic capacities, with a desire to replace and one day eliminate more invasive sampling approaches, such as venipuncture and biopsy tissue collection (Metzler, 2010; Wang et al., 2017).

The field of human and non‐human primate evolutionary biology (HEB) has been a longtime leader in MIB research (for early examples of this work, see Brockman et al., 1995; Campbell, 1994; Ellison, 1988; Knott, 1997; Summers et al., 1983; Worthman & Stallings, 1997). In many respects, this position of leadership has emerged as a necessity for carrying out respectful and sustainable research with some of the world's most vulnerable human and comparative non‐human primate (NHP) populations. Researchers in HEB currently measure MIBs among humans and their close primate relatives in diverse contexts and in countries ranging quite literally from “A” (Argentina: e.g., Corley et al., 2021) to “Z” (Zimbabwe: e.g., Campbell & Mbizo, 2006).

The potential advantages of applying MIBs in HEB research are many. Most obviously, MIBs can be obtained with low burden to participants and minimal disruption to their everyday lives, often providing the only possible approach for implementing research designs with direct physiological measures (e.g., among young children, populations with cultural aversions such as blood taboos, or wild NHPs). The use of MIBs can also increase overall participation rates, facilitate repeat‐measures sampling, and improve statistical power by minimizing potential confounds in physiological analyses (e.g., by avoiding acute stress responses). Importantly, MIB approaches provide HEB researchers with a suite of alternative sampling methods that can be strategically applied based on study population preference and a range of other context‐specific factors, including practical considerations such as relative financial cost, biohazard risk, biomarker stability, and required specialization of sample collection/handling (see Gildner, 2021a for a decision‐making flow chart example).

The most common sample types currently used for MIB application in HEB are saliva, urine, feces, hair, milk, and finger‐prick dried blood spots (DBS). An overview of these six key sample types is provided in Table 1. Each is unique in its utility. As discussed above, there is no best sample type for all HEB applications, and researchers must select the approach that best suits their specific research questions and context. In‐depth methodological descriptions, critiques, and comparisons of sample types are beyond the scope of this paper but are provided elsewhere (e.g., Behringer & Deschner, 2017; Lindau & McDade, 2008).

**TABLE 1:** Minimally invasive sample types commonly used for MIB analysis in HEB

	Common with NHPs	Time frame of MIB production reflected	Major advantages	Major disadvantages	Key references
Urine	Yes	h‐4 days	Readily available Can be collected from all ages Supports repeated sampling in quantity Low‐risk and inexpensive collection process	Must be frozen during transport and storage Unsuitable for detecting some short‐term hormone patterns (e.g., diurnal rhythms) Values must adjust for concentration of urine	(Behringer et al., 2017a; Gildner, 2021b; Higham et al., 2020)
Saliva	No	<1–5 min	Supports repeated sampling over short intervals Low‐risk and inexpensive collection process	Must be frozen during transport and storage Viscous samples can be difficult to pipette High internal contamination potential with blood and food debris	(Behringer et al., 2017b; Rosenbaum et al., 2018)
Milk	No	30 min‐2 h	Low‐risk and inexpensive collection process Unique for providing direct information on mother‐infant relationships	Variation due to collection time and sample volume not easy to control for	(Muletz‐Wolz et al., 2019; Quinn, 2021)
Feces	Yes	12–72 h	Time‐integrated levels ranging from several hours to several days Relatively easy to collect from NHPs	Must be frozen during transport and storage Moderate disease transmission risk High purification equipment costs for some analyses	(Bahr et al., 2000; Murray et al., 2013; Ziegler & Wittwer, 2005)
DBS	No	min–hours	Finger prick for small volume of blood No centrifugation or immediate freezing required Low external contamination risk from filter paper cards	Additional training requirements Validation required to demonstrate values reflect serum/plasma	(McDade et al., 2007a; Valeggia, 2007)
Hair	Yes	Up to 3 months	Long‐term biomarker index Storage at room temperature long‐term Low‐risk and inexpensive collection process	Not always available from all individuals Difficult to distinguish between external and internal exposure Limited reference values	(Esteban & Castaño, 2009; Gao et al., 2013)

The range of topics being addressed by HEB researchers using MIBs is wide and expanding. Table 2 highlights some of the physiological systems and traits/evolutionary questions that are being investigated, as well as many of the MIBs that are being utilized. An extended table that includes additional MIBs and individual study entries, species names, and other details is provided in Table S1. At the fundamental level, MIBs are employed to document variation in key aspects of human and comparative NHP biology and phenotype (e.g., reproduction, immune function, stress, and aging). Increasingly, MIBs are also used to operationalize and test hypotheses directly relating to human evolution.

**TABLE 2:** MIBs commonly used in HEB research

MIB	Sample type	Species	Physiological system	Investigated trait/evolutionary topic	Example studies
8‐hydroxy‐2′‐deoxyguanosine (8‐OHdG)	Urine	Humans NHPs	DNA repair	Aging; longevity; oxidative stress	(Gangestad et al., 2010; González et al., 2020; Pontzer et al., 2015; Ziomkiewicz et al., 2016)
Alpha amylase	Saliva	Humans NHPs	Digestion	Fetal growth; stress response	(Broche et al., 2019; Giesbrecht et al., 2015; Petrullo et al., 2016)
Cortisol	Feces Fingernail Hair Milk Saliva Urine	Humans NHPs	Stress; energy regulation	Aggression; competitive response; developmental plasticity; energy allocation; energy balance; growth rate; immune response; life history trade‐offs; stress; stress response	(Emery Thompson et al., 2010; Flinn & England, 1997; Hinde et al., 2015; Nepomnaschy et al., 2006; Urlacher et al., 2018b; Von Rueden et al., 2014)
C‐peptide	Urine	Humans NHPs	Energy regulation	Aging; energy balance; fecundity; hydration	(Ellison & Valeggia, 2003; Emery Thompson et al., 2012; Higham, Girard‐Buttoz, et al., 2011; Reiches et al., 2014; Sherry & Ellison, 2007b; Valeggia & Ellison, 2009a)
C‐reactive protein (CRP)	DBS Feces Saliva Urine	Humans NHPs	Immunity	Energy allocation; inflammation; life history evolution	(Blackwell et al., 2010; Clancy et al., 2013; Lynn et al., 2020; McDade et al., 2008; Thompson et al., 2019; Urlacher, Ellison, et al., 2018)
Creatinine	Urine	Humans NHPs	Cellular metabolism	Muscle mass; energy allocation	(Alvarado et al., 2015; Emery Thompson et al., 2016)
Dehydroepiandrosterone sulfate (DHEAS)	Hair Saliva Urine Milk	Humans NHPs	Energy regulation	Adrenarche; developmental plasticity	(Behringer et al., 2012; Helfrecht et al., 2018; Keestra et al., 2021)
Epstein–Barr virus antibody (EBV‐Ab)	DBS	Humans	Immunity	Energy allocation; cell‐mediated immunity; life history evolution	Eick et al., 2016; Urlacher, Ellison, et al., 2018)
Estradiol	DBS Feces Saliva Urine	Humans NHPs	Reproduction	Energy allocation; fecundity; life history trade‐offs; menarche; ovarian function; stress	(Jasienska et al., 2005; Núñez‐de la Mora et al., 2007b; Thompson & Lampl, 2013; Valeggia, 2007; Worthman & Stallings, 1997)
Immunoglobulin E (IgE)	DBS	Humans	Immunity	Adaptive immunity; energy allocation; helminth defense; life history evolution	(Blackwell et al., 2010; Cepon‐Robins et al., 2021; Urlacher et al., 2021b; Urlacher, Ellison, et al., 2018)
Immunoglobulin G (IgG)	DBS	Humans	Immunity	Adaptive immunity; energy allocation; life history evolution	(Urlacher et al., 2019b; Urlacher et al., 2021a; Urlacher, Ellison, et al., 2018)
Ketones	Urine	Humans NHPs	Metabolism	Energy balance; fat metabolism	(Knott, 1998; Naumenko et al., 2020)
Leptin	DBS	Humans	Energy regulation	Energy allocation; developmental plasticity; immunity	(Miller et al., 2006; Sharrock et al., 2008)
Luteinizing hormone	DBS Urine	Humans	Reproduction	Energy allocation; reproductive investment	(Trumble et al., 2010; Worthman & Stallings, 1997)
Neopterin	Feces Urine	NHPs	Immunity	Disease detection; food competition; innate immunity; mate competition	(Behringer et al., 2017b; Heistermann & Higham, 2015; Higham et al., 2015; Sacco et al., 2020)
Oxytocin	Milk Saliva Urine	Humans NHPs	Neurobehavior	Aggression; cooperation; gonadal function; pair‐bonding; parental investment	(Crockford et al., 2013; De Dreu & Kret, 2016; Wittig et al., 2014)
Progesterone	Feces Saliva Urine	Humans NHPs	Reproduction	Aging; energy allocation; Fecundity; ovarian function; life history; menarche; stress response	(Clancy et al., 2013; Ellison et al., 1986; Jasieńska & Ellison, 1998; Núñez‐de la Mora et al., 2007a; Valeggia & Ellison, 2004; Vitzthum et al., 2002)
Prolactin	DBS Urine	Humans NHPs	Reproduction	Milk production; Paternal care	(Gettler et al., 2015; Worthman & Stallings, 1997; Ziegler et al., 2004)
Secretory immunoglobulin A (sIgA)	Milk Saliva	Humans	Immune	Immunocompetence; offspring immune programming	(Klein et al., 2018; Lynn et al., 2020; Miller & McConnell, 2015)
Telomere length	DBS Saliva Skin	Humans NHPs	Cellular proliferation	Aging; energy allocation; longevity; stress response	(Eisenberg et al., 2012; Goldman et al., 2018; Rej et al., 2020; Rej et al., 2021; Steinert et al., 2002)
Testosterone (T)	DBS Feces Hair Saliva Urine	Humans NHPs	Energy regulation; Reproduction	Aggression; Developmental plasticity; Energy allocation; fecundity; immune activation; male reproduction; mate competition; reproductive success; stress response	(Alvarado et al., 2019; Bribiescas, 1996; Ellison & Panter‐Brick, 1996; Emery Thompson, et al., 2012; Gettler et al., 2011b; Kuzawa et al., 2009; Muehlenbein & Watts, 2010; Muller et al., 2009; Trumble et al., 2010)
Triiodothyronine (T3)	Feces Urine	NHPs	Energy regulation; Thermoregulation	Energy allocation; energy balance; thermoregulation	(Chen et al., 2021; Cristóbal‐Azkarate et al., 2016; Dias et al., 2017; Gesquiere et al., 2018; Thompson et al., 2017)

*Note*: Selected examples (non‐comprehensive list). An extended list that includes additional MIBs and individual study entries, species names, and other details is provided in Table S1.

This paper provides an overview of the use of MIBs in HEB. Our aim is not to provide a comprehensive review of this expansive area of research. Rather, we have three more specific objectives: (1) To highlight key research topics which successfully utilize MIBs; (2) To identify promising yet under‐investigated areas of MIB application; (3) To discuss current challenges in MIB research, with suggestions for advancing the field. Our focus is on the topics that MIBs are utilized to address, not on the range of potential applications for specific biomarkers. Included evolutionary topics were chosen based on implications for the field, coverage in the literature, and our own research interests. For recognition of the broader range of evolutionary topics being explored using MIBs in HEB, see Table 2.

## 2 KEY HEB TOPICS INVESTIGATED USING MIBS

### 2.1 Energetics and life history variation/evolution

Much has been written on human and comparative NHP energetics and their importance for understanding human phenotypic variation and evolutionary processes (e.g., Ellison, 2017; Emery Thompson, 2017; Pontzer, 2017; Urlacher et al., 2019a). This area of research is deeply rooted in life history theory. Life history theory is built on the premise that organisms evolve under selective pressure to allocate a limited supply of time and energy (i.e., calories) to competing demands across the life course in a manner that maximizes fitness (Charnov, 1991; Stearns, 1992). This simple yet powerful framework – predicting trade‐offs between key physiological life tasks as a function of age, environment, and other factors – has been used to shed light on human patterns of growth (Bogin et al., 2007; Leigh, 2001; Urlacher, Ellison, et al., 2018; Urlacher & Kramer, 2018), reproduction (Bribiescas, 2010; Ellison et al., 1993; Emery Thompson et al., 2007; Valeggia & Ellison, 2009b), immune function (Blackwell et al., 2011; Cepon‐Robins et al., 2019; Gurven et al., 2009; McDade, 2003; Muehlenbein & Bribiescas, 2005), physical activity (Caldwell, 2016; Lieberman et al., 2021; Shattuck & Muehlenbein, 2015), aging (Crimmins & Finch, 2006; Gurven et al., 2008; Hawkes, 2003; Jones, 2011; Kaplan et al., 2000; Ziomkiewicz et al., 2016), and other traits.

Two variables that are central to the life history theory approach for understanding phenotypic variation and evolution are energy condition (i.e., energy availability or balance) and energy allocation (i.e., the distribution of energy investment between competing life tasks). Accordingly, these variables have received considerable attention in HEB, with researchers finding success in developing and utilizing MIBs for both. This application of MIBs provides opportunities to objectively assess energetic parameters in field settings. The use of metabolic hormones as MIBs in this area also facilitates direct investigation of the physiological pathways linking environmental stimuli with phenotypic variation (Bribiescas & Ellison, 2008), providing novel opportunity to test mechanistic hypotheses and to increase the sensitivity of life history analysis. Here, we describe several common MIBs of energy condition and allocation in HEB and overview their current application.

#### 2.1.1 C‐peptide

C‐peptide is widely applied in HEB as a MIB of energy balance. The hormone insulin, owing to its role directing glucose homeostasis and the synthesis/break‐down of energy reserves, is a primary regulator of metabolism and a sensitive marker of energy balance when measured at physiological baseline. C‐peptide is a pancreatic byproduct of insulin production that is generated in equimolar fashion to insulin itself. Importantly—and in contrast to insulin—C‐peptide is largely inert in circulation and has a relatively long half‐life, meaning that circulating concentrations of C‐peptide reflect daily insulin production better than circulating insulin itself (Kruszynska et al., 1987). C‐peptide, but not insulin, is also excreted intact in urine at a rate directly proportional to production (Kruszynska et al., 1987). For these reasons, urinary C‐peptide serves as a reliable MIB of insulin production and, thus, energy balance (Emery Thompson, 2017; Sherry & Ellison, 2007a).

The practicalities of C‐peptide analysis in urine samples collected in field settings have been thoroughly described (Emery Thompson & Knott, 2008; Higham, Girard‐Buttoz, et al., 2011). Although 24‐h urine sampling is recommended, opportunistic collection of urine also provides reliable C‐peptide estimates of energy balance in research contexts, particularly when utilizing multi‐day sampling. Lab analysis is typically done with enzyme‐linked immunosorbent assay (ELISA) or radioimmunoassay (RIA) and can be performed with conventional urine samples (possessing good long‐term stability of C‐peptide when continuously frozen) or urine dried and stored on filter paper (Emery Thompson & Knott, 2008; Higham, Girard‐Buttoz, et al., 2011; Reiches et al., 2014). A key limitation of C‐peptide analysis is that available assays lack standardization, making it particularly difficult to compare data across assays and labs (Little et al., 2008).

The use of urinary C‐peptide as a MIB of energy balance has been validated in the lab and field among humans (see above) and a range of comparative wild and captive NHPs, including chimpanzees (*Pan troglodytes*: Emery Thompson et al., 2009; Sherry & Ellison, 2007a), bonobos (*Pan paniscus*: Deschner et al., 2008), and several other apes and monkeys (Emery Thompson & Knott, 2008; Fürtbauer et al., 2020; Girard‐Buttoz et al., 2011; Grueter et al., 2014a; Harris et al., 2010; Sacco et al., 2021; Sherry & Ellison, 2007a). Diverse questions have been addressed. A strength, however, has been research relating to female reproductive ecology (Ellison, 2003a) and the study of reproductive variation using energetic models that link environmental conditions with life history trade‐offs.

C‐peptide has proven a valuable tool in female reproductive ecology in large part because insulin itself acts both directly and indirectly (via gonadotropins) to regulate ovarian steroid hormone production (Greisen et al., 2001). Thus, C‐peptide reflects a physiological link between environment and reproductive function/fecundity (Sherry & Ellison, 2007a). The earliest work utilizing C‐peptide as an MIB in this framework investigated the drivers of variation in human reproductive rate. In a series of analyses by Valeggia and Ellison among the Indigenous Qom of Argentina (Ellison & Valeggia, 2003; Valeggia & Ellison, 2001; Valeggia & Ellison, 2004; Valeggia & Ellison, 2009b), urinary C‐peptide tracked across postpartum intervals was used to demonstrate that maternal energy balance declines sharply with birth and the initiation of lactation, recovers to pre‐lactation levels gradually as moderated by maternal resource availability, and is closely tied to ovarian steroid hormone (estradiol and progesterone) production and the resumption of ovulation. In sum, this work used C‐peptide to demonstrate the role of energy balance in the presumed adaptive regulation of fecundity and birth spacing among humans.

Supporting comparative evidence linking urinary C‐peptide levels with reproductive function is now also available from wild chimpanzees (Emery Thompson et al., 2012), chacma baboons (*Papio ursinus*: Fürtbauer et al., 2020), and howler monkeys (*Alouatta palliata*: Rangel‐Negrin et al., 2021). The role of resource availability in moderating rapid recovery of postpartum C‐peptide levels and ovulation among humans (Valeggia & Ellison, 2004), for example, has now also been demonstrated among chimpanzees living with varying food resources (Emery Thompson et al., 2012). Highlighting the importance of variation in energy expenditure—as well as energy intake—in this energy balance pathway, the dynamics of postpartum urinary C‐peptide levels have also been found to map onto the dynamic costs of milk production and infant transport and vigilance activities among NHPs (Emery Thompson et al., 2012; Rangel‐Negrin et al., 2021). The interesting finding that C‐peptide levels are greatest during gestation among humans and many NHPs (Fürtbauer et al., 2020; Rangel‐Negrin et al., 2021) has led to the hypothesis that primates have evolved a conservative pregnancy metabolic strategy to prepare for the heightened challenges of lactation (Dufour & Sauther, 2002; Fürtbauer et al., 2020). More research is needed in this area to understand the energetic trade‐offs that underlie this potential adaptation, with preliminary work investigating urinary C‐peptide among Sanje mangabeys (*Cercocebus sanjei*) indicating the likely importance of trade‐offs involving immune activity (McCabe et al., 2016).

#### 2.1.2 Cortisol

Likely no MIB has received more attention in HEB than cortisol. While its biomarker value is wide, cortisol is often utilized as a MIB of both energy condition and energy allocation toward critical life tasks. Cortisol is a glucocorticoid steroid hormone produced by the cortex as the primary end product of the hypothalamic–pituitary–adrenal axis (Dickerson & Kemeny, 2004; Sapolsky et al., 2000a). Triggered routinely for release by a range of physical and psychosocial “stressors”, including low energy intake and physical activity, cortisol's primary physiological role is to maintain energy homeostasis. To accomplish this, cortisol stimulates catabolic metabolism (i.e., converts energy stores into usable energy) and downregulates a number of costly biological systems, including reproduction, immunity, and growth (Gunnar & Quevedo, 2007). The sensitivity of cortisol production to energetic stress makes it a potential biomarker of energy condition/balance. At the same time, the regulatory effects of cortisol on multiple key life tasks make it a useful biomarker of energy allocation in the study of life history trade‐offs.

Researchers in HEB measure cortisol as a MIB in many sample types, including saliva, urine, hair, feces, and milk. Commercial ELISA and RIA kits are typically used for lab assessment. Detailed reviews of the nuances and limitations of cortisol measurement are provided elsewhere (Cook, 2012; Nicolson, 2008; Saxbe, 2008). Here, two major factors warrant mention. First, cortisol has a strong pulsatile and diurnal pattern of secretion among humans and likely most other primates (Adam & Kumari, 2009; Gröschl et al., 2003). Second, and relatedly, sampling strategy (e.g., saliva vs. hair, 24‐h urine vs. single‐point urine) can have a large impact on the timescale of cortisol production ultimately measured. Point measures of salivary cortisol, for example, are particularly susceptible to the diurnal and pulsatile nature of cortisol secretion and are therefore unlikely to reflect daily cortisol production. The physiological implications of acute cortisol activity differ from those of chronic activity (Do Yup Lee & Choi, 2015, see Developmental Plasticity below), and it is therefore critical that appropriate sampling approaches are selected. Diverse cortisol sampling approaches in HEB make comparisons across studies particularly challenging (Anestis, 2010; Anestis & Bribiescas, 2004).

Cortisol is routinely applied in HEB as a MIB of energy condition, specifically as a MIB of negative energy balance or energy “stress”. This use is derived from extensive experimental evidence from humans (Nieuwenhuizen & Rutters, 2008) and has been supported with comparative evidence from NHPs demonstrating that cortisol in feces, urine, and hair is elevated in contexts of low energy availability, including poor habitat quality (Busch and Hayward, 2009; Jaimez et al., 2012; Rangel‐Negrín et al., 2009) and seasonal food scarcity (Gesquiere et al., 2008; Pride, 2005). As expected, the impact of low energy availability on cortisol appears to be greatest among those already at risk for negative energy balance, such as lactating females (Emery Thompson, 2009; Foerster et al., 2012). It is important to note that while the above studies and others provide a strong basis for the use of cortisol as a MIB of energy condition, factors unrelated to energetic stress, such as psychosocial stress, may also influence cortisol production and introduce error into energetic analyses (Emery Thompson, 2017; Flinn, 2010).

To test life history hypotheses, HEB researchers also use cortisol as a MIB of energy allocation. This work capitalizes on the integrated regulatory role of cortisol across major physiological systems. Under conditions of energetic stress, the evolved function of cortisol is seemingly to prioritize fueling essential life tasks (i.e., those that are critical for survival or most immediate to fitness) at the cost of diverting energy away from tasks that are relatively low priority (Bribiescas, 2010; Charmandari et al., 2005; Ellison, 2017). Thus, cortisol is viewed as a mediator of energetic trade‐offs that responds to environmental energy cues. Using cortisol as a MIB under this life history framework, researchers have identified presumed adaptive negative relationships between cortisol and human growth (Nyberg et al., 2010, see Developmental Plasticity below), reproduction (Foerster et al., 2012; Lodge et al., 2013; Valeggia & Ellison, 2009b), immune activity (Hoffman et al., 2011; Muehlenbein, 2006), and other physiological tasks. Notably, fecal cortisol among ring‐tail lemurs (*Lemur catta*: Pride, 2005) and hair cortisol among gray mouse lemurs (*Microcebus murinus*: Rakotoniaina et al., 2017) have also been shown to predict mortality rate, providing comparative evidence that trade‐offs mediated by cortisol are not always enough to ensure survival. As noted by Beehner and Bergman in their excellent review (2017), studies directly testing relationships between cortisol and clear outcomes of fitness are rare among primates, preventing strong conclusions about the adaptive significance of observed energy allocation trade‐offs in HEB.

#### 2.1.3 Triiodothyronine (T3)

The thyroid hormone triiodothyronine (T3) has also received attention in HEB as a MIB of energy condition and energy allocation directed to basal metabolic tasks, specifically thermoregulation. Circulating T3 is secreted along with its less biologically active precursor thyroxine (T4) by the action of the hypothalamic–pituitary‐thyroid axis. Following additional conversion of T4 to T3 in many peripheral tissues, T3 acts at the cellular level to upregulate ATP production, ultimately increasing cellular metabolic rates and thermogenesis (Danforth & Burger, 1989). Given this direct stimulatory action on many tissues throughout the body (e.g., adipose, hepatic, skeletal muscle), as well as indirect insulin sensitivity‐promoting effects (López et al., 2010), T3 levels broadly reflect basal metabolic rate (BMR) among mammals (Leonard et al., 2005) and energy availability and energy balance among humans (López et al., 2013). The regulatory actions of T3 on numerous physiological functions also make it an attractive biomarker of energy allocation, primarily toward thermoregulation (Behringer et al., 2018; Leonard et al., 2005; Mantzouratou et al., 2022).

Traditional assessment of T3 relies on venipuncture blood samples. However, T3 measurement is also validated in urine and feces among humans (Burke et al., 1972; Yoshida et al., 1980) and NHPs (Gesquiere et al., 2018; Schaebs et al., 2016; Wasser et al., 2010). Lab measurement is typically performed using ELISA or RIA. Circulating T3 mirrors cortisol's dynamic diurnal rhythm (Nicoloff et al., 1970). As such, T3 concentrations in urine and feces are often poorly correlated with blood levels (Burke & Shakespear, 1976; Shakespear & Burke, 1976) and reflect longer‐term T3 production (Behringer et al., 2018). Importantly, urinary and fecal T3 measures must account for the presence of both bound and unbound forms of T3 as well as various T3 metabolites that may differ in their physiological significance (Burke & Shakespear, 1976). For these reasons, urinary and fecal T3 assessment must be approached cautiously.

Researchers in HEB capitalize on T3's role as a regulator of BMR to use T3 as a MIB of energy allocation to basal metabolism in life history analyses. Fecal T3 is regularly measured to investigate energetic response to limited environmental food availability, with the positive relationship between T3 and energy availability/consumption thought to reflect adaptive regulation of basal metabolism to avoid overspending during periods of energy shortage (Cristóbal‐Azkarate et al., 2016; Gesquiere et al., 2018; Schaebs et al., 2016; Wasser et al., 2010).

Researchers in HEB have also used T3 to investigate energy allocation to more specific physiological tasks. Thermoregulation has received the most attention in this area, building on work (using predominantly venipuncture samples) indicating that population variation in human metabolic heat production can be explained as T3‐mediated adaptation to environmental temperature and cold stress (Cepon et al., 2011; Leonard et al., 2002; Leonard et al., 2005; Levine et al., 1995; Roberts, 1952). Using fecal T3 data, supporting comparative evidence is now available from several species of NHPs (Chen et al., 2021; Gesquiere et al., 2018; Thompson et al., 2017). Interestingly, this research and other work with NHPs (Dias et al., 2021) also demonstrates a positive relationship between fecal T3 levels and male mating effort, a finding that provides comparative evidence for influence of T3 on energy allocation specifically to male reproduction.

#### 2.1.4 Testosterone (T)

Testosterone has been widely used in HEB as a MIB of energy allocation, specifically energy investment in male reproduction. An androgenic steroid hormone, T is produced in males in the testes as the final product of the hypothalamic‐ pituitary‐testicular axis. Its primary function is anabolic in driving the development and sustainment of male sex characteristics, including spermatogenesis and the synthesis of metabolically costly skeletal muscle tissue (Nieschlag et al., 2012). However, T has broad metabolic functions that also involve the regulation of growth, immune activity, behavior, and other physiological systems. Testosterone's regulatory actions, coupled with its wide observed variation in concentration between species, populations, and individuals (Bribiescas, 2010; Ellison et al., 2002; Schurmeyer & Nieschlag, 1982), make it a key biomarker of energy allocation to male reproduction.

Testosterone can be measured in many sample types, and researchers in HEB currently utilize T as a MIB predominantly in saliva, urine, hair, feces, and milk. This research spans several decades and is being performed among diverse groups of humans and comparative NHPs. The measurement of T is typically performed using commercial ELISA or RIA, but not without limitations. Similar to cortisol, T concentration is labile in circulation, is highly dependent on sample type, and is subject to diurnal, pulsatile, and other biological rhythms (for complete discussion of these issues, see Trost & Mulhall, 2016; van Anders et al., 2014). Importantly, T exists in bound and unbound forms at different levels in different sample types (Keevil et al., 2016) and appears to be particularly susceptible to environmental/behavioral modulation of the relationship between physiological production and clearance (Cadoux‐Hudson et al., 1985), influencing measurement. The analytical assessment of T is plagued by a relatively high degree of between‐assay and between‐lab variation (Rosner et al., 2007). For these reasons, particular care must be taken when assessing T, particularly when making comparisons across studies (Anestis, 2010).

In contrast to several other energetic MIBs common in HEB, T is not a reliable indicator of energy condition. Studies of diet among humans living in non‐industrialized populations (e.g., Alvarado et al., 2019; Ellison & Panter‐Brick, 1996) and among wild chimpanzees (Muller & Wrangham, 2005) and orangutans (Knott, 1999) demonstrate that T in saliva, urine, and hair is not consistently related to BMI and that short‐to‐intermediate periods of low energy availability have no detectable impact on T. It is generally only with complete fasting that notable decreases in T have been reliably detected among humans (Klibanski et al., 1981; Röjdmark, 1987; Trumble et al., 2010). The impact of regular physical activity on T also appears to be marginal, such that seasonal increases in everyday physical activity are not associated with changes in salivary T among humans (Bentley et al., 1993; Ellison & Panter‐Brick, 1996; although see evidence for some changes in diurnal rhythms in Vitzthum et al., 2009). Collectively, this work has led to the conclusion that typical acute and seasonal variation in energy availability and balance has little impact on T (Emery Thompson, 2017; Muehlenbein & Bribiescas, 2010).

The value of T as a MIB of energy allocation to male reproductive effort is much more established. While sperm production itself accounts for <1% of BMR among mammals (Elia, 1992), T also drives male reproductive effort directed toward mate acquisition, including behavioral characteristics that can be energetically costly (e.g., competitiveness, aggressiveness, territoriality, libido) and skeletal muscle mass that is expensive to synthesize and maintain. These costs are substantial. Among male NHPs, it has been estimated that the lifetime energetic costs associated with T's somatic effects alone are equivalent to the direct lifetime reproductive costs experienced by females (i.e., energy invested in pregnancy, lactation, etc., Key & Ross, 1999). The mate‐acquisition behaviors promoted by T also appear to incur substantial energy costs for many primates (Alberts et al., 1996; Eberle & Kappeler, 2004; Muller & Wrangham, 2004c). The energetic cost of male reproduction is clearly considerable, and it is regulated by T (Bribiescas, 2001; Muller, 2017). This recognition is applied widely in HEB to test life history theory predictions.

Work in HEB has shown that salivary T among adult males from human subsistence populations in Africa (e.g., Christiansen, 1991; Ellison et al., 1989; Gray et al., 2007), South America (e.g., Beall et al., 1992; Bribiescas, 1996; Trumble et al., 2012), Asia (e.g., Ellison et al., 2002; Ellison & Panter‐Brick, 1996), and Oceania (e.g., Campbell, 1994), while exhibiting between‐group variation (Alvarado et al., 2019; Ellison et al., 2002), are consistently lower than those of men living in industrialized populations. Wild male chimpanzees similarly have lower urinary T than their captive counterparts (Muller & Wrangham, 2005). A wide body of research is investigating the specific environmental factors that underlie such broad differences in reproductive energy allocation and their potential adaptive significance. In addition to the possible effect of chronic energy limitation during ontogeny in establishing low lifetime T (see Developmental Plasticity below, Bribiescas, 2010), energetic trade‐offs involving metabolic tasks in direct competition with immediate reproduction appear likely.

Research using T as a MIB in HEB has provided considerable evidence for trade‐offs between reproduction and immune activity (see reviews in Muehlenbein & Bribiescas, 2005; Muehlenbein et al., 2017). However, T's impact on immunity appears to be complex (i.e., regulating specific aspects of immunity unevenly [Braude et al., 1999; Ezenwa et al., 2012]) and can be difficult to isolate from other environmental factors in field settings (Prall & Muehlenbein, 2014). Such analyses must be approached carefully. Other key work in this area uses T as an MIB to investigate trade‐offs between reproductive effort and somatic maintenance (e.g., cellular repair), with an emphasis on understanding the accelerating impact of T on biological senescence and, ultimately, on reproductive effort's effects on the evolution of human and primate lifespans (Altmann et al., 2010; Bribiescas, 2020; Kruger & Nesse, 2006). A final noteworthy topic is the use of T as an MIB to investigate trade‐offs underlying variation in human paternal care (see reviews in Gettler, 2020; Gettler et al., 2014; Gray & Campbell, 2009; Gray et al., 2020). In this work, lower T among high paternal‐care populations (Muller et al., 2009), among high paternal‐care fathers (Alvergne et al., 2009; Gray, 2003; Kuzawa et al., 2009), and following men's own initiation of fatherhood (Gettler, 2016; Gettler et al., 2011a) is interpreted as directing energy toward investment in current offspring and away from mate acquisition. Similar comparative evidence is available for several NHPs (Bales et al., 2006; Ziegler & Wittwer, 2005). In sum, the use of T as an MIB of energy allocation in HEB has provided insights into the factors driving species, population, individual, and lifetime variation in reproductive effort.

### 2.2 Developmental plasticity

Human evolutionary biologists and anthropologists have been interested in how early life conditions shape human development and lifelong phenotype for well over a century (Boas, 1912; Chisholm et al., 1993; Ellis et al., 2009; West‐Eberhard, 2003b). Within HEB, developmental plasticity—the phenomenon by which a single genotype can produce distinct phenotypes in response to variation in early environmental conditions (Lea et al., 2017)—remains a key concept and a focus of research on the evolutionary mechanisms underlying human diversity.

Current adaptive theories on developmental plasticity in HEB posit that individuals respond developmentally to variation in experienced conditions in order to facilitate either immediate fitness benefits (e.g., survival, “immediate adaptive responses”) or to facilitate lifetime fitness benefits (e.g., future reproduction, “predictive adaptive responses [PARs]”) (Ellis & Del Giudice, 2019; Gluckman et al., 2005; Kuzawa & Bragg, 2013; Lea et al., 2017). In the case of PARs, it is suggested that evolved biological mechanisms allow individuals to respond to early environmental cues (within genetically‐defined limits and norms of reaction) in order to target their development and adult phenotype onto predicted later life environmental conditions, ultimately increasing fitness (Gluckman et al., 2005). Defining developmental pathways, such as PARs, can improve understanding of variation in phenotype and fitness (Lu et al., 2019) as well as help to identify the underpinnings of individual trajectories of lifetime health outcomes (McDade et al., 2007b).

Many MIB approaches are suited for data collection with individuals of all ages and developmental stages. As such, MIBs have been used to investigate developmental plasticity among a wide range of human groups and comparative NHP species (Behringer et al., 2014; Emery Thompson et al., 2012; Gesquiere et al., 2005; Tkaczynski et al., 2020). This work spans several active areas of HEB research on developmental plasticity, including studies on response to early life adversity, life history flexibility, and biosocial pathways of variation in development and health. Problematically, most HEB research on developmental plasticity remains limited to retrospective investigation of prior ontogenetic responses using data collected from adults. Relatively few studies have utilized longitudinal MIB approaches to track trait specific variation in association with early life conditions. This dearth of longitudinal data poses considerable challenges for identifying adaptation and fitness effects.

Here, we overview several MIBs used in HEB to investigate developmental plasticity and highlight their key research applications and potential for expanded use.

#### 2.2.1 Cortisol

Cortisol is again used in HEB as a MIB of experienced “stress” and a mediator of adaptive phenotypic response in investigations of developmental plasticity. Cortisol is released as a result of various stressors, including energetic challenges and adverse social situations (Lu et al., 2019). Its regulatory roles can be applied to understand developmental plasticity, whereby cortisol production in high‐stress early environments may serve to influence development and adult phenotype in response signals of a challenging lifetime environment.

Several studies in HEB have assessed cortisol levels among pregnant women or in breast milk as a MIB of experienced maternal stress. In the adaptive framework, maternal cortisol is hypothesized to transmit signals of environmental quality to the fetus, resulting in calibrated developmental plasticity that enhances immediate survival and long‐term fitness. Support for this hypothesis in HEB has been found in humans demonstrating that elevated maternal evening salivary cortisol levels are associated with reduced fetal growth rate (Thayer et al., 2012). Similar comparative evidence is available from NHPs (Bardi & Huffman, 2006; Berghänel et al., 2016; Mustoe et al., 2014). In breast milk, cortisol concentrations may reflect the maternal environment and their own experienced life history (Hinde et al., 2015). A growing body of evidence among humans (Grey et al., 2013; Hahn‐Holbrook et al., 2016) and NHPs (Bernstein & Hinde, 2016; Dettmer et al., 2018; Hinde et al., 2015; Hinde & Milligan, 2011; Petrullo et al., 2019; Sullivan et al., 2011) indicates that milk cortisol levels influence offspring development as predicted by evolutionary theory. This work supports cortisol's key role as a signal of environmental stress to offspring in the postnatal period, supporting adaptive PAR theory in which offspring respond early in life to environmental signals to optimize lifetime phenotype and fitness.

Cortisol is also used as a MIB of an individual's own experienced stress in HEB developmental plasticity research. Among children in the subsistence Tsimane population of Bolivia, for example, elevated morning and evening salivary cortisol levels—themselves driven by immunological stress—were found to be predictive of current child growth outcomes (Nyberg et al., 2012). Retrospectively, many studies in HEB using saliva, urine, feces, and hair samples have also identified relationships linking early life adversity among humans (e.g., low socioeconomic status, childhood trauma, familial stress) with cortisol patterns in adulthood (Desantis et al., 2015; Kim et al., 2020; Zhang et al., 2019). Similar relationships among wild chimpanzees (Tkaczynski et al., 2020) provide comparative evidence that socioecological challenges during early life (e.g., feeding competition, poor maternal care, parent‐offspring conflict) alter cortisol levels across development. Chronic stress exposure among humans and NHPs results, somewhat unintuitively, in diminished rather than elevated cortisol production, including lower overall cortisol levels and blunted reactivity and diurnal rhythms (Ellis & Del Giudice, 2019; Ponzi et al., 2020; Sapolsky et al., 2000b). This downregulation in salivary cortisol production has been demonstrated at the population level among subsistence human groups subject to chronic stressors early in life (Nyberg, 2012; Urlacher et al., 2018c). Cortisol suppression in the presence of chronic stress may function as an adaptive mechanism to maintain physiological homeostasis and prevent deleterious outcomes of hypercortisolism, such as persistent wasting and immunosuppression (Nyberg, 2012; Urlacher et al., 2018c). This hypothesis warrants further investigation.

#### 2.2.2 Progesterone

Developmental plasticity provides an important framework for understanding variation in lifetime reproductive phenotypes (Jasienska et al., 2017). In HEB, progesterone has often been used as an MIB of reproductive effort in developmental plasticity analysis. Progesterone is an ovarian pregnane steroid hormone that plays important roles in female sexual development, ovarian function, pregnancy, lactation, and other reproductive activities (Jasienska et al., 2017; Nunez‐de‐la‐Mora & Bentley, 2008). It can be measured using commercial ELISA and RIA kits directly in saliva, hair, and DBS and indirectly via metabolites in urine (e.g., pregnanediol glucuronide [PdG]) and feces (e.g., 5α‐pregnane‐3α‐ol‐20‐one [5‐P‐3OH]). A full review of progesterone measurement is provided elsewhere (Gildner, 2021a). Progesterone production varies predictably across the menstrual cycle, with levels increasing during the luteal phase to prepare the uterus for implantation and support conception (Taraborrelli, 2015). Progesterone concentration is also influenced acutely by a number of other factors, including diet and exercise (Ellison, 2003b). As such, repeat measures of progesterone on multiple days across the menstrual cycle and over multiple cycles is typically needed to reliably document habitual patterns, particularly when measuring the hormone in saliva (Jasienska & Jasienski, 2008).

Adult progesterone levels appear to be influenced by early life environmental conditions (Jasienska et al., 2017), and researchers in HEB have utilized progesterone extensively as an MIB to investigate developmental plasticity in human female reproductive investment (Ellison et al., 1986; Lipson & Ellison, 1992; Núñez‐de la Mora et al., 2007a; Vitzthum et al., 2002). From a life history perspective, progesterone adjustments can be adaptive in regulating reproductive investment and probability of conception as a function of environmental cues on energy availability and social support or extrinsic mortality (Ellison, 1996; Ellison, 2003b). This approach has proven useful for understanding variation in reproductive function in women across several populations, including among rural Polish farmers (Jasienska & Ellison, 2004), Bangladeshi migrants (Núñez‐de la Mora et al., 2007a), and rural Bolivian Aymara women (Vitzthum et al., 2004). Among Bangladeshi migrants to London, for example, it was shown that women who migrated during childhood have higher levels of average luteal salivary progesterone than women who never migrated and continue to reside in Bangladesh (Núñez‐de la Mora et al., 2007a). Illustrating the importance of timing on developmental plasticity in ovarian function, those women who migrated earliest in development displayed the highest salivary progesterone levels and experienced the earliest age of menarche (Núñez‐de la Mora et al., 2007a). Supporting evidence is available from several species of NHPs, demonstrating the impact of a broader range of early‐life socioecological variables (e.g., maternal dominance rank, natal dispersal) on adult progesterone levels and variation in female reproductive function (Gesquiere et al., 2005; Onyango et al., 2013; Sousa & Ziegler, 1998; Xia et al., 2018).

#### 2.2.3 Testosterone (T)

As described above, T is a commonly utilized MIB of male reproductive effort in HEB. Within the context of developmental plasticity, studies in HEB have utilized T to understand how socioecological conditions experienced during early life also influence male reproductive investment across the lifespan. This body of research is more limited than for cortisol or progesterone but is still worth mention. Most of the work on this topic exists for humans (Alvergne et al., 2008; Magid et al., 2018; Thompson & Lampl, 2013; Trumble et al., 2013), and supports the hypothesis that variation in energetic factors (i.e., nutritional intake, pathogen load) during development adaptively influences adult reproductive investment (i.e., timing of reproductive maturity, adult reproductive function). For example, it has been shown that Bangladeshi men who migrated to London during childhood display higher levels of waking and evening salivary T levels and experience earlier pubertal onset than men who completed their childhood in Sylhet (Magid et al., 2018). Importantly, this pathway may help to more broadly explain the lower T levels documented among subsistence populations relative to industrialized references (see *Energetics and Life History Variation/Evolution*). Comparative NHP data on this topic is lacking, and more research is needed to identify the specific socioecological variables that drive lifetime variation in T and male reproductive function.

### 2.3 Social status and dominance

Research on social dynamics has a rich history in HEB, including the study of human and NHP social stratification and hierarchies (Hawley, 1999; Sapolsky, 2005). Minimally invasive biomarkers are widely used to operationalize and test evolutionary hypotheses in this area (Harris & Schorpp, 2018). Two related traits have received a particularly large amount of attention: social status among humans and social dominance among NHPs. Human social status is defined as relative access to resources and is a function of how members of a social group evaluate one another based on prestige or influence (Henrich & Gil‐White, 2001). It is linked to differences in skill, respect, and power (Berger et al., 1972; Berger et al., 1980; Desjardins et al., 2015). Comparatively, in NHPs, social dominance is more broadly defined as a relative measure of “coexistence” among members of a social group, where coexistence is determined by resource acquisition, reproduction, and health (Dewsbury, 1982; Drews, 1993; Majolo et al., 2012; Sapolsky, 2005). Social dominance is usually represented in terms of social rank and can be applied to individual and dyadic relationships through dominance hierarchies that mediate interactions such as conflict and competition (Newton‐Fisher, 2017).

Given the importance of social status and dominance as traits for understanding social dynamics and related evolutionary processes, researchers in HEB have developed several MIB approaches to investigate them. Here, we highlight three of the most studied MIBs in HEB social status and dominance research.

#### 2.3.1 C‐peptide

As outlined above, C‐peptide is widely used in HEB as a MIB of human and NHP energy balance. This utility has proven useful for research investigating the energetic correlates and evolutionary selective pressures surrounding social dominance among many NHPs (Deschner et al., 2008; Emery Thompson et al., 2009; Grueter et al., 2014b; Higham et al., 2011; Sacco et al., 2021; Surbeck et al., 2015). Unfortunately, similar applications with social status among humans, while suggested (Valeggia & Ellison, 2009b), have not been directly performed.

Available C‐peptide MIB research among NHPs consistently indicates that attaining and maintaining social dominance comes with a considerable energetic cost. Among adult male chimpanzees, for example, high‐ranking individuals have lower urinary C‐peptide levels than lower‐ranking individuals across multiple seasons (Emery Thompson et al., 2009). It has been suggested that this apparent energetic cost is related to greater aggression and foraging effort necessary to maintain social dominance (Emery Thompson & Georgiev, 2014). Research among rhesus macaques offers additional insight into the dynamics of social dominance energetics, demonstrating that dominant male urinary C‐peptide levels plummet during periods of heightened mating effort and are inversely related to obtained copulation events (Higham et al., 2011). Together, these findings provide insight into the evolution of primate social dynamics and structure, highlighting the role of social competitiveness strategies and dominance energetics in shaping distributions in male reproductive success.

C‐peptide is also used as a MIB of energy balance to investigate the evolutionary links between primate social relationships and party size. The evolution and nature of grouping patterns in primates is hypothesized to relate, in part, to access to food (Anderson et al., 2002). Providing support for this hypothesis, longitudinal research among bonobos—characterized by fission–fusion social dynamics—has demonstrated a positive association between mean monthly urinary C‐peptide levels and mean party size, indicating larger group formation and reduced hostility in times of greater energy abundance (Surbeck et al., 2015). Interestingly, when in large groups, dominant male bonobos have greater, not lower, C‐peptide levels than their low‐ranking counterparts. This indicates increased contest competition over food, shedding light on the dynamic dominance‐energetic relationships underlying the evolution of primate social systems (Emery Thompson et al., 2009; Surbeck et al., 2015). These findings can be applied to understand the evolution human social systems and indicate the promise of similar application of C‐peptide as a MIB in human research.

#### 2.3.2 Cortisol

Cortisol's role as a MIB of stress has also been applied in HEB to investigate social dynamics. Specifically, cortisol is used to illuminate relationships between social status/dominance and experienced stress. This has led to better understanding of the evolution of human and NHP social systems and life history strategies.

Cortisol levels among humans are well established to have an inverse relationship with social status (Sherman & Mehta, 2020). In HEB, this relationship has now been demonstrated using a range of cortisol and social status measures among small‐scale populations. This work includes research using multi‐point salivary cortisol assessment among adult male villagers in Dominica (Decker, 2000) and subsistence forager‐horticulturalists in Papua New Guinea (Konečná & Urlacher, 2017), as well as research measuring first‐morning urine cortisol among subsistence forager‐horticulturalists of all ages in Bolivia (Jaeggi et al., 2021; Von Rueden et al., 2014). Interestingly, no such relationship was found between social status and hair cortisol concentration among relatively egalitarian female hunter‐gatherers of Tanzania (Fedurek et al., 2020). The totality of evidence, however, from both industrialized and small‐scale populations indicates a typically strong inverse relationship between human social status and cortisol levels. This relationship is widely interpreted to reflect lower physical (i.e., energetic) and/or psychosocial stress among high‐status individuals. Within‐group distributions of stress have direct developmental plasticity and life history implications relating to lifelong variation in phenotype and fitness (see above).

Comparative data from NHPs indicates that, in contrast to findings for humans, greater social dominance is related to greater cortisol concentrations. This finding is robust and includes studies assessing urinary and fecal cortisol levels among chimpanzees (Emery Thompson et al., 2020; Muller & Wrangham, 2004b; Preis et al., 2019), bonobos (Surbeck et al., 2012; Wobber et al., 2010), capuchins (Schoof et al., 2012; Schrock et al., 2019), baboons (Weingrill et al., 2004), macaques (Barrett et al., 2002; Higham et al., 2013; Qin et al., 2013; Vandeleest et al., 2020; Zhang et al., 2018), and platyrrhines (Bales et al., 2006). The observation of positive relationships between cortisol and dominance rank aligns with that for C‐peptide among NHPs and points to the importance of physical/energetic challenges experienced by high‐ranking individuals. The nature of the difference in the social status/dominance‐cortisol relationship between humans and NHPs remains unclear, but it is likely driven by differences in the relative strictness of social hierarchies, rank stability, and environmental resource abundance (Sapolsky, 2005). Regardless, these MIB findings indicate something unique about the evolution of human social dynamics and social structure.

#### 2.3.3 Testosterone (T)

The use of T as a MIB has also led to novel evolutionary insights into the links between social status/dominance and male behavioral energetics and life history. Among its many androgenic actions, T is known to regulate striving and aggressive dominance behaviors (Casto & Edwards, 2016; Kordsmeyer et al., 2019). Researchers in HEB have capitalized on this role, and applications of T as a MIB within related evolutionary social behavioral contexts can now be found among humans (see review in Booth et al., 2006; Muehlenbein & Bribiescas, 2005) and a long list of comparative NHPs (Emery Thompson et al., 2012; Marty et al., 2015; Mendonça‐Furtado et al., 2014; Muehlenbein et al., 2004; Muehlenbein & Watts, 2010; Muller & Wrangham, 2004a; Ross & French, 2011; Schoof et al., 2014; Wobber et al., 2010).

Positive associations between status/dominance and male T concentrations have been observed using salivary samples among humans (Booth et al., 2006) as well urine samples among chimpanzees and gorillas (Muller & Wrangham, 2004a; Robbins & Czekala, 1997) and fecal samples among bearded capuchins (Mendonça‐Furtado et al., 2014). These findings support the view of primate T as a “competition hormone” that calibrates status‐enhancing motivations through aggressive and competitive behaviors (Mazur & Booth, 1997), ultimately improving reproductive success and fitness (Bribiescas, 2001). Notably, however, the positive relationship between social status/dominance and T has not been consistently identified across human and NHP studies (e.g., Kordsmeyer et al., 2019). Deviation from this pattern has typically been explained with the “Challenge Hypothesis” (Wingfield, 2017; Wingfield et al., 1990). The Challenge Hypothesis suggests that natural selection has often favored the evolution of a physiological system in which testosterone levels are flexible and increase only when most critical for fitness (e.g., during male–male competition to improve performance and survivability), thereby avoiding the many deleterious consequences of chronically high T levels (e.g., wasting, immunosuppression). Support for the Challenge Hypothesis has been provided using salivary measures of T among humans in small‐scale populations (Trumble et al., 2012) as well as using urinary and fecal measures of T among wild chimpanzees (Muller & Wrangham, 2004a) and several other NHPs (Cavigelli & Pereira, 2000; Girard‐Buttoz et al., 2015). Collectively, this research points to complex relationships linking T with dominance behaviors and the evolution of social dynamics and hierarchies among humans and their primate relatives. More work is needed to directly investigate the fitness implications of these relationships.

## 3 UNDER‐INVESTIGATED MIB APPLICATIONS IN HEB

### 3.1 MIBs of linear growth

Linear growth is often a focus of research in HEB. At the species and population levels, human and NHP patterns of growth are studied as windows into environmental conditions, life history evolution, and the selective forces that have shaped body size and the dynamics of ontogeny (Bogin, 1999; Cheverud et al., 1992; Kuo & Chen, 2017; Leigh, 2001; Tanner et al., 1990; Urlacher, Blackwell, et al., 2016). At the level of the individual, linear growth variation is often used to investigate developmental plasticity and evolved norms of reaction (Bogin et al., 2007; Hochberg, 2011; Urlacher, Liebert, et al., 2016). The now well‐established relationship between human growth and later life chronic disease risk (Dewey & Begum, 2011) also makes linear growth a focal point for understanding the developmental origins of health and disease within the HEB subfield of evolutionary medicine (Berger et al., 2021).

Despite a long‐standing focus on linear growth in HEB, the field has been slow to adopt new methods of growth assessment to complement traditional anthropometry. This has limited research opportunities. Anthropometric assessment of linear growth (e.g., in stature among humans and comparative crown‐rump length among NHPs) is characterized by a relatively high degree of measurement error, requires multi‐point data collection to document current growth – typically over a period of several months or longer – and is often prohibitively difficult or invasive to perform among NHPs (Mattison & Vaughan, 2017). A small number of HEB studies among both humans and NHPs have explored the possibility of measuring serum insulin‐like growth factor 1 (IGF‐1) as a biomarker for linear growth (see review in Bernstein, 2017). However, the invasiveness of this approach (currently requiring venipuncture) and the activity of IGF‐1 on multiple biological systems other than linear growth has limited its application.

Several MIBs of liner growth—increasingly common in other fields—offer promise for overcoming methodological limitations in HEB. These MIBs are objective, single‐point, and are being developed predominantly using urine and DBS samples that are familiar to researchers. Typically, they use commercially available ELISA or RIA kits. Anthropometry will remain a critical tool for assessing growth in HEB. However, MIBs of linear growth, if properly validated, offer alternative or complementary measures that may be useful in specific contexts. Here, we highlight three analytes that hold promise as MIBs of linear growth.

#### 3.1.1 C‐terminal telopeptide (CTx)

C‐terminal telopeptide is an established marker of bone resorption and turnover. It is a small peptide fragment that is cleaved from collagen type 1 and released into circulation in a manner proportional to collagen degradation by osteoclasts (Rosen et al., 2000). Due to high sensitivity and specificity to osteoclast activity, CTx in blood has been widely used clinically and in research as a measure of human bone resorption and turnover rate (see review in Herrmann & Seibel, 2008). It has also been used for this purpose among long‐tailed macaques (e.g., Stroup et al., 2001; Wu et al., 2021). The small size of CTx allows it to be cleared by the kidneys, and it can be reliably measured in urine using a commercial ELISA kit. The dynamics of CTx and the nuances of its measurement in urine are well documented. Measurement is typically done using first morning void urine samples in fasted state in order to control for effects of food intake (Holst et al., 2007) and time of day (Bjarnason et al., 2002). A large reference for CTx values in healthy Austrian children age 0–18 years old (Rauchenzauner et al., 2007) provides a means for calculating CTx percentiles and performing sensitive statistical analysis. Levels of CTx mirror linear growth rates throughout childhood and during the adolescent growth spurt (Matsukura et al., 2003; Rauchenzauner et al., 2007; Szulc et al., 2000), demonstrating the utility of CTx as a MIB of growth.

#### 3.1.2 Osteocalcin

Osteocalcin is an established marker of bone formation. A major non‐collagenous matrix protein synthesized primarily by osteoblasts, osteocalcin in its carboxylated form has high affinity for hydroxyapatite and directly binds bone. As such, circulating osteocalcin levels have been shown to be directly proportional to osteoblast activity and rate of bone formation (Booth et al., 2013; Zoch et al., 2016). The short half‐life of osteocalcin and its diurnal rhythm of production (Shetty, 2016) render it most indicative of short‐term bone activity. Clinically, osteocalcin levels in blood are often used to monitor bone formation in metabolic diseases such as osteoporosis (Chen et al., 2000; Kuo & Chen, 2017). Importantly, serum osteocalcin is also currently used as a biomarker of linear growth, often in the context of disorders such as growth hormone deficiency and growth faltering (Zoch et al., 2016) but also in relation to rapid bone deposition and growth during adolescence (Lee et al., 2000). These studies demonstrate the close positive relationship between circulating osteocalcin and current linear growth. As with CTx, a large reference for serum osteocalcin values is available from healthy Austrian children age 0–18 years old (Rauchenzauner et al., 2007). A recent validation has established that osteocalcin can also be reliably measured in DBS samples using a modified protocol with a commercially available ELISA kit (Eick et al., 2020), opening the door for the integration of osteocalcin as a MIB in HEB growth‐related research.

#### 3.1.3 Tartrate resistant acid phosphate (TRACP‐5b)

Tartrate resistant acid phosphate is another promising MIB of bone resorption and linear growth. Secreted by osteoclasts, TRACP‐5b is a small polypeptide chain. Its biological function is unknown, yet its circulating levels correlate closely with osteoclast number. As such, it is widely used as a blood marker of bone resorption and turnover (Halleen et al., 2006; Janckila & Yam, 2009). Its concentration in serum has been shown to correlate positively with children's linear growth (Lin et al., 2016). Similar to CTx and osteocalcin, a large reference for serum TRACP‐5b values is available from healthy Austrian children age 0–18 years old (Rauchenzauner et al., 2007). One measurement and potential biomarker advantage over CTx and osteocalcin is that TRACP‐5b is more stable in circulation, with minimal diurnal variability or response to feeding (Janckila & Yam, 2009). As for osteocalcin, a recent validation has established that TRACP‐5b can be reliably measured in DBS samples using a modified protocol with a commercially available ELISA kit (Eick et al., 2019). Collectively, this work supports exploring TRACP‐5b for application as a MIB in HEB growth‐related research.

### 3.2 MIBs of gut function

Gut function has been an important topic of study in HEB for several decades. The Expensive Tissue Hypothesis (Aiello & Wheeler, 1995), for example, was formative in proposing an energetic link between gut size and encephalization in the evolution of primates. More recently, researchers in HEB have embraced the study of the gut microbiome to investigate variation in human and comparative NHP metabolism, life history, and health (Amato et al., 2016; Bennett et al., 2016; Clayton et al., 2018; Tung et al., 2015). Despite this sustained research focus on the gut, little remains known about variation in some of the most basic aspects of human and NHP gut function, including gut absorptive capacity and gut permeability. Gut absorptive capacity (i.e., ability to extract nutrients from ingested food) has direct implications for foraging efficiency and, ultimately, energy availability. Gut permeability (i.e., integrity of the gut barrier) has implications for host‐pathogen interactions and, ultimately, energy expenditure via ability to prevent ingested pathogens from entering circulation and triggering costly immune responses.

Evidence from fields outside of HEB suggests that variation in gut absorptive capacity and permeability are substantial among humans (Damms‐Machado et al., 2017; Dutta et al., 2019; Li & Atkinson, 2015) as well as NHPs (Bethune et al., 2008; Garg et al., 2015; Zhang et al., 2020). One primary source of variation globally among humans appears to be environmental enteric dysfunction (EED)—an acquired condition characterized by microbial translocation across the small intestine and reduced absorptive capacity—that is common in contexts of high pathogen exposure (Kosek, 2017; Marie et al., 2018; Mbuya & Humphrey, 2016; Prendergast et al., 2014).

Documenting human and NHP variation in gut absorptive capacity and permeability and investigating the ecological drivers of such variation is a critical step toward understanding the evolution of digestion, metabolism, and life history as well as the mechanisms regulating health and disease. The use of MIBs is a promising approach for accomplishing this task. Several MIBs of gut absorption and permeability have already been developed in the sciences. Here, we highlight three of the most promising MIBs for application in HEB.

#### 3.2.1 Claudins

Claudins are a family of proteins that are established biomarkers of gut absorptive capacity, a position relating to their role as key regulators of tight junction ion selectivity in the intestinal epithelial barrier (Guerrant et al., 2016b; Harper et al., 2018). Two claudins to receive particular attention by researchers are Claudin‐15 and Claudin‐2. These are used clinically in detecting intestinal damage and early stages of development of diseases such as necrotizing enterocolitis (Blackwood et al., 2017) and inflammatory bowel disease (Thuijls et al., 2010). Circulating levels of Claudin‐15 in humans have been shown to closely reflect intestinal barrier absorptive function as determined using gold‐standard jejunal biopsy (Guerrant et al., 2016b; Günzel & Yu, 2013). Recently, urine concentrations of both Claudin‐15 and Claudin‐2—measured using commercial ELISA—have similarly been shown to exhibit positive relationships with children's gut absorptive capacity assessed using dual sugar test (Guerrant et al., 2016b) and jejunal biopsy (Blackwood et al., 2017), respectively. This initial validation work is promising. However, additional research is needed to determine the usefulness of urinary claudins as MIBs of gut absorptive capacity in HEB.

#### 3.2.2 Zonulin

Zonulin is a promising biomarker of gut permeability. A haptoglobin protein that induces the breakdown of epithelial tight junctions, zonulin acts to reversibly increase intestinal permeability (Fasano, 2011; Fasano, 2012). Clinically, it has been indicated in the pathogenesis of several chronic inflammatory conditions, including celiac disease and type 1 diabetes (Ciccia et al., 2017; Fasano, 2011; Fasano, 2020; Hendy et al., 2017; Küme et al., 2017). Further supporting its role as a biomarker of gut permeability, circulating zonulin is consistently elevated among children suffering from or presumed to be suffering from EED (Guerrant et al., 2016a; Tickell et al., 2019; Zambruni et al., 2019). A commercial ELISA kit has been developed for measuring zonulin in fecal samples, and fecal zonulin has also been shown to correlate well with dual sugar test measures of gut permeability (Damms‐Machado et al., 2017; Seethaler et al., 2021). Zonulin is also a promising candidate for measurement in DBS, but additional validation work is needed.

#### 3.2.3 Endotoxin‐core antibodies (EndoCAb)

Endotoxin‐core antibodies (EndoCAb) are an additional promising biomarker of gut permeability. The production of EndoCAb and its concentration in circulation increases as a degree of intestinal permeability and associated translocation of bacterial products across the epithelial barrier into the bloodstream (Brenchley & Douek, 2012). Given this strong, albeit indirect, relationship with gut permeability, EndoCAb measured by venipuncture blood sampling has been successfully used as a biomarker of gut permeability in numerous studies examining EED and other intestinal conditions among humans (Campbell et al., 2003; Mondal et al., 2012; Mwape et al., 2017; Uddin et al., 2021). Importantly, EndoCAb measurement using a commercial ELISA kit has been preliminarily validated for minimally invasive DBS, with a strong reported correlation (*r* = 0.965) between matched DBS and plasma values (Hoke et al., 2018). This study, although limited by a small sample size (*N* = 18), provides initial support for the application of EndoCAb as a MIB of gut permeability in HEB.

### 3.3 Epigenetic MIBs of developmental plasticity

Epigenetic research continues to gain interest in HEB. Epigenetic modifications are heritable changes in gene expression which occur via biochemical mechanisms (i.e., DNA methylation, histone modification, noncoding RNA's) that do not alter the gene sequence itself but that can have large impacts on phenotype (Mulligan, 2016; Non & Thayer, 2019). Researchers in HEB have taken interest in epigenetic modifications due to characteristics such as sensitivity to environmental experiences (i.e., nutritional intake, psychosocial stress, toxicant exposure, racial disparities) (Kuzawa & Sweet, 2009; Thayer & Kuzawa, 2011) and the ability to influence phenotypic variation across the life course. At the individual, population, and species levels, HEB epigenetics research is being used to investigate evolutionary questions on life history, developmental plasticity, and the developmental origins of health and disease (Lea et al., 2017; Mulligan, 2016; Thayer & Kuzawa, 2011).

Problematically, existing epigenetics research in HEB among contemporary human populations has largely been performed using invasive venipuncture blood samples. Recent validations of minimally invasive sample types provide opportunity to expand this work using epigenetic MIBs. Here, we highlight two common epigenetic markers that offer promise for application as MIBs of developmental plasticity in HEB.

#### 3.3.1 DNA methylation patterns

DNA methylation is a well characterized epigenetic mechanism that involves covalent binding of a methyl group to DNA on cytosines, primarily in CpG sites, inhibiting binding of transcription factors otherwise needed for gene expression (Langie et al., 2017). It is a primary epigenetic pathway altering gene regulation in mammals, and patterns of methylation can help explain basic developmental processes as well as lifetime phenotypic responses to environmental stimuli (Burdge & Lillycrop, 2010). Patterns of methylation can be identified globally or in a more targeted manner using PCR and other technologies with small quantities of DNA (Singer, 2019). Research in HEB using invasive venipuncture blood sampling has demonstrated the utility of DNA methylation assessment for addressing developmental plasticity and evolutionary questions, including the adaptive nature of the impact of human early life social environments (McDade et al., 2017) and resource availability (McDade et al., 2019) on adult inflammatory immune processes. Comparative work among NHPs has provided additional evidence linking early environmental conditions to adaptive plasticity in several adult behavioral/physiological phenotypic traits (Anderson et al., 2021; Lea et al., 2016; Tung et al., 2012). This work is promising. However, recent advancements in the ability to assess methylation patterns in minimally invasive sample types such as feces (Belshaw et al., 2004), urine (Jatkoe et al., 2015), DBS (Hollegaard et al., 2013), saliva (Matthews et al., 2013), and buccal cells (Claire Mulot et al., 2004) provides opportunity to much more broadly and systematically utilize MIB approaches of DNA methylation in HEB.

#### 3.3.2 Telomere length

Telomere length is epigenetically regulated via DNA methylation and represents another promising MIB of developmental plasticity and variation in biological aging. Structurally, telomeres are repetitive DNA sequences that comprise the cap‐like protein structures found at the ends of chromosomes (Blackburn, 1991; Eisenberg, 2011). The length of telomeres is reduced over the lifespan with successive DNA replication, increasing opportunity for damage to the genome and accelerating biological senescence (Eisenberg, 2011). However, telomeres are susceptible to DNA methylation (Levis et al., 1985) and evidence suggests that this modification functions to reduce telomere shortening throughout life (García‐Cao et al., 2002; García‐Cao et al., 2004; Gonzalo et al., 2005; Gonzalo et al., 2006). Telomere length is thus used as an epigenetic marker of biological aging. Evolutionarily, telomere length may serve as a biomarker of variation in energy allocated to cellular maintenance (e.g., anti‐aging) activity across the life course (Eisenberg, 2011). Recent work has validated the measurement of telomere length in minimally invasive saliva, DBS, and buccal cell samples (Goldman et al., 2018; Rej et al., 2021; Stout et al., 2017), and researchers in HEB have preliminarily started to adopt the use of telomere length as an MIB. This initial research has led to the identification of negative effects of psychosocial stress (Rej et al., 2020) and adverse childhood experiences (Puterman et al., 2016) on human telomere length, suggesting more rapid biological aging with early life experienced adversity. This work supports the proposed adaptive nature of variation in telomere length and warrants additional MIB work in HEB.

## 4 FUTURE DIRECTIONS FOR MIB RESEARCH IN HEB

**FIGURE 1:**
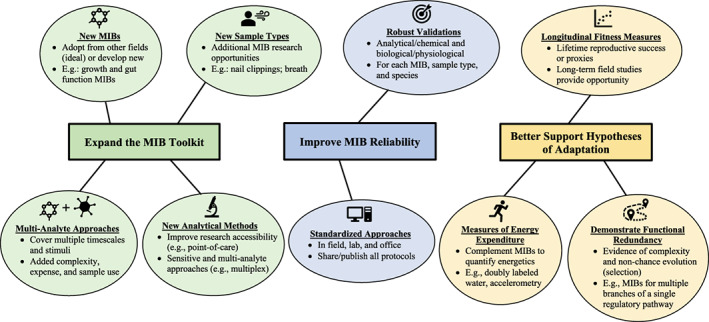
Future directions in minimally invasive biomarker (MIB) research in HEB (overview)

Despite decades of progress and many successes, limitations remain in HEB research using MIBs. Many of these limitations have been noted above for specific situations but require more general discussion. Here, we point to some of the most promising future directions for advancing MIB research in HEB (Figure 1).

### 4.1 Expand the MIB toolkit

#### 4.1.1 New MIBs

There will always be opportunity to develop new MIBs to expand the MIB toolkit and HEB's range of investigation. Many promising MIBs are already validated and in use in other fields, awaiting application to address evolutionary questions (see examples for growth, etc., above). These MIBs often require relatively little or no additional validation work and are particularly attractive for new research directions. In other cases, biomarkers already established with invasive sample types warrant development and validation as MIBs. Prospective MIBs must be evaluated carefully, with consideration of many potential factors limiting their application in new sample types (see validation discussion below; Lindau & McDade, 2008).

#### 4.1.2 Multi‐analyte approaches

Approaches that use multiple MIBs in combination hold promise in HEB, most obviously by facilitating investigation of novel relationships between two or more traits but also by providing information across different timescales and by improving ability to disentangle traits with multiple environmental inputs (e.g., the source of “stress”). The current use of multi‐analyte approaches is more common in HEB research among humans than NHPs. However, several recent examples demonstrate the value of multi‐MIB approaches for investigating comparative NHP topics ranging from developmental plasticity of the stress response (salivary cortisol and alpha‐amylase: Petrullo et al., 2016) to the behavioral and environmental sources of energy imbalance (urinary C‐peptide and fecal T3: Rangel‐Negrin et al., 2021). The ability to utilize multiple MIBs is becoming more practical with the development of multi‐analyte laboratory techniques that minimize sample waste (see below).

#### 4.1.3 New minimally invasive sample types

Researchers in HEB are well positioned to be leaders in expanding the range of minimally invasive sample types used in MIB assessment. There are several promising biospecimen types to consider. Nail clippings, for example, are now established in several fields as viable for the measurement of hormones and other analytes (Jaramillo Ortiz et al., 2021), most notably cortisol (Fischer et al., 2020). Recent work in HEB has shown the promise of this approach, demonstrating that fingernail cortisol levels among forager children of the Congo Basin are inversely correlated with measures of experienced fathering quality (Gettler et al., 2021). Sample types such as skin (Wang & Maibach, 2011), breath (Das & Pal, 2020), and undried finger‐prick whole blood (Blackwell et al., 2021) offer additional promise for MIB development in specific situations and with specific analytes.

#### 4.1.4 New analytical measurement methods

Most MIB research in HEB continues to use single‐analyte immunoassays (ELISA and RIA) for biomarker laboratory measurement. These methods are established and relatively affordable and approachable (Grange et al., 2014). Increasingly, however, researchers are turning to alternative methods to provide greater analytical sensitivity or to measure multiple analytes at once. Bead‐based immunoassays (i.e., multiplex assays) and flow cytometry have been used to measure panels of immunological biomarkers among humans and NHPs using venous blood (Giavedoni, 2005) and, in more limited instances, using minimally invasive sample types (for a recent example with DBS, see McDade et al., 2021). Liquid chromatography mass spectrometry (LCMS)—validated for measuring a range of biomarkers in urine and feces (Murtagh et al., 2013)—has also been applied in HEB for sensitive MIB assessment (Vogel et al., 2012; Weltring et al., 2012).

The primary factors limiting wider use of multi‐analyte measurement approaches in HEB are the complexity and costs involved in purchasing, maintaining, and operating needed equipment. These challenges will not easily be overcome and disproportionately impact researchers based in the low‐ and middle‐income countries where much HEB research continues to be performed. A focus of the field should therefore be on utilizing analytical approaches that not only perform well but are accessible (e.g., widely available, low cost, easy to use). In this regard, point‐of‐care (POC) measurement technologies hold promise. Some POC technologies are already regularly used to measure MIBs in HEB research, including dipstick urine test strips to detect the presence of ketones and metabolic stress (e.g., Knott, 1998; Muller & Wrangham, 2005; Naumenko et al., 2020) and portable photometric and electrochemical devices that use finger‐prick blood to measure circulating hemoglobin, glucose, and blood lipids (e.g., DeLouize et al., 2022; Levy et al., 2018; Liebert et al., 2013; Raichlen et al., 2017). However, as discussed by Gildner et al. in their recent review of POC technology (Gildner et al., 2021), barriers to POC application remain, including a general lack of device validations performed under typical HEB field conditions.

### 4.2 Improve MIB reliability

#### 4.2.1 Robust validations for each MIB (for each sample type, for each species)

Many reviews have been published on the methodological issues associated with MIB research and the importance of proper validations for ensuring reliability (Buchanan & Goldsmith, 2004; Saxbe, 2008; Touma & Palme, 2005; Trost & Mulhall, 2016), including reviews specific to HEB work with humans and NHPs (e.g., Anestis, 2010; Behringer & Deschner, 2017; Gray et al., 2017; Higham, 2016; Mcdade, 2013). In general, proper validations should be performed at both the analytical/chemical level—Is the measurement of the analyte accurate and reliable?—and the biological/physiological level—Does the analyte accurately and reliably reflect the trait of interest?—for each MIB, for each sample type, for each species.

Robust MIB validations are not currently the standard in HEB, although there many excellent examples (e.g., Behringer et al., 2017a; Eick et al., 2016; Emery Thompson & Knott, 2008; Higham et al., 2020; McDade et al., 2004). High quality analytical/chemical validations must report a range of MIB measurement characteristics, including sensitivity, specificity, linearity/parallelism, and biomarker stability. Biological/physiological validations should also be performed with each new sample type proposed to demonstrate that a MIB does indeed reflect the trait of interest. Similarly, the application of MIBs to new comparative primate species—possessing potential differences in biomarker structures and sensitivities—must be justified. Additional underexplored issues to consider in MIB validations include variation in the relationship between biomarker production and clearance rates and the biological significance of carrier proteins and metabolite conjugation that may moderate biomarker activity and relationships with phenotype.

#### 4.2.2 Standardized approaches in the field, lab, and office

There is likely no more effective or efficient way to advance the use of MIBs in HEB than to adopt standardized approaches for collecting, analyzing, and reporting data (Mulligan et al., in press; Poisot et al., 2019). Research groups should freely share and, ideally, publish detailed protocols for data collection and analysis. This information should include the specifics of sample collection, any data collection tools, lab protocols and reference/control information, and code and programs developed for data analysis. This standardization would lead to higher quality research and would improve reliability when comparing MIB findings across studies, research sites, and labs.

### 4.3 Better support hypotheses of adaptation

#### 4.3.1 Complementary measures of energy expenditure

The role of energetics in many HEB studies using MIBs has been established. Energetics are central to MIB research addressing life history questions. Hormones like cortisol, T, and T3, for example, appear to have evolved as physiological messengers to coordinate adaptive patterns of energy expenditure. Including these MIBs in research designs can therefore support the existence of energetic trade‐offs between competing life tasks by targeting the pathways of energy regulation that underlie observed correlations of traits (Bribiescas & Ellison, 2008). While such analyses are powerful, this approach is limited by not actually measuring energy expenditure or accounting for potential differences in daily energy budgets. Recent research demonstrates the usefulness of study designs that pair MIBs with complementary measures of energy expenditure to investigate adaptive patterns of energy use. Research among hunter‐gatherers in Tanzania, for example, has combined urine measures of oxidative stress with urine doubly labeled water (DLW) measures of daily total energy expenditure (TEE, kcal/day) to support the existence of fitness‐enhancing trade‐offs between somatic aging defense and other life tasks (Pontzer et al., 2015). Research among forager‐horticulturalists of Ecuador has similarly combined MIBs of immune activity with DLW measures of TEE and respirometry‐based (breath) measures of basal metabolism to provide “calorie‐level” evidence for adaptive energetic trade‐offs between immune activity and childhood growth (Urlacher et al., 2019a; Urlacher et al., 2021a). Studies such as these highlight the promise of combining MIBs and measures of energy expenditure to better support adaptive energetic hypotheses.

#### 4.3.2 Evidence of functional redundancy

It is well established in evolutionary biology that the regulation of variation in a phenotypic trait (i.e., the regulation of plasticity) is itself a trait that can be selected and adaptive (West‐Eberhard, 2003a). Recognizing this, a growing target of HEB research is to demonstrate the fitness‐enhancing functions of regulatory physiological pathways, for example those involved in energetic trade‐offs (Bribiescas & Ellison, 2008; Flatt & Heyland, 2011; Ricklefs & Wikelski, 2002; Zera & Harshman, 2001). This work is promising, but it often struggles to demonstrate that the regulation of a trait or set of traits is indeed adaptive and not the result of pathology (i.e., breakdown of physiological function) or chance evolution (e.g., random selection, genetic drift). Providing evidence of functional redundancy in regulatory pathways may be helpful in such cases. Functional redundancy supports the adaptive nature of a trait by providing evidence for a specific evolved function and evolved complexity (Williams, 2008). Demonstrating that multiple branches of a single regulatory pathway serve the exact same redundant function—for example the inhibition of child growth by not one but numerous direct and indirect actions of inflammatory immune activity (Cirillo et al., 2017)—increases confidence in identifying regulation that is adaptive and evolved via natural selection. More research in HEB should aim to demonstrate functional redundancy, for example using multi‐MIB approaches, to support adaptive hypotheses.

#### 4.3.3 Longitudinal measures of fitness

As noted by others (Beehner & Bergman, 2017), research in HEB has disproportionately focused on identifying the sources of variation in MIBs rather than on their presumed relationships with fitness. Troublingly few studies in HEB have collected the longitudinal data necessary to reliably document fitness effects for presumed adaptive traits. Identifying lifetime reproductive success should be the goal of this work. Yet, even longitudinally assessed proxies for fitness (e.g., inter‐birth interval, offspring survival) are often lacking in evolutionary hypothesis testing. Measuring lifetime fitness outcomes among humans and other primates with slow life histories is inherently difficult owing to the challenges of sustaining long‐term research infrastructure and funding. However, a growing number of successful HEB field studies utilizing MIBs offer examples for approaches for compiling the robust longitudinal datasets that will guide future advancements in the field by demonstrating lifetime fitness effects.

## 5 CONCLUSIONS

Research in HEB has a rich history using MIBs to document variation in key aspects of human and comparative NHP phenotype. A wide range of MIBs are also being productively applied to operationalize and test evolutionary hypotheses, including those relating to topics such as life history, developmental plasticity, and social dynamics. This work is expansive and was only cursorily covered in this paper. Given the breadth/depth of its current MIB research activity and the nature of its research foundation in both the field and in the lab, HEB is well‐positioned to remain a leader in MIB development and application. We have outlined several promising avenues for advancing the field. Apart from continuing to expand the MIB toolkit, researchers should improve trust in MIB reliability and better support hypotheses of adaptation. The use of MIBs is likely to remain a hallmark of HEB for many years to come, and continued progress in this area holds promise for advancing our understanding of what is means to be human.

## AUTHOR CONTRIBUTIONS

Samuel S. Urlacher: conceptualization (lead), writing – original draft (lead), writing – review and editing (lead). Elizabeth Y. Kim: conceptualization (equal), writing – original draft (equal), writing – review and editing (equal). Tiffany Luan: conceptualization (equal), writing – original draft (equal), writing – review and editing (supporting). Lauren J. Young: conceptualization (supporting), writing – original draft (supporting), writing – review and editing (supporting). Brian Adjetey: writing – original draft (supporting), writing – review and editing (supporting).

## CONFLICT OF INTEREST

The authors declare no conflicts of interest.

## Supporting information


**Table S1** Supporting Information.

## Data Availability

Data sharing is not applicable to this article as no new data were created or analyzed in this study.

## References

[ajhb23811-bib-0001] Adam, E. K. , & Kumari, M. (2009). Assessing salivary cortisol in large‐scale, epidemiological research. Psychoneuroendocrinology, 34(10), 1423–1436. 19647372 10.1016/j.psyneuen.2009.06.011

[ajhb23811-bib-0002] Aiello, L. C. , & Wheeler, P. (1995). The expensive‐tissue hypothesis: The brain and the digestive system in human and primate evolution. Current Anthropology, 36(2), 199–221.

[ajhb23811-bib-0003] Alberts, S. C. , Altmann, J. , & Wilson, M. L. (1996). Mate guarding constrains foraging activity of male baboons. Animal Behaviour, 51(6), 1269–1277.

[ajhb23811-bib-0004] Altmann, J. , Gesquiere, L. , Galbany, J. , Onyango, P. O. , & Alberts, S. C. (2010). Life history context of reproductive aging in a wild primate model. Annals of the New York Academy of Sciences, 1204(1), 127–138. 20738283 10.1111/j.1749-6632.2010.05531.x PMC3399114

[ajhb23811-bib-0005] Alvarado, L. C. , Muller, M. N. , Emery Thompson, M. , Klimek, M. , Nenko, I. , & Jasienska, G. (2015). The paternal provisioning hypothesis: Effects of workload and testosterone production on men's musculature. American Journal of Physical Anthropology, 158(1), 19–35. 26123405 10.1002/ajpa.22771

[ajhb23811-bib-0006] Alvarado, L. C. , Valeggia, C. R. , Ellison, P. T. , Lewarch, C. L. , & Muller, M. N. (2019). A comparison of men's life history, aging, and testosterone levels among Datoga pastoralists, Hadza foragers, and Qom transitional foragers. Adaptive Human Behavior and Physiology, 5(3), 251–273.

[ajhb23811-bib-0007] Alvergne, A. , Faurie, C. , & Raymond, M. (2008). Developmental plasticity of human reproductive development: Effects of early family environment in modern‐day France. Physiology & Behavior, 95(5), 625–632. 18822309 10.1016/j.physbeh.2008.09.005

[ajhb23811-bib-0008] Alvergne, A. , Faurie, C. , & Raymond, M. (2009). Variation in testosterone levels and male reproductive effort: Insight from a polygynous human population. Hormones and Behavior, 56(5), 491–497. 19664637 10.1016/j.yhbeh.2009.07.013

[ajhb23811-bib-0009] Amato, K. R. , Martinez‐Mota, R. , Righini, N. , Raguet‐Schofield, M. , Corcione, F. P. , Marini, E. , Humphrey, G. , Gogul, G. , Gaffney, J. , & Lovelace, E. (2016). Phylogenetic and ecological factors impact the gut microbiota of two neotropical primate species. Oecologia, 180(3), 717–733. 26597549 10.1007/s00442-015-3507-z

[ajhb23811-bib-0010] Anderson, D. P. , Nordheim, E. V. , Boesch, C. , & Moermond, T. (2002). Factors influencing fission‐fusion grouping in chimpanzees in the Taï National Park, Côte D'ivoire. In Behavioural diversity in chimpanzees and bonobos. Cambridge University Press. (pp. 90–101).

[ajhb23811-bib-0011] Anderson, J. A. , Johnston, R. A. , Lea, A. J. , Campos, F. A. , Voyles, T. N. , Akinyi, M. Y. , Alberts, S. C. , Archie, E. A. , & Tung, J. (2021). High social status males experience accelerated epigenetic aging in wild baboons. eLife, 10, e66128. 33821798 10.7554/eLife.66128 PMC8087445

[ajhb23811-bib-0012] Anestis, S. F. (2010). Hormones and social behavior in primates. Evolutionary Anthropology: Issues, News, and Reviews: Issues, News, and Reviews, 19(2), 66–78.

[ajhb23811-bib-0013] Anestis, S. F. , & Bribiescas, R. G. (2004). Rapid changes in chimpanzee (*Pan troglodytes*) urinary cortisol excretion. Hormones and Behavior, 45(3), 209–213. 15047016 10.1016/j.yhbeh.2003.09.015

[ajhb23811-bib-0014] Bahr, N. , Palme, R. , Möhle, U. , Hodges, J. , & Heistermann, M. (2000). Comparative aspects of the metabolism and excretion of cortisol in three individual nonhuman primates. General and Comparative Endocrinology, 117(3), 427–438. 10764553 10.1006/gcen.1999.7431

[ajhb23811-bib-0015] Bales, K. L. , French, J. A. , McWilliams, J. , Lake, R. A. , & Dietz, J. M. (2006). Effects of social status, age, and season on androgen and cortisol levels in wild male golden lion tamarins (*Leontopithecus rosalia*). Hormones and Behavior, 49(1), 88–95. 15978593 10.1016/j.yhbeh.2005.05.006

[ajhb23811-bib-0016] Bardi, M. , & Huffman, M. A. (2006). Maternal behavior and maternal stress are associated with infant behavioral development in macaques. Developmental Psychobiology, 48(1), 1–9. 16381034 10.1002/dev.20111

[ajhb23811-bib-0017] Barrett, G. M. , Shimizu, K. , Bardi, M. , Asaba, S. , & Mori, A. (2002). Endocrine correlates of rank, reproduction, and female‐directed aggression in male Japanese macaques (*Macaca fuscata*). Hormones and Behavior, 42(1), 85–96. 12191651 10.1006/hbeh.2002.1804

[ajhb23811-bib-0018] Beall, C. M. , Worthman, C. M. , Stallings, J. , Strohl, K. P. , Brittenham, G. , & Barragan, M. (1992). Salivary testosterone concentration of Aymara men native to 3600 m. Annals of Human Biology, 19(1), 67–78. 1734824 10.1080/03014469200001932

[ajhb23811-bib-0019] Beehner, J. C. , & Bergman, T. J. (2017). The next step for stress research in primates: To identify relationships between glucocorticoid secretion and fitness. Hormones and Behavior, 91, 68–83. 28284709 10.1016/j.yhbeh.2017.03.003

[ajhb23811-bib-0020] Behringer, V. , Deimel, C. , Hohmann, G. , Negrey, J. , Schaebs, F. S. , & Deschner, T. (2018). Applications for non‐invasive thyroid hormone measurements in mammalian ecology, growth, and maintenance. Hormones and Behavior, 105, 66–85. 30063897 10.1016/j.yhbeh.2018.07.011

[ajhb23811-bib-0021] Behringer, V. , & Deschner, T. (2017). Non‐invasive monitoring of physiological markers in primates. Hormones and Behavior, 91, 3–18. 28202354 10.1016/j.yhbeh.2017.02.001

[ajhb23811-bib-0022] Behringer, V. , Deschner, T. , Deimel, C. , Stevens, J. M. G. , & Hohmann, G. (2014). Age‐related changes in urinary testosterone levels suggest differences in puberty onset and divergent life history strategies in bonobos and chimpanzees. Hormones and Behavior, 66(3), 525–533. 25086337 10.1016/j.yhbeh.2014.07.011

[ajhb23811-bib-0023] Behringer, V. , Hohmann, G. , Stevens, J. M. , Weltring, A. , & Deschner, T. (2012). Adrenarche in bonobos (*Pan paniscus*): Evidence from ontogenetic changes in urinary dehydroepiandrosterone‐sulfate levels. Journal of Endocrinology, 214(1), 55–65. 22562655 10.1530/JOE-12-0103

[ajhb23811-bib-0024] Behringer, V. , Stevens, J. M. , Leendertz, F. H. , Hohmann, G. , & Deschner, T. (2017a). Validation of a method for the assessment of urinary neopterin levels to monitor health status in non‐human‐primate species. Frontiers in Physiology, 8, 51. 28220080 10.3389/fphys.2017.00051 PMC5292569

[ajhb23811-bib-0025] Behringer, V. , Stevens, J. M. G. , Leendertz, F. H. , Hohmann, G. , & Deschner, T. (2017b). Validation of a method for the assessment of urinary neopterin levels to monitor health status in Non‐human‐primate species. Frontiers in Physiology, 8, 1–11. 28220080 10.3389/fphys.2017.00051 PMC5292569

[ajhb23811-bib-0026] Belshaw, N. J. , Elliott, G. O. , Williams, E. A. , Bradburn, D. M. , Mills, S. J. , Mathers, J. C. , & Johnson, I. T. (2004). Use of DNA from human stools to detect aberrant CpG Island methylation of genes implicated in colorectal cancer. Cancer Epidemiology, Biomarkers & Prevention, 13(9), 1495–1501. 15342451

[ajhb23811-bib-0027] Bennett, G. , Malone, M. , Sauther, M. L. , Cuozzo, F. P. , White, B. , Nelson, K. E. , Stumpf, R. M. , Knight, R. , Leigh, S. R. , & Amato, K. R. (2016). Host age, social group, and habitat type influence the gut microbiota of wild ring‐tailed lemurs (*Lemur catta*). American Journal of Primatology, 78(8), 883–892. 27177345 10.1002/ajp.22555

[ajhb23811-bib-0028] Bentley, G. R. , Harrigan, A. M. , Campbell, B. , & Ellison, P. T. (1993). Seasonal effects on salivary testosterone levels among lese males of the Ituri forest. Zaire. American Journal of Human Biology, 5(6), 711–717. 28548365 10.1002/ajhb.1310050614

[ajhb23811-bib-0029] Berger, J. , Cohen, B. P. , & Zelditch, M. (1972). Status characteristics and social interaction. American Sociological Review, 37(3), 241.

[ajhb23811-bib-0030] Berger, J. , Rosenholtz, S. J. , & Morris, Z. J. (1980). Status organizing processes. Annual Review of Sociology, 6(1), 479–508.

[ajhb23811-bib-0031] Berger, S. M. , Griffin, J. S. , & Dent, S. C. (2021). Phenotypes and pathways: Working toward an integrated skeletal biology in biological anthropology. American Journal of Human Biology, 33(2), e23450. 32511865 10.1002/ajhb.23450

[ajhb23811-bib-0032] Berghänel, A. , Heistermann, M. , Schülke, O. , & Ostner, J. (2016). Prenatal stress effects in a wild, long‐lived primate: Predictive adaptive responses in an unpredictable environment. Proceedings of the Royal Society B: Biological Sciences, 283(1839), 20161304. 10.1098/rspb.2016.1304 PMC5046897 27655764

[ajhb23811-bib-0033] Bernstein, R. M. (2017). Hormones and human and nonhuman primate growth. Hormone Research in Pædiatrics, 88(1), 15–21. 10.1159/000476065 28528334

[ajhb23811-bib-0034] Bernstein, R. M. , & Hinde, K. (2016). Bioactive factors in milk across lactation: Maternal effects and influence on infant growth in rhesus macaques (*Macaca mulatta*). American Journal of Primatology, 78(8), 838–850. 27029025 10.1002/ajp.22544 PMC5538777

[ajhb23811-bib-0035] Bethune, M. T. , Borda, J. T. , Ribka, E. , Liu, M.‐X. , Phillippi‐Falkenstein, K. , Jandacek, R. J. , Doxiadis, G. G. , Gray, G. M. , Khosla, C. , & Sestak, K. (2008). A non‐human primate model for gluten sensitivity. PLoS One, 3(2), e1614. 18286171 10.1371/journal.pone.0001614 PMC2229647

[ajhb23811-bib-0036] Bjarnason, N. , Henriksen, E. , Alexandersen, P. , Christgau, S. , Henriksen, D. , & Christiansen, C. (2002). Mechanism of circadian variation in bone resorption. Bone, 30(1), 307–313. 11792602 10.1016/s8756-3282(01)00662-7

[ajhb23811-bib-0037] Blackburn EH . 1991. Structure and function of telomeres. 350:5. 10.1038/350569a0 1708110

[ajhb23811-bib-0038] Blackwell, A. D. , Garcia, A. R. , Keivanfar, R. L. , & Bay, S. (2021). A field method for cryopreservation of whole blood from a finger prick for later analysis with flow cytometry. American Journal of Physical Anthropology, 174(4), 670–685. 33595836 10.1002/ajpa.24251

[ajhb23811-bib-0039] Blackwell, A. D. , Gurven, M. D. , Sugiyama, L. S. , Madimenos, F. C. , Liebert, M. A. , Martin, M. A. , Kaplan, H. S. , & Snodgrass, J. J. (2011). Evidence for a peak shift in a humoral response to helminths: Age profiles of IgE in the Shuar of Ecuador, the Tsimane of Bolivia, and the U.S. NHANES. PLoS Neglected Tropical Diseases, 5(6), e1218. 21738813 10.1371/journal.pntd.0001218 PMC3125146

[ajhb23811-bib-0040] Blackwell, A. D. , Snodgrass, J. J. , Madimenos, F. C. , & Sugiyama, L. S. (2010). Life history, immune function, and intestinal helminths: Trade‐offs among immunoglobulin E, C‐reactive protein, and growth in an Amazonian population. American Journal of Human Biology: The Official Journal of the Human Biology Council, 22(6), 836–848. 20865759 10.1002/ajhb.21092 PMC4512836

[ajhb23811-bib-0041] Blackwood, B. P. , Yuan, C. Y. , Wood, D. R. , Nicolas, J. D. , Grothaus, J. S. , & Hunter, C. J. (2017). Probiotic lactobacillus species strengthen intestinal barrier function and tight junction integrity in experimental necrotizing enterocolitis. Journal of Probiotics & Health, 5(1), 1000159. 10.4172/2329-8901.1000159 PMC5475283 28638850

[ajhb23811-bib-0042] Boas, F. (1912). Changes in the bodily form of descendants of immigrants. American Anthropologist, 14(3), 530–562.

[ajhb23811-bib-0043] Bogin, B. (1999). Evolutionary perspective on human growth. Annual Review of Anthropology, 28, 109–153. 10.1146/annurev.anthro.28.1.109 12295621

[ajhb23811-bib-0044] Bogin, B. , Silva, M. I. V. , & Rios, L. (2007). Life history trade‐offs in human growth: Adaptation or pathology? American Journal of Human Biology, 19(5), 631–642. 17636530 10.1002/ajhb.20666

[ajhb23811-bib-0045] Booth, A. , Granger, D. A. , Mazur, A. , & Kivlighan, K. T. (2006). Testosterone and social behavior. Social Forces, 85(1), 167–191.

[ajhb23811-bib-0046] Booth, S. L. , Centi, A. , Smith, S. R. , & Gundberg, C. (2013). The role of osteocalcin in human glucose metabolism: Marker or mediator? Nature Reviews Endocrinology, 9(1), 43–55. 10.1038/nrendo.2012.201 PMC4441272 23147574

[ajhb23811-bib-0047] Braude, S. , Tang‐Martinez, Z. , & Taylor, G. T. (1999). Stress, testosterone, and the immunoredistribution hypothesis. Behavioral Ecology, 10(3), 345–350.

[ajhb23811-bib-0048] Brenchley, J. M. , & Douek, D. C. (2012). Microbial translocation across the GI tract. Annual Review of Immunology, 30, 149–173. 10.1146/annurev-immunol-020711-075001 PMC3513328 22224779

[ajhb23811-bib-0049] Bribiescas, R. (2010). An evolutionary and life history perspective on human male reproductive senescence. Annals of the New York Academy of Sciences, 1204(1), 54–64. 20738275 10.1111/j.1749-6632.2010.05524.x

[ajhb23811-bib-0050] Bribiescas, R. , & Ellison, P. (2008). Chapter 7: How hormones mediate trade‐offs in human health and disease. In Evolution in Health and Disease (pp. 77–93). New York: Oxford University Press.

[ajhb23811-bib-0051] Bribiescas, R. G. (1996). Testosterone levels among Aché hunter‐gatherer men. Human Nature, 7(2), 163–188. 24203318 10.1007/BF02692109

[ajhb23811-bib-0052] Bribiescas, R. G. (2001). Reproductive ecology and life history of the human male. American Journal of Physical Anthropology: The Official Publication of the American Association of Physical Anthropologists, 116(S33), 148–176. 10.1002/ajpa.10025.abs 11786994

[ajhb23811-bib-0053] Bribiescas, R. G. (2020). Aging, life history, and human evolution. Annual Review of Anthropology, 49, 101–121.

[ajhb23811-bib-0054] Broche, N. , Takeshita, R. S. C. , Mouri, K. , Bercovitch, F. B. , & Huffman, M. A. (2019). Salivary alpha‐amylase enzyme is a non‐invasive biomarker of acute stress in Japanese macaques (*Macaca fuscata*). Primates, 60(6), 547–558. 31541328 10.1007/s10329-019-00757-6

[ajhb23811-bib-0055] Brockman, D. K. , Whitten, P. L. , Russell, E. , Richard, A. F. , & Izard, M. K. (1995). Application of fecal steroid techniques to the reproductive endocrinology of female Verreaux's sifaka (*Propithecus verreauxi*). American Journal of Primatology, 36(4), 313–325. 31924099 10.1002/ajp.1350360406

[ajhb23811-bib-0056] Buchanan, K. L. , & Goldsmith, A. R. (2004). Noninvasive endocrine data for behavioural studies: The importance of validation. Animal Behaviour, 67, 183–185.

[ajhb23811-bib-0500] Busch, D. S. , & Hayward, L. S. (2009). Stress in a conservation context: a discussion of glucocorticoid actions and how levels change with conservation‐relevant variables. Biological Conservation, 142.12, 2844–2853.

[ajhb23811-bib-0057] Burdge, G. C. , & Lillycrop, K. A. (2010). Nutrition, epigenetics, and developmental plasticity: Implications for understanding human disease. Annual Review of Nutrition, 30(1), 315–339. 10.1146/annurev.nutr.012809.104751 20415585

[ajhb23811-bib-0058] Burke, C. W. , & Shakespear, R. A. (1976). Triiodothyronine and thyroxine in urine. II. Renal handling, and effect of urinary protein. The Journal of Clinical Endocrinology & Metabolism, 42(3), 504–513. 1254691 10.1210/jcem-42-3-504

[ajhb23811-bib-0059] Burke, C. W. , Shakespear, R. A. , & Russell, F. T. (1972). Measurement of thyroxine and triiodothyronine in human urine. The Lancet, 300(7788), 1177–1179. 10.1016/s0140-6736(72)92597-4 4117596

[ajhb23811-bib-0060] Cadoux‐Hudson, T. , Few, J. , & Imms, F. (1985). The effect of exercise on the production and clearance of testosterone in well trained young men. European Journal of Applied Physiology and Occupational Physiology, 54(3), 321–325. 4065118 10.1007/BF00426153

[ajhb23811-bib-0061] Caldwell, A. E. (2016). Physical activity and life history theory. In Human physical fitness and activity (pp. 11–17). Springer.

[ajhb23811-bib-0062] Campbell, B. , & Mbizo, M. (2006). Reproductive maturation, somatic growth and testosterone among Zimbabwe boys. Annals of Human Biology, 33(1), 17–25. 16500808 10.1080/03014460500424068

[ajhb23811-bib-0063] Campbell, D. I. , Elia, M. , & Lunn, P. G. (2003). Growth faltering in rural Gambian infants is associated with impaired small intestinal barrier function, leading to endotoxemia and systemic inflammation. The Journal of Nutrition, 133(5), 1332–1338. 12730419 10.1093/jn/133.5.1332

[ajhb23811-bib-0064] Campbell, K. L. (1994). Blood, urine, saliva and dip‐sticks: Experiences in Africa, New Guinea, and Boston. Annals of the New York Academy of Sciences, 709(1), 312–330. 8154724 10.1111/j.1749-6632.1994.tb30419.x

[ajhb23811-bib-0065] Casto, K. V. , & Edwards, D. A. (2016). Testosterone, cortisol, and human competition. Hormones and Behavior, 82, 21–37. 27103058 10.1016/j.yhbeh.2016.04.004

[ajhb23811-bib-0066] Cavigelli, S. A. , & Pereira, M. E. (2000). Mating season aggression and fecal testosterone levels in male ring‐tailed lemurs (*Lemur catta*). Hormones and Behavior, 37(3), 246–255. 10868488 10.1006/hbeh.2000.1585

[ajhb23811-bib-0067] Cepon, T. J. , Snodgrass, J. J. , Leonard, W. R. , Tarskaia, L. A. , Klimova, T. M. , Fedorova, V. I. , Baltakhinova, M. E. , & Krivoshapkin, V. G. (2011). Circumpolar adaptation, social change, and the development of autoimmune thyroid disorders among the Yakut (Sakha) of Siberia. American Journal of Human Biology, 23(5), 703–709. 21732471 10.1002/ajhb.21200

[ajhb23811-bib-0068] Cepon‐Robins, T. J. , Blackwell, A. D. , Gildner, T. E. , Liebert, M. A. , Urlacher, S. S. , Madimenos, F. C. , Eick, G. N. , Snodgrass, J. J. , & Sugiyama, L. S. (2021). Pathogen disgust sensitivity protects against infection in a high pathogen environment. Proceedings of the National Academy of Sciences, 118(8), e2018552118. 10.1073/pnas.2018552118 PMC7923589 33597300

[ajhb23811-bib-0069] Cepon‐Robins, T. J. , Gildner, T. E. , Schrock, J. , Eick, G. , Bedbury, A. , Liebert, M. A. , Urlacher, S. S. , Madimenos, F. C. , Harrington, C. J. , & Amir, D. (2019). Soil‐transmitted helminth infection and intestinal inflammation among the Shuar of Amazonian Ecuador. American Journal of Physical Anthropology, 170(1), 65–74. 31260090 10.1002/ajpa.23897

[ajhb23811-bib-0070] Charmandari, E. , Tsigos, C. , & Chrousos, G. (2005). Endocrinology of the stress response. Annual Review of Physiology, 67, 259–284. 10.1146/annurev.physiol.67.040403.120816 15709959

[ajhb23811-bib-0071] Charnov, E. L. (1991). Evolution of life history variation among female mammals. Proceedings of the National Academy of Sciences, 88(4), 1134–1137. 10.1073/pnas.88.4.1134 PMC50971 1996315

[ajhb23811-bib-0072] Chen, H. , Yao, H. , Yang, W. , Xiang, Z. , Ostner, J. , & Cristόbal‐Azkarate, J. (2021). Validation of a fecal T3 metabolite assay for measuring energetics in wild golden snub‐nosed monkeys (*Rhinopithecus roxellana*). International Journal of Primatology, 42(5), 759–763.

[ajhb23811-bib-0073] Chen, Y. , Shimizu, M. , Sato, K. , Koto, M. , Tsunemi, K. , Yoshida, T. , & Yoshikawa, Y. (2000). Effects of aging on bone mineral content and bone biomarkers in female cynomolgus monkeys. Experimental Animals, 49(3), 163–170. 11109538 10.1538/expanim.49.163

[ajhb23811-bib-0074] Cheverud, J. M. , Wilson, P. , & Dittus, W. P. (1992). Primate population studies at Polonnaruwa. III. Somatometric growth in a natural population of toque macaques (Macaca sinica). Journal of Human Evolution, 23(1), 51–77.

[ajhb23811-bib-0075] Chisholm, J. S. , Ellison, P. T. , Evans, J. , Lee, P. C. , Lieberman, L. S. , Pavlik, Z. , Ryan, A. S. , Salter, E. M. , Stini, W. A. , & Worthman, C. M. (1993). Death, hope, and sex: Life‐history theory and the development of reproductive strategies. Current Anthropology, 34(1), 1–24.

[ajhb23811-bib-0076] Christiansen, K. H. (1991). Serum and saliva sex hormone levels in! Kung san men. American Journal of Physical Anthropology, 86(1), 37–44. 1951659 10.1002/ajpa.1330860103

[ajhb23811-bib-0077] Ciccia, F. , Guggino, G. , Rizzo, A. , Alessandro, R. , Luchetti, M. M. , Milling, S. , Saieva, L. , Cypers, H. , Stampone, T. , & Di Benedetto, P. (2017). Dysbiosis and zonulin upregulation alter gut epithelial and vascular barriers in patients with ankylosing spondylitis. Annals of the Rheumatic Diseases, 76(6), 1123–1132. 28069576 10.1136/annrheumdis-2016-210000 PMC6599509

[ajhb23811-bib-0078] Cirillo, F. , Lazzeroni, P. , Sartori, C. , & Street, M. E. (2017). Inflammatory diseases and growth: Effects on the GH–IGF axis and on growth plate. International Journal of Molecular Sciences, 18(9), 1878. 28858208 10.3390/ijms18091878 PMC5618527

[ajhb23811-bib-0079] Claire Mulot, I. S. , Clavel, J. , Beaune, P. , & Loriot, M.‐A. (2004). Collection of human genomic DNA from buccal cells for genetics studies: Comparison between cytobrush, mouthwash, and treated card. Journal of Biomedicine and Biotechnology, 2005(3), 291–296. 10.1155/JBB.2005.291 PMC1224694 16192688

[ajhb23811-bib-0080] Clancy, K. B. H. , Klein, L. D. , Ziomkiewicz, A. , Nenko, I. , Jasienska, G. , & Bribiescas, R. G. (2013). Relationships between biomarkers of inflammation, ovarian steroids, and age at menarche in a rural polish sample. American Journal of Human Biology, 25(3), 389–398. 23606228 10.1002/ajhb.22386

[ajhb23811-bib-0081] Clayton, J. B. , Gomez, A. , Amato, K. , Knights, D. , Travis, D. A. , Blekhman, R. , Knight, R. , Leigh, S. , Stumpf, R. , & Wolf, T. (2018). The gut microbiome of nonhuman primates: Lessons in ecology and evolution. American Journal of Primatology, 80(6), e22867. 29862519 10.1002/ajp.22867

[ajhb23811-bib-0082] Clow, A. , & Smyth, N. (2020). Salivary cortisol as a non‐invasive window on the brain. International Review of Neurobiology, 150, 1–16. 32204827 10.1016/bs.irn.2019.12.003

[ajhb23811-bib-0083] Cook, N. J. (2012). Minimally invasive sampling media and the measurement of corticosteroids as biomarkers of stress in animals. Canadian Journal of Animal Science, 92(3), 227–259.

[ajhb23811-bib-0084] Corley, M. , Perea‐Rodriguez, J. P. , Valeggia, C. , & Fernandez‐Duque, E. (2021). Associations between fecal cortisol and biparental care in a pair‐living primate. American Journal of Physical Anthropology, 176(2), 295–307. 34272723 10.1002/ajpa.24368 PMC8429222

[ajhb23811-bib-0085] Crimmins, E. M. , & Finch, C. E. (2006). Infection, inflammation, height, and longevity. Proceedings of the National Academy of Sciences, 103(2), 498–503. 10.1073/pnas.0501470103 PMC1326149 16387863

[ajhb23811-bib-0086] Cristóbal‐Azkarate, J. , Maréchal, L. , Semple, S. , Majolo, B. , & MacLarnon, A. (2016). Metabolic strategies in wild male barbary macaques: Evidence from faecal measurement of thyroid hormone. Biology Letters, 12(4), 20160168. 27095269 10.1098/rsbl.2016.0168 PMC4881361

[ajhb23811-bib-0087] Crockford, C. , Wittig, R. M. , Langergraber, K. , Ziegler, T. E. , Zuberbühler, K. , & Deschner, T. (2013). Urinary oxytocin and social bonding in related and unrelated wild chimpanzees. Proceedings of the Royal Society B: Biological Sciences, 280(1755), 20122765. 10.1098/rspb.2012.2765 PMC3574389 23345575

[ajhb23811-bib-0088] Damms‐Machado, A. , Louis, S. , Schnitzer, A. , Volynets, V. , Rings, A. , Basrai, M. , & Bischoff, S. C. (2017). Gut permeability is related to body weight, fatty liver disease, and insulin resistance in obese individuals undergoing weight reduction. The American Journal of Clinical Nutrition, 105(1), 127–135. 28049662 10.3945/ajcn.116.131110

[ajhb23811-bib-0089] Danforth, E. , & Burger, A. G. (1989). The impact of nutrition on thyroid hormone physiology and action. Annual Review of Nutrition, 9(1), 201–227. 10.1146/annurev.nu.09.070189.001221 2669870

[ajhb23811-bib-0090] Das, S. , & Pal, M. (2020). Non‐invasive monitoring of human health by exhaled breath analysis: A comprehensive review. Journal of the Electrochemical Society, 167(3), 037562.

[ajhb23811-bib-0091] De Dreu, C. K. W. , & Kret, M. E. (2016). Oxytocin conditions intergroup relations through upregulated in‐group empathy, cooperation, conformity, and defense. Biological Psychiatry, 79(3), 165–173. 25908497 10.1016/j.biopsych.2015.03.020

[ajhb23811-bib-0092] Decker, S. A. (2000). Salivary cortisol and social status among Dominican men. Hormones and Behavior, 38(1), 29–38. 10924284 10.1006/hbeh.2000.1597

[ajhb23811-bib-0093] DeLouize, A. M. , Liebert, M. A. , Madimenos, F. C. , Urlacher, S. S. , Schrock, J. M. , Cepon‐Robins, T. J. , Gildner, T. E. , Blackwell, A. D. , Harrington, C. J. , & Amir, D. (2022). Low prevalence of anemia among Shuar communities of Amazonian Ecuador. American Journal of Human Biology, 34(1), e23590. 33749068 10.1002/ajhb.23590

[ajhb23811-bib-0094] Desantis, A. S. , Kuzawa, C. W. , & Adam, E. K. (2015). Developmental origins of flatter cortisol rhythms: Socioeconomic status and adult cortisol activity. American Journal of Human Biology, 27(4), 458–467. 25753264 10.1002/ajhb.22668

[ajhb23811-bib-0095] Deschner, T. , Kratzsch, J. , & Hohmann, G. (2008). Urinary C‐peptide as a method for monitoring body mass changes in captive bonobos (*Pan paniscus*). Hormones and Behavior, 54(5), 620–626. 18638479 10.1016/j.yhbeh.2008.06.005

[ajhb23811-bib-0096] Desjardins, N. M. L. , Srivastava, S. , Küfner, A. C. P. , & Back, M. D. (2015). Who attains status? Similarities and differences across social contexts. Social Psychological and Personality Science, 6(6), 692–700.

[ajhb23811-bib-0097] Dettmer, A. M. , Murphy, A. M. , Guitarra, D. , Slonecker, E. , Suomi, S. J. , Rosenberg, K. L. , Novak, M. A. , Meyer, J. S. , & Hinde, K. (2018). Cortisol in neonatal mother's milk predicts later infant social and cognitive functioning in rhesus monkeys. Child Development, 89(2), 525–538. 28369689 10.1111/cdev.12783 PMC6528802

[ajhb23811-bib-0098] Dewey, K. G. , & Begum, K. (2011). Long‐term consequences of stunting in early life. Maternal & Child Nutrition, 7(3), 5–18. 21929633 10.1111/j.1740-8709.2011.00349.x PMC6860846

[ajhb23811-bib-0099] Dewsbury, D. A. (1982). Dominance rank, copulatory behavior, and differential reproduction. The Quarterly Review of Biology, 57(2), 135–159. 7051088 10.1086/412672

[ajhb23811-bib-0100] Dias, P. A. D. , Coyohua‐Fuentes, A. , Canales‐Espinosa, D. , Chavira‐Ramírez, R. , & Rangel‐Negrín, A. (2017). Hormonal correlates of energetic condition in mantled howler monkeys. Hormones and Behavior, 94, 13–20. 28602941 10.1016/j.yhbeh.2017.06.003

[ajhb23811-bib-0101] Dias, P. A. D. , Coyohua‐Fuentes, A. , Chavira‐Ramírez, D. R. , Canales‐Espinosa, D. , & Rangel‐Negrín, A. (2021). Correlates of hormonal modulation in mantled howler monkey males, *Alouatta palliata* . American Journal of Biological Anthropology, 178, 17–28. 10.1002/ajpa.24464 36787731

[ajhb23811-bib-0102] Dickerson, S. S. , & Kemeny, M. E. (2004). Acute stressors and cortisol responses: A theoretical integration and synthesis of laboratory research. Psychological Bulletin, 130(3), 355–391. 15122924 10.1037/0033-2909.130.3.355

[ajhb23811-bib-0103] Do Yup Lee, E. K. , & Choi, M. H. (2015). Technical and clinical aspects of cortisol as a biochemical marker of chronic stress. BMB Reports, 48(4), 209–216. 25560699 10.5483/BMBRep.2015.48.4.275 PMC4436856

[ajhb23811-bib-0104] Drews, C. (1993). The concept and definition of dominance in animal behaviour. Behaviour, 125(3–4), 283–313.

[ajhb23811-bib-0105] Dufour, D. L. , & Sauther, M. L. (2002). Comparative and evolutionary dimensions of the energetics of human pregnancy and lactation. American Journal of Human Biology, 14(5), 584–602. 12203813 10.1002/ajhb.10071

[ajhb23811-bib-0106] Dutta, D. , Methe, B. , Amar, S. , Morris, A. , & Lim, S. H. (2019). Intestinal injury and gut permeability in sickle cell disease. Journal of Translational Medicine, 17(1), 1–4. 31146745 10.1186/s12967-019-1938-8 PMC6543649

[ajhb23811-bib-0107] Eberle, M. , & Kappeler, P. M. (2004). Sex in the dark: Determinants and consequences of mixed male mating tactics in *Microcebus murinus*, a small solitary nocturnal primate. Behavioral Ecology and Sociobiology, 57(1), 77–90.

[ajhb23811-bib-0108] Eick, G. , Urlacher, S. S. , McDade, T. W. , Kowal, P. , & Snodgrass, J. J. (2016). Validation of an optimized ELISA for quantitative assessment of Epstein‐Barr virus antibodies from dried blood spots. Biodemography and Social Biology, 62(2), 222–233. 27337556 10.1080/19485565.2016.1169396 PMC4968568

[ajhb23811-bib-0109] Eick, G. N. , Devlin, M. J. , Cepon‐Robins, T. J. , Kowal, P. , Sugiyama, L. S. , & Snodgrass, J. J. (2019). A dried blood spot‐based method to measure levels of tartrate‐resistant acid phosphatase 5b (TRACP‐5b), a marker of bone resorption. American Journal of Human Biology, 31(3), e23240. 30897260 10.1002/ajhb.23240

[ajhb23811-bib-0110] Eick, G. N. , Madimenos, F. C. , Cepon‐Robins, T. J. , Devlin, M. J. , Kowal, P. , Sugiyama, L. S. , & Snodgrass, J. J. (2020). Validation of an enzyme‐linked immunoassay assay for osteocalcin, a marker of bone formation, in dried blood spots. American Journal of Human Biology, 32(5), e23394. 32017301 10.1002/ajhb.23394

[ajhb23811-bib-0111] Eisenberg, D. T. A. (2011). An evolutionary review of human telomere biology: The thrifty telomere hypothesis and notes on potential adaptive paternal effects. American Journal of Human Biology, 23(2), 149–167. 21319244 10.1002/ajhb.21127

[ajhb23811-bib-0112] Eisenberg, D. T. A. , Hayes, M. G. , & Kuzawa, C. W. (2012). Delayed paternal age of reproduction in humans is associated with longer telomeres across two generations of descendants. Proceedings of the National Academy of Sciences, 109(26), 10251–10256. 10.1073/pnas.1202092109 PMC3387085 22689985

[ajhb23811-bib-0113] Elia, M. (1992). Organ and tissue contribution to metabolic rate. In J. M. Kinney, & H. N. Tucker (Eds.), Energy Metabolism, Tissue Determinants and Cellular Corollaries (pp. 61–80). New York: Raven Press, Ltd.

[ajhb23811-bib-0114] Ellis, B. J. , & Del Giudice, M. (2019). Developmental adaptation to stress: An evolutionary perspective. Annual Review of Psychology, 70(1), 111–139. 10.1146/annurev-psych-122216-011732 30125133

[ajhb23811-bib-0115] Ellis, B. J. , Figueredo, A. J. , Brumbach, B. H. , & Schlomer, G. L. (2009). Fundamental dimensions of environmental risk: The impact of harsh versus unpredictable environments on the evolution and development of life history strategies. Human Nature, 20(2), 204–268. 25526958 10.1007/s12110-009-9063-7

[ajhb23811-bib-0116] Ellison, P. (2003a). Energetics and reproductive effort. American Journal of Human Biology, 15(3), 342–351. 12704710 10.1002/ajhb.10152

[ajhb23811-bib-0117] Ellison, P. T. (1988). Human salivary steroids: Methodological considerations and applications in physical anthropology. American Journal of Physical Anthropology, 31(S9), 115–142.

[ajhb23811-bib-0118] Ellison, P. T. (1996). Developmental influences on adult ovarian hormonal function. American Journal of Human Biology, 8(6), 725–734. 28561465 10.1002/(SICI)1520-6300(1996)8:6<725::AID-AJHB4>3.0.CO;2-S

[ajhb23811-bib-0119] Ellison, P. T. (2003b). Energetics and reproductive effort. American Journal of Human Biology, 15(3), 342–351. 12704710 10.1002/ajhb.10152

[ajhb23811-bib-0120] Ellison, P. T. (2017). Endocrinology, energetics, and human life history: A synthetic model. Hormones and Behavior, 91, 97–106. 27650355 10.1016/j.yhbeh.2016.09.006

[ajhb23811-bib-0121] Ellison, P. T. , Bribiescas, R. G. , Bentley, G. R. , Campbell, B. C. , Lipson, S. F. , Panter‐Brick, C. , & Hill, K. (2002). Population variation in age‐related decline in male salivary testosterone. Human Reproduction, 17(12), 3251–3253. 12456632 10.1093/humrep/17.12.3251

[ajhb23811-bib-0122] Ellison, P. T. , Lipson, S. F. , & Meredith, M. (1989). Salivary testosterone levels in males from the Ituri forest of Zaire. American Journal of Human Biology, 1(1), 21–24. 28514040 10.1002/ajhb.1310010106

[ajhb23811-bib-0123] Ellison, P. T. , & Panter‐Brick, C. (1996). Salivary testosterone levels among Tamang and Kami males of central Nepal. Human Biology, 68, 955–965. 8979466

[ajhb23811-bib-0124] Ellison, P. T. , Panter‐Brick, C. , Lipson, S. F. , & Rourke, M. T. (1993). The ecological context of human ovarian function. Human Reproduction, 8(12), 2248–2258. 8150934 10.1093/oxfordjournals.humrep.a138015

[ajhb23811-bib-0125] Ellison, P. T. , Peacock, N. R. , & Lager, C. (1986). Salivary progesterone and luteal function in two low‐fertility populations of Northeast Zaire. Human Biology, 58(4), 473–483. 3759049

[ajhb23811-bib-0126] Ellison, P. T. , & Valeggia, C. R. (2003). C‐peptide levels and the duration of lactational amenorrhea. Fertility and Sterility, 80(5), 1279–1280. 14607590 10.1016/s0015-0282(03)02158-7

[ajhb23811-bib-0127] Emery Thompson M. 2009. Dynamics of stress in wild female chimpanzees.1–34. 10.1016/j.yhbeh.2010.05.009 PMC3951729 20546741

[ajhb23811-bib-0128] Emery Thompson, M. (2017). Energetics of feeding, social behavior, and life history in non‐human primates. Hormones and Behavior, 91, 84–96. 27594442 10.1016/j.yhbeh.2016.08.009

[ajhb23811-bib-0129] Emery Thompson, M. , Fox, S. A. , Berghänel, A. , Sabbi, K. H. , Phillips‐Garcia, S. , Enigk, D. K. , Otali, E. , Machanda, Z. P. , Wrangham, R. W. , & Muller, M. N. (2020). Wild chimpanzees exhibit humanlike aging of glucocorticoid regulation. Proceedings of the National Academy of Sciences, 117(15), 8424–8430. 10.1073/pnas.1920593117 PMC7165472 32229565

[ajhb23811-bib-0130] Emery Thompson, M. , & Georgiev, A. V. (2014). The high price of success: Costs of mating effort in male primates. International Journal of Primatology, 35(3–4), 609–627.

[ajhb23811-bib-0131] Emery Thompson, M. , Jones, J. , Pusey, A. , Brewer‐Marsden, S. , Goodall, J. , Marsden, D. , Matsuzawa, T. , Nishida, T. , Reynolds, V. , & Sugiyama, Y. (2007). Aging and fertility patterns in wild chimpanzees provide insights into the evolution of menopause. Current Biology, 17(24), 2150–2156. 18083515 10.1016/j.cub.2007.11.033 PMC2190291

[ajhb23811-bib-0132] Emery Thompson, M. , & Knott, C. D. (2008). Urinary C‐peptide of insulin as a non‐invasive marker of energy balance in wild orangutans. Hormones and Behavior, 53(4), 526–535. 18255067 10.1016/j.yhbeh.2007.12.005

[ajhb23811-bib-0133] Emery Thompson, M. , Muller, M. N. , Kahlenberg, S. M. , & Wrangham, R. W. (2010). Dynamics of social and energetic stress in wild female chimpanzees. Hormones and Behavior, 58(3), 440–449. 20546741 10.1016/j.yhbeh.2010.05.009 PMC3951729

[ajhb23811-bib-0134] Emery Thompson, M. , Muller, M. N. , Sabbi, K. , Machanda, Z. P. , Otali, E. , & Wrangham, R. W. (2016). Faster reproductive rates trade off against offspring growth in wild chimpanzees. Proceedings of the National Academy of Sciences, 113(28), 7780–7785. 10.1073/pnas.1522168113 PMC4948337 27354523

[ajhb23811-bib-0135] Emery Thompson, M. , Muller, M. N. , & Wrangham, R. W. (2012). The energetics of lactation and the return to fecundity in wild chimpanzees. Behavioral Ecology, 23(6), 1234–1241.

[ajhb23811-bib-0136] Emery Thompson, M. , Muller, M. N. , Wrangham, R. W. , Lwanga, J. S. , & Potts, K. B. (2009). Urinary C‐peptide tracks seasonal and individual variation in energy balance in wild chimpanzees. Hormones and Behavior, 55(2), 299–305. 19084530 10.1016/j.yhbeh.2008.11.005

[ajhb23811-bib-0137] Emery Thompson, M. , Zhou, A. , & Knott, C. D. (2012). Low testosterone correlates with delayed development in male orangutans. PLoS One, 7(10), e47282. 23077585 10.1371/journal.pone.0047282 PMC3471841

[ajhb23811-bib-0138] Esteban, M. , & Castaño, A. (2009). Non‐invasive matrices in human biomonitoring: A review. Environment International, 35(2), 438–449. 18951632 10.1016/j.envint.2008.09.003

[ajhb23811-bib-0139] Ezenwa, V. O. , Stefan Ekernas, L. , & Creel, S. (2012). Unravelling complex associations between testosterone and parasite infection in the wild. Functional Ecology, 26(1), 123–133.

[ajhb23811-bib-0140] Fasano, A. (2011). Zonulin and its regulation of intestinal barrier function: The biological door to inflammation, autoimmunity, and cancer. Physiological Reviews, 91, 151–175. 21248165 10.1152/physrev.00003.2008

[ajhb23811-bib-0141] Fasano, A. (2012). Zonulin, regulation of tight junctions, and autoimmune diseases. Annals of the New York Academy of Sciences, 1258(1), 25–33. 22731712 10.1111/j.1749-6632.2012.06538.x PMC3384703

[ajhb23811-bib-0142] Fasano, A. (2020). All disease begins in the (leaky) gut: Role of zonulin‐mediated gut permeability in the pathogenesis of some chronic inflammatory diseases. F1000Research, 9, 9. 32051759 10.12688/f1000research.20510.1 PMC6996528

[ajhb23811-bib-0143] Fedurek, P. , Lacroix, L. , Lehmann, J. , Aktipis, A. , Cronk, L. , Townsend, C. , Makambi, E. J. , Mabulla, I. , Behrends, V. , & Berbesque, J. C. (2020). Status does not predict stress: Women in an egalitarian hunter–gatherer society. Evolutionary Human Sciences, 2, 1–15. 10.1017/ehs.2020.44 PMC10427491 37588349

[ajhb23811-bib-0144] Fischer, S. , Schumacher, S. , Skoluda, N. , & Strahler, J. (2020). Fingernail cortisol–state of research and future directions. Frontiers in Neuroendocrinology, 58, 100855. 32730860 10.1016/j.yfrne.2020.100855

[ajhb23811-bib-0145] Flatt, T. , & Heyland, A. (2011). Mechanisms of life history evolution: The genetics and physiology of life history traits and trade‐offs. Oxford University Press.

[ajhb23811-bib-0146] Flinn, M. V. (2010). Evolutionary biology of hormonal responses to social challenges in the human child. In M. P. Muehlenbein (Ed.), Human Evolutionary Biology (pp. 405). Cambridge University Press.

[ajhb23811-bib-0147] Flinn, M. V. , & England, B. G. (1997). Social economics of childhood glucocorticoid stress response and health. American Journal of Physical Anthropology, 102(1), 33–53. 9034037 10.1002/(SICI)1096-8644(199701)102:1<33::AID-AJPA4>3.0.CO;2-E

[ajhb23811-bib-0148] Foerster, S. , Cords, M. , & Monfort, S. L. (2012). Seasonal energetic stress in a tropical forest primate: Proximate causes and evolutionary implications. PLoS One, 7(11), e50108. 23209651 10.1371/journal.pone.0050108 PMC3509155

[ajhb23811-bib-0149] Fürtbauer, I. , Christensen, C. , Bracken, A. , O'Riain, M. J. , Heistermann, M. , & King, A. J. (2020). Energetics at the urban edge: Environmental and individual predictors of urinary C‐peptide levels in wild chacma baboons (*Papio ursinus*). Hormones and Behavior, 126, 104846. 32860833 10.1016/j.yhbeh.2020.104846

[ajhb23811-bib-0150] Gangestad, S. W. , Merriman, L. A. , & Emery, T. M. (2010). Men's oxidative stress, fluctuating asymmetry and physical attractiveness. Animal Behaviour, 80(6), 1005–1013.

[ajhb23811-bib-0151] Gao, W. , Stalder, T. , Foley, P. , Rauh, M. , Deng, H. , & Kirschbaum, C. (2013). Quantitative analysis of steroid hormones in human hair using a column‐switching LC–APCI–MS/MS assay. Journal of Chromatography B, 928, 1–8. 10.1016/j.jchromb.2013.03.008 23584040

[ajhb23811-bib-0152] García‐Cao, M. , Gonzalo, S. , Dean, D. , & Blasco, M. A. (2002). A role for the Rb family of proteins in controlling telomere length. Nature Genetics, 32(3), 415–419. 12379853 10.1038/ng1011

[ajhb23811-bib-0153] García‐Cao, M. , O'Sullivan, R. , Peters, A. H. F. M. , Jenuwein, T. , & Blasco, M. A. (2004). Epigenetic regulation of telomere length in mammalian cells by the Suv39h1 and Suv39h2 histone methyltransferases. Nature Genetics, 36(1), 94–99. 14702045 10.1038/ng1278

[ajhb23811-bib-0154] Garg, S. , Zheng, J. , Wang, J. , Authier, S. , Pouliot, M. , & Hauer‐Jensen, M. (2015). Segmental differences in radiation‐induced alterations of tight junction‐related proteins in Non‐human primate jejunum. Ileum and Colon. Radiation Research, 185(1), 50–59. 26720804 10.1667/RR14157.1 PMC4720531

[ajhb23811-bib-0155] Gesquiere, L. R. , Altmann, J. , Khan, M. Z. , Couret, J. , Yu, J. C. , Endres, C. S. , Lynch, J. W. , Ogola, P. , Fox, E. A. , & Alberts, S. C. (2005). Coming of age: Steroid hormones of wild immature baboons (*Papio cynocephalus*). American Journal of Primatology, 67(1), 83–100. 16163714 10.1002/ajp.20171

[ajhb23811-bib-0503] Gesquiere, L. R. , Khan, M., Shek, L., Wango, T. L., Wango, E. O., Alberts, S. C., & Altmann, J. (2008). Coping with a challenging environment: effects of seasonal variability and reproductive status on glucocorticoid concentrations of female baboons (Papio cynocephalus). Hormones and Behavior, 54(3), 410–416. 18514196 10.1016/j.yhbeh.2008.04.007 PMC2603603

[ajhb23811-bib-0156] Gesquiere, L. R. , Pugh, M. , Alberts, S. C. , & Markham, A. C. (2018). Estimation of energetic condition in wild baboons using fecal thyroid hormone determination. General and Comparative Endocrinology, 260, 9–17. 29427633 10.1016/j.ygcen.2018.02.004 PMC5856635

[ajhb23811-bib-0157] Gettler, L. T. (2016). Becoming DADS. Current Anthropology, 57(S13), S38–S51.

[ajhb23811-bib-0158] Gettler, L. T. (2020). Exploring evolutionary perspectives on human fatherhood and paternal biology: Testosterone as an exemplar. In Handbook of Fathers and Child Development (pp. 137–152). Springer.

[ajhb23811-bib-0159] Gettler, L. T. , Lew‐Levy, S. , Sarma, M. S. , Miegakanda, V. , Doxsey, M. , Meyer, J. S. , & Boyette, A. H. (2021). Children's fingernail cortisol among BaYaka foragers of the Congo Basin: Associations with fathers' roles. Philosophical transactions of the Royal Society of London Series B, Biological sciences, 376(1827), 20200031. 33938276 10.1098/rstb.2020.0031 PMC8090812

[ajhb23811-bib-0160] Gettler, L. T. , McDade, T. W. , Agustin, S. S. , Feranil, A. B. , & Kuzawa, C. W. (2014). Testosterone, immune function, and life history transitions in Filipino males (*Homo sapiens*). International Journal of Primatology, 35(3), 787–804.

[ajhb23811-bib-0161] Gettler, L. T. , McDade, T. W. , Agustin, S. S. , Feranil, A. B. , & Kuzawa, C. W. (2015). Longitudinal perspectives on fathers' residence status, time allocation, and testosterone in The Philippines. Adaptive Human Behavior and Physiology, 1(2), 124–149.

[ajhb23811-bib-0162] Gettler, L. T. , Mcdade, T. W. , Feranil, A. B. , & Kuzawa, C. W. (2011a). Longitudinal evidence that fatherhood decreases testosterone in human males. Proceedings of the National Academy of Sciences, 108(39), 16194–16199. 10.1073/pnas.1105403108 PMC3182719 21911391

[ajhb23811-bib-0163] Gettler, L. T. , McDade, T. W. , Feranil, A. B. , & Kuzawa, C. W. (2011b). Longitudinal evidence that fatherhood decreases testosterone in human males. Proceedings of the National Academy of Sciences, 108(39), 16194–16199. 10.1073/pnas.1105403108 PMC3182719 21911391

[ajhb23811-bib-0164] Giavedoni, L. D. (2005). Simultaneous detection of multiple cytokines and chemokines from nonhuman primates using luminex technology. Journal of Immunological Methods, 301(1–2), 89–101. 15896800 10.1016/j.jim.2005.03.015

[ajhb23811-bib-0165] Giesbrecht, G. F. , Campbell, T. , & Letourneau, N. (2015). Sexually dimorphic adaptations in basal maternal stress physiology during pregnancy and implications for fetal development. Psychoneuroendocrinology, 56, 168–178. 25827961 10.1016/j.psyneuen.2015.03.013

[ajhb23811-bib-0166] Gildner, T. E. (2021a). Reproductive hormone measurement from minimally invasive sample types: Methodological considerations and anthropological importance. American Journal of Human Biology, 33(1), e23535. 33174269 10.1002/ajhb.23535

[ajhb23811-bib-0167] Gildner, T. E. (2021b). Reproductive hormone measurement from minimally invasive sample types: Methodological considerations and anthropological importance. American Journal of Human Biology, 33(1), e23535. 33174269 10.1002/ajhb.23535

[ajhb23811-bib-0168] Gildner, T. E. , Eick, G. N. , Schneider, A. L. , Madimenos, F. C. , & Snodgrass, J. J. (2021). After Theranos: Using point‐of‐care testing to advance measures of health biomarkers in human biology research. American Journal of Human Biology, 1–28. 10.1002/ajhb.23689 34669210

[ajhb23811-bib-0169] Girard‐Buttoz, C. , Heistermann, M. , Rahmi, E. , Agil, M. , Fauzan, P. A. , & Engelhardt, A. (2015). Androgen correlates of male reproductive effort in wild male long‐tailed macaques (*Macaca fascicularis*): A multi‐level test of the challenge hypothesis. Physiology & Behavior, 141, 143–153. 25596329 10.1016/j.physbeh.2015.01.015

[ajhb23811-bib-0170] Girard‐Buttoz, C. , Higham, J. P. , Heistermann, M. , Wedegärtner, S. , Maestripieri, D. , & Engelhardt, A. (2011). Urinary C‐peptide measurement as a marker of nutritional status in macaques. PLoS One, 6(3), e18042. 21479215 10.1371/journal.pone.0018042 PMC3068145

[ajhb23811-bib-0171] Gluckman, P. , Hanson, M. , & Spencer, H. (2005). Predictive adaptive responses and human evolution. Trends in Ecology & Evolution, 20(10), 527–533. 16701430 10.1016/j.tree.2005.08.001

[ajhb23811-bib-0172] Goldman, E. A. , Eick, G. N. , Compton, D. , Kowal, P. , Snodgrass, J. J. , Eisenberg, D. T. A. , & Sterner, K. N. (2018). Evaluating minimally invasive sample collection methods for telomere length measurement. American Journal of Human Biology, 30(1), e23062. 10.1002/ajhb.23062 PMC5785450 28949426

[ajhb23811-bib-0173] González, N. T. , Otali, E. , Machanda, Z. , Muller, M. N. , Wrangham, R. , & Thompson, M. E. (2020). Urinary markers of oxidative stress respond to infection and late‐life in wild chimpanzees. PLoS One, 15(9), e0238066. 32916689 10.1371/journal.pone.0238066 PMC7486137

[ajhb23811-bib-0174] Gonzalo, S. , García‐Cao, M. , Fraga, M. F. , Schotta, G. , Peters, A. H. F. M. , Cotter, S. E. , Eguía, R. , Dean, D. C. , Esteller, M. , & Jenuwein, T. (2005). Role of the RB1 family in stabilizing histone methylation at constitutive heterochromatin. Nature Cell Biology, 7(4), 420–428. 15750587 10.1038/ncb1235

[ajhb23811-bib-0175] Gonzalo, S. , Jaco, I. , Fraga, M. F. , Chen, T. , Li, E. , Esteller, M. , & Blasco, M. A. (2006). DNA methyltransferases control telomere length and telomere recombination in mammalian cells. Nature Cell Biology, 8(4), 416–424. 16565708 10.1038/ncb1386

[ajhb23811-bib-0176] Grange, R. , Thompson, J. , & Lambert, D. (2014). Radioimmunoassay, enzyme and non‐enzyme‐based immunoassays. British Journal of Anaesthesia, 112(2), 213–216. 24431350 10.1093/bja/aet293

[ajhb23811-bib-0177] Gray, P. B. (2003). Marriage, parenting, and testosterone variation among Kenyan Swahili men. American Journal of Physical Anthropology: The Official Publication of the American Association of Physical Anthropologists, 122(3), 279–286. 10.1002/ajpa.10293 14533186

[ajhb23811-bib-0178] Gray, P. B. , & Campbell, B. C. (2009). Human male testosterone, pair‐bonding. In P. T. Eliison, & P. B. Gray (Eds.), Endocrinology of Social Relationships (pp. 270). Harvard University Press.

[ajhb23811-bib-0179] Gray, P. B. , Ellison, P. T. , & Campbell, B. C. (2007). Testosterone and marriage among Ariaal men of northern Kenya. Current Anthropology, 48(5), 750–755.

[ajhb23811-bib-0180] Gray, P. B. , McHale, T. S. , & Carré, J. M. (2017). A review of human male field studies of hormones and behavioral reproductive effort. Hormones and Behavior, 91, 52–67. 27449532 10.1016/j.yhbeh.2016.07.004

[ajhb23811-bib-0181] Gray, P. B. , Straftis, A. A. , Bird, B. M. , McHale, T. S. , & Zilioli, S. (2020). Human reproductive behavior, life history, and the challenge hypothesis: A 30‐year review, retrospective and future directions. Hormones and Behavior, 123, 104530. 31085183 10.1016/j.yhbeh.2019.04.017

[ajhb23811-bib-0182] Greisen, S. , Ledet, T. , & Ovesen, P. (2001). Effects of androstenedione, insulin and luteinizing hormone on steroidogenesis in human granulosa luteal cells. Human Reproduction, 16(10), 2061–2065. 11574492 10.1093/humrep/16.10.2061

[ajhb23811-bib-0183] Grey, K. R. , Davis, E. P. , Sandman, C. A. , & Glynn, L. M. (2013). Human milk cortisol is associated with infant temperament. Psychoneuroendocrinology, 38(7), 1178–1185. 23265309 10.1016/j.psyneuen.2012.11.002 PMC4777694

[ajhb23811-bib-0184] Gröschl, M. , Rauh, M. , & Dörr, H.‐G. (2003). Circadian rhythm of salivary cortisol, 17α‐hydroxyprogesterone, and progesterone in healthy children. Clinical Chemistry, 49(10), 1688–1691. 14500602 10.1373/49.10.1688

[ajhb23811-bib-0185] Grueter, C. C. , Deschner, T. , Behringer, V. , Fawcett, K. , & Robbins, M. M. (2014a). Socioecological correlates of energy balance using urinary C‐peptide measurements in wild female mountain gorillas. Physiology & Behavior, 127, 13–19. 24472322 10.1016/j.physbeh.2014.01.009

[ajhb23811-bib-0186] Grueter, C. C. , Deschner, T. , Behringer, V. , Fawcett, K. , & Robbins, M. M. (2014b). Socioecological correlates of energy balance using urinary C‐peptide measurements in wild female mountain gorillas. Physiology & Behavior, 127, 13–19. 24472322 10.1016/j.physbeh.2014.01.009

[ajhb23811-bib-0187] Guerrant, R. L. , Leite, A. M. , Pinkerton, R. , Medeiros, P. H. , Cavalcante, P. A. , DeBoer, M. , Kosek, M. , Duggan, C. , Gewirtz, A. , & Kagan, J. C. (2016a). Biomarkers of environmental enteropathy, inflammation, stunting, and impaired growth in children in Northeast Brazil. PLoS One, 11(9), e0158772. 27690129 10.1371/journal.pone.0158772 PMC5045163

[ajhb23811-bib-0188] Guerrant, R. L. , Leite, A. M. , Pinkerton, R. , Medeiros, P. H. Q. S. , Cavalcante, P. A. , DeBoer, M. , Kosek, M. , Duggan, C. , Gewirtz, A. , & Kagan, J. C. (2016b). Biomarkers of environmental enteropathy, inflammation, stunting, and impaired growth in children in Northeast Brazil. PLoS One, 11(9), e0158772. 27690129 10.1371/journal.pone.0158772 PMC5045163

[ajhb23811-bib-0189] Gunnar, M. , & Quevedo, K. (2007). The neurobiology of stress and development. Annual Review of Psychology, 58, 145–173. 10.1146/annurev.psych.58.110405.085605 16903808

[ajhb23811-bib-0190] Günzel, D. , & Yu, A. S. (2013). Claudins and the modulation of tight junction permeability. Physiological Reviews, 93(2), 525–569. 23589827 10.1152/physrev.00019.2012 PMC3768107

[ajhb23811-bib-0191] Gurven, M. , Kaplan, H. , Winking, J. , Finch, C. , & Crimmins, E. M. (2008). Aging and inflammation in two epidemiological worlds. The Journals of Gerontology Series A: Biological Sciences and Medical Sciences, 63(2), 196–199. 18314457 10.1093/gerona/63.2.196 PMC2952348

[ajhb23811-bib-0192] Gurven, M. , Kaplan, H. , Winking, J. , Rodriguez, D. E. , Vasunilashorn, S. , Kim, J. K. , Finch, C. , & Crimmins, E. (2009). Inflammation and infection do not promote arterial aging and cardiovascular disease risk factors among lean horticulturalists. PLoS One, 4(8), e6590. 19668697 10.1371/journal.pone.0006590 PMC2722089

[ajhb23811-bib-0193] Hahn‐Holbrook, J. , Le, T. B. , Chung, A. , Davis, E. P. , & Glynn, L. M. (2016). Cortisol in human milk predicts child BMI. Obesity, 24(12), 2471–2474. 27891832 10.1002/oby.21682 PMC5400496

[ajhb23811-bib-0194] Halleen, J. M. , Tiitinen, S. L. , Ylipahkala, H. , Fagerlund, K. M. , & Vaananen, H. K. (2006). Tartrate‐resistant acid phosphatase 5b (TRACP 5b) as a marker of bone resorption. Clinical Laboratory, 52(9–10), 499–509. 17078477

[ajhb23811-bib-0195] Harper, K. M. , Mutasa, M. , Prendergast, A. J. , Humphrey, J. , & Manges, A. R. (2018). Environmental enteric dysfunction pathways and child stunting: A systematic review. PLoS Neglected Tropical Diseases, 12(1), e0006205. 29351288 10.1371/journal.pntd.0006205 PMC5792022

[ajhb23811-bib-0196] Harris, K. M. , & Schorpp, K. M. (2018). Integrating biomarkers in social stratification and health research. Annual Review of Sociology, 44(1), 361–386. 10.1146/annurev-soc-060116-053339 PMC6433161 30918418

[ajhb23811-bib-0197] Harris, T. R. , Chapman, C. A. , & Monfort, S. L. (2010). Small folivorous primate groups exhibit behavioral and physiological effects of food scarcity. Behavioral Ecology, 21(1), 46–56.

[ajhb23811-bib-0198] Hawkes, K. (2003). Grandmothers and the evolution of human longevity. American Journal of Human Biology, 15(3), 380–400. 12704714 10.1002/ajhb.10156

[ajhb23811-bib-0199] Hawley, P. H. (1999). The ontogenesis of social dominance: A strategy‐based evolutionary perspective. Developmental Review, 19(1), 97–132.

[ajhb23811-bib-0200] Heistermann, M. , & Higham, J. P. (2015). Urinary neopterin, a non‐invasive marker of mammalian cellular immune activation, is highly stable under field conditions. Scientific Reports, 5(1), 16308. 26549509 10.1038/srep16308 PMC4637859

[ajhb23811-bib-0201] Helfrecht, C. , Hagen, E. H. , DeAvila, D. , Bernstein, R. M. , Dira, S. J. , & Meehan, C. L. (2018). DHEAS patterning across childhood in three sub‐Saharan populations: Associations with age, sex, ethnicity, and cortisol. American Journal of Human Biology, 30(2), e23090. 10.1002/ajhb.23090 29226590

[ajhb23811-bib-0202] Hendy, O. M. , Elsabaawy, M. M. , Aref, M. M. , Khalaf, F. M. , Oda, A. M. A. , & El Shazly, H. M. (2017). Evaluation of circulating zonulin as a potential marker in the pathogenesis of nonalcoholic fatty liver disease. APMIS, 125(7), 607–613. 28430371 10.1111/apm.12696

[ajhb23811-bib-0203] Henrich, J. , & Gil‐White, F. J. (2001). The evolution of prestige: Freely conferred deference as a mechanism for enhancing the benefits of cultural transmission. Evolution and Human Behavior, 22(3), 165–196. 11384884 10.1016/s1090-5138(00)00071-4

[ajhb23811-bib-0204] Herrmann, M. , & Seibel, M. (2008). The amino‐and carboxyterminal cross‐linked telopeptides of collagen type I, NTX‐I and CTX‐I: A comparative review. Clinica Chimica Acta, 393(2), 57–75. 10.1016/j.cca.2008.03.020 18423400

[ajhb23811-bib-0205] Higham, J. P. (2016). Field endocrinology of nonhuman primates: Past, present, and future. Hormones and Behavior, 84, 145–155. 27469069 10.1016/j.yhbeh.2016.07.001

[ajhb23811-bib-0206] Higham, J. P. , Girard‐Buttoz, C. , Engelhardt, A. , & Heistermann, M. (2011). Urinary C‐peptide of insulin as a non‐invasive marker of nutritional status: Some practicalities. PLoS One, 6(7), e22398. 21799844 10.1371/journal.pone.0022398 PMC3142156

[ajhb23811-bib-0207] Higham, J. P. , Heistermann, M. , & Maestripieri, D. (2011). The energetics of male–male endurance rivalry in free‐ranging rhesus macaques, *Macaca mulatta* . Animal Behaviour, 81(5), 1001–1007.

[ajhb23811-bib-0208] Higham, J. P. , Heistermann, M. , & Maestripieri, D. (2013). The endocrinology of male rhesus macaque social and reproductive status: A test of the challenge and social stress hypotheses. Behavioral Ecology and Sociobiology, 67(1), 19–30. 24634561 10.1007/s00265-012-1420-6 PMC3950204

[ajhb23811-bib-0209] Higham, J. P. , Kraus, C. , Stahl‐Hennig, C. , Engelhardt, A. , Fuchs, D. , & Heistermann, M. (2015). Evaluating noninvasive markers of nonhuman primate immune activation and inflammation. American Journal of Physical Anthropology, 158(4), 673–684. 26250063 10.1002/ajpa.22821

[ajhb23811-bib-0210] Higham, J. P. , Stahl‐Hennig, C. , & Heistermann, M. (2020). Urinary suPAR: A non‐invasive biomarker of infection and tissue inflammation for use in studies of large free‐ranging mammals. Royal Society Open Science, 7(2), 191825. 32257339 10.1098/rsos.191825 PMC7062102

[ajhb23811-bib-0211] Hinde, K. , & Milligan, L. A. (2011). Primate milk: Proximate mechanisms and ultimate perspectives. Evolutionary Anthropology: Issues, News, and Reviews, 20(1), 9–23. 10.1002/evan.20289 22034080

[ajhb23811-bib-0212] Hinde, K. , Skibiel, A. L. , Foster, A. B. , Del Rosso, L. , Mendoza, S. P. , & Capitanio, J. P. (2015). Cortisol in mother's milk across lactation reflects maternal life history and predicts infant temperament. Behavioral Ecology, 26(1), 269–281. 25713475 10.1093/beheco/aru186 PMC4309982

[ajhb23811-bib-0213] Hochberg, Z. (2011). Evo‐devo of child growth: Treatise on child growth and human evolution. John Wiley & Sons.

[ajhb23811-bib-0214] Hoffman, C. L. , Higham, J. P. , Heistermann, M. , Coe, C. L. , Prendergast, B. J. , & Maestripieri, D. (2011). Immune function and HPA axis activity in free‐ranging rhesus macaques. Physiology & Behavior, 104(3), 507–514. 21635909 10.1016/j.physbeh.2011.05.021 PMC3133459

[ajhb23811-bib-0215] Hoke, M. K. , McCabe, K. A. , Miller, A. A. , & McDade, T. W. (2018). Validation of endotoxin‐core antibodies in dried blood spots as a measure of environmental enteropathy and intestinal permeability. American Journal of Human Biology, 30(4), e23120. 29532544 10.1002/ajhb.23120

[ajhb23811-bib-0216] Hollegaard, M. V. , Grauholm, J. , Nørgaard‐Pedersen, B. , & Hougaard, D. M. (2013). DNA methylome profiling using neonatal dried blood spot samples: A proof‐of‐principle study. Molecular Genetics and Metabolism, 108(4), 225–231. 23422032 10.1016/j.ymgme.2013.01.016

[ajhb23811-bib-0217] Holst, J. J. , Hartmann, B. , Gottschalck, I. B. , Jeppesen, P. B. , Miholic, J. , & Bang, H. D. (2007). Bone resorption is decreased postprandially by intestinal factors and glucagon‐like peptide‐2 is a possible candidate. Scandinavian Journal of Gastroenterology, 42(7), 814–820. 17558904 10.1080/00365520601137272

[ajhb23811-bib-0218] Jaeggi, A. V. , Blackwell, A. D. , Von Rueden, C. , Trumble, B. C. , Stieglitz, J. , Garcia, A. R. , Kraft, T. S. , Beheim, B. A. , Hooper, P. L. , & Kaplan, H. (2021). Do wealth and inequality associate with health in a small‐scale subsistence society? eLife, 10, 1–28. 10.7554/eLife.59437 PMC8225390 33988506

[ajhb23811-bib-0501] Jaimez, N. A ., Bribiescas, R. G., Aronsen, G. P., Anestis, S. A., & Watts, D. P. (2012). Urinary cortisol levels of gray‐cheeked mangabeys are higher in disturbed compared to undisturbed forest areas in K ibale N ational P ark, U ganda. Animal Conservation, 15(3), 242–247.

[ajhb23811-bib-0219] Janckila, A. J. , & Yam, L. T. (2009). Biology and clinical significance of tartrate‐resistant acid phosphatases: New perspectives on an old enzyme. Calcified Tissue International, 85(6), 465–483. 19915788 10.1007/s00223-009-9309-8

[ajhb23811-bib-0220] Jaramillo Ortiz, S. , Howsam, M. , van Aken, E. H. , Delanghe, J. R. , Boulanger, E. , & Tessier, F. J. (2021). Biomarkers of disease in human nails: A comprehensive review. Critical Reviews in Clinical Laboratory Sciences, 59, 1–17. 34726550 10.1080/10408363.2021.1991882

[ajhb23811-bib-0221] Jasienska, G. , Bribiescas, R. G. , Furberg, A.‐S. , Helle, S. , & Núñez‐de la Mora, A. (2017). Human reproduction and health: An evolutionary perspective. The Lancet, 390(10093), 510–520. 10.1016/S0140-6736(17)30573-1 28792413

[ajhb23811-bib-0222] Jasieńska, G. , & Ellison, P. T. (1998). Physical work causes suppression of ovarian function in women. Proceedings of the Royal Society of London Series B: Biological Sciences, 265(1408), 1847–1851. 10.1098/rspb.1998.0511 PMC1689377 9802241

[ajhb23811-bib-0223] Jasienska, G. , & Ellison, P. T. (2004). Energetic factors and seasonal changes in ovarian function in women from rural Poland. American Journal of Human Biology, 16(5), 563–580. 15368604 10.1002/ajhb.20063

[ajhb23811-bib-0224] Jasienska, G. , & Jasienski, M. (2008). Interpopulation, interindividual, intercycle, and intracycle natural variation in progesterone levels: A quantitative assessment and implications for population studies. American Journal of Human Biology, 20(1), 35–42. 17963226 10.1002/ajhb.20686

[ajhb23811-bib-0225] Jasienska, G. , Ziomkiewicz, A. , Lipson, S. F. , Thune, I. , & Ellison, P. T. (2005). High ponderal index at birth predicts high estradiol levels in adult women. American Journal of Human Biology, 18(1), 133–140. 10.1002/ajhb.20462 16378335

[ajhb23811-bib-0226] Jatkoe, T. A. , Karnes, R. J. , Freedland, S. J. , Wang, Y. , Le, A. , & Baden, J. (2015). A urine‐based methylation signature for risk stratification within low‐risk prostate cancer. British Journal of Cancer, 112(5), 802–808. 25695483 10.1038/bjc.2015.7 PMC4453961

[ajhb23811-bib-0227] Jones, J. H. (2011). Primates and the evolution of long, slow life histories. Current Biology, 21(18), R708–R717. 21959161 10.1016/j.cub.2011.08.025 PMC3192902

[ajhb23811-bib-0228] Kaplan, H. , Hill, K. , Lancaster, J. , & Hurtado, A. (2000). A theory of human life history evolution: Diet, intelligence, and longevity. Evolutionary Anthropology: Issues, News, and Reviews, 9(4), 156–185.

[ajhb23811-bib-0229] Keestra, S. M. , Bentley, G. R. , Núñez‐de la Mora, A. , Houghton, L. C. , Wilson, H. , Vázquez‐Vázquez, A. , Cooper, G. D. , Dickinson, F. , Griffiths, P. , & Bogin, B. A. (2021). The timing of adrenarche in Maya girls, Merida, Mexico. American Journal of Human Biology, 33(2), e23465. 32643208 10.1002/ajhb.23465 PMC8264844

[ajhb23811-bib-0230] Keevil, B. G. , Fiers, T. , Kaufman, J.‐M. , Macdowall, W. , Clifton, S. , Lee, D. , & Wu, F. (2016). Sex hormone‐binding globulin has no effect on salivary testosterone. Annals of Clinical Biochemistry, 53(6), 717–720. 10.1177/0004563216646800 27117450

[ajhb23811-bib-0231] Key, C. , & Ross, C. (1999). Sex differences in energy expenditure in non–human primates. Proceedings of the Royal Society of London Series B: Biological Sciences, 266(1437), 2479–2485. 10.1098/rspb.1999.0949 PMC1690481 10693818

[ajhb23811-bib-0232] Kim, A. W. , Adam, E. K. , Bechayda, S. A. , & Kuzawa, C. W. (2020). Early life stress and HPA axis function independently predict adult depressive symptoms in metropolitan Cebu, Philippines. American Journal of Physical Anthropology, 173(3), 448–462. 32744374 10.1002/ajpa.24105 PMC7846226

[ajhb23811-bib-0233] Klein, L. D. , Huang, J. , Quinn, E. A. , Martin, M. A. , Breakey, A. A. , Gurven, M. , Kaplan, H. , Valeggia, C. , Jasienska, G. , & Scelza, B. (2018). Variation among populations in the immune protein composition of mother's milk reflects subsistence pattern. Evolution, Medicine, and Public Health, 2018(1), 230–245. 30430010 10.1093/emph/eoy031 PMC6222208

[ajhb23811-bib-0234] Klibanski, A. , Beitins, I. Z. , Badger, T. , Little, R. , & McArthur, J. W. (1981). Reproductive function during fasting in men. The Journal of Clinical Endocrinology & Metabolism, 53(2), 258–263. 6788791 10.1210/jcem-53-2-258

[ajhb23811-bib-0235] Knott, C. D. (1997). Field collection and preservation of urine in orangutans and Chimpanzees. Tropical Biodiversity, 4(1), 95–102.

[ajhb23811-bib-0236] Knott, C. D. (1998). Changes in orangutan caloric intake, energy balance, and ketones in response to fluctuating fruit availability. International Journal of Primatology, 19(6), 1061–1079.

[ajhb23811-bib-0237] Knott, C. D. (1999). Reproductive, physiological and behavioral responses of orangutans in Borneo to fluctuations in food availability. Harvard University.

[ajhb23811-bib-0238] Konečná, M. , & Urlacher, S. S. (2017). Male social status and its predictors among Garisakang forager‐horticulturalists of lowland Papua New Guinea. Evolution and Human Behavior, 38(6), 789–797.

[ajhb23811-bib-0239] Kordsmeyer, T. L. , Freund, D. , Vugt, M. V. , & Penke, L. (2019). Honest signals of status: Facial and bodily dominance are related to success in physical but not nonphysical competition. Evolutionary Psychology, 17(3), 147470491986316. 10.1177/1474704919863164 PMC10358418 31345060

[ajhb23811-bib-0240] Kosek, M. N. (2017). Causal pathways from enteropathogens to environmental enteropathy: Findings from the MAL‐ED birth cohort study. eBioMedicine, 18, 109–117. 28396264 10.1016/j.ebiom.2017.02.024 PMC5405169

[ajhb23811-bib-0241] Kruger, D. J. , & Nesse, R. M. (2006). An evolutionary life‐history framework for understanding sex differences in human mortality rates. Human Nature, 17(1), 74–97. 26181346 10.1007/s12110-006-1021-z

[ajhb23811-bib-0242] Kruszynska, Y. , Home, P. , Hanning, I. , & Alberti, K. (1987). Basal and 24‐h C‐peptide and insulin secretion rate in normal man. Diabetologia, 30(1), 16–21. 3552817 10.1007/BF01788901

[ajhb23811-bib-0243] Küme, T. , Acar, S. , Tuhan, H. , Çatlı, G. , Anık, A. , Çalan, Ö. G. , Böber, E. , & Abacı, A. (2017). The relationship between serum zonulin level and clinical and laboratory parameters of childhood obesity. Journal of Clinical Research in Pediatric Endocrinology, 9(1), 31–38. 28008865 10.4274/jcrpe.3682 PMC5363162

[ajhb23811-bib-0244] Kuo, T. R. , & Chen, C. H. (2017). Bone biomarker for the clinical assessment of osteoporosis: Recent developments and future perspectives. Biomarker Research, 5, 18. 28529755 10.1186/s40364-017-0097-4 PMC5436437

[ajhb23811-bib-0245] Kuzawa, C. W. , & Bragg, J. M. (2013). Plasticity in human life history strategy. Current Anthropology, 53(S6), 1–14.

[ajhb23811-bib-0246] Kuzawa, C. W. , Gettler, L. T. , Muller, M. N. , Mcdade, T. W. , & Feranil, A. B. (2009). Fatherhood, pairbonding and testosterone in the Philippines. Hormones and Behavior, 56(4), 429–435. 19651129 10.1016/j.yhbeh.2009.07.010 PMC2855897

[ajhb23811-bib-0247] Kuzawa, C. W. , & Sweet, E. (2009). Epigenetics and the embodiment of race: Developmental origins of US racial disparities in cardiovascular health. American Journal of Human Biology, 21(1), 2–15. 18925573 10.1002/ajhb.20822

[ajhb23811-bib-0248] Langie, S. A. S. , Moisse, M. , Declerck, K. , Koppen, G. , Godderis, L. , Vanden Berghe, W. , Drury, S. , & De Boever, P. (2017). Salivary DNA methylation profiling: Aspects to consider for biomarker identification. Basic & Clinical Pharmacology & Toxicology, 121(S3), 93–101. 27901320 10.1111/bcpt.12721 PMC5644718

[ajhb23811-bib-0249] Lea, A. J. , Altmann, J. , Alberts, S. C. , & Tung, J. (2016). Resource base influences genome‐wide DNA methylation levels in wild baboons (*Papio cynocephalus*). Molecular Ecology, 25(8), 1681–1696. 26508127 10.1111/mec.13436 PMC4846536

[ajhb23811-bib-0250] Lea, A. J. , Tung, J. , Archie, E. A. , & Alberts, S. C. (2017). Developmental plasticity. Evolution, Medicine, and Public Health, 2017(1), 162–175. 29424834 10.1093/emph/eox019 PMC5798083

[ajhb23811-bib-0251] Lee, A. J. , Hodges, S. , & Eastell, R. (2000). Measurement of osteocalcin. Annals of Clinical Biochemistry, 37(4), 432–446. 10902858 10.1177/000456320003700402

[ajhb23811-bib-0252] Leigh, S. R. (2001). Evolution of human growth. Evolutionary Anthropology, 10, 1–14.

[ajhb23811-bib-0253] Leonard, W. R. , Snodgrass, J. J. , & Sorensen, M. V. (2005). Metabolic adaptation in indigenous Siberian populations. Annual Review of Anthropology, 34(1), 451–471.

[ajhb23811-bib-0254] Leonard, W. R. , Sorensen, M. V. , Galloway, V. A. , Spencer, G. J. , Mj, M. , Osipova, L. , & Spitsyn, V. A. (2002). Climatic influences on basal metabolic rates among circumpolar populations. American Journal of Human Biology, 14(5), 609–620. 12203815 10.1002/ajhb.10072

[ajhb23811-bib-0255] Levine, M. , Duffy, L. , Moore, D. C. , & Matej, L. A. (1995). Acclimation of a non‐indigenous sub‐Arctic population: Seasonal variation in thyroid function in interior Alaska. Comparative Biochemistry and Physiology Part A: Physiology, 111(2), 209–214. 7788348 10.1016/0300-9629(95)00016-z

[ajhb23811-bib-0256] Levis, R. , Hazelrigg, T. , & Rubin, G. M. (1985). Effects of genomic position on the expression of transduced copies of the white gene of drosophila. Science, 229(4713), 558–561. 2992080 10.1126/science.2992080

[ajhb23811-bib-0257] Levy, S. B. , Klimova, T. M. , Zakharova, R. N. , Federov, A. I. , Fedorova, V. I. , Baltakhinova, M. E. , & Leonard, W. R. (2018). Brown adipose tissue, energy expenditure, and biomarkers of cardio‐metabolic health among the Yakut (Sakha) of northeastern Siberia. American Journal of Human Biology, 30(6), e23175. 30368978 10.1002/ajhb.23175

[ajhb23811-bib-0258] Li, X. , & Atkinson, M. A. (2015). The role for gut permeability in the pathogenesis of type 1 diabetes–a solid or leaky concept? Pediatric Diabetes, 16(7), 485–492. 26269193 10.1111/pedi.12305 PMC4638168

[ajhb23811-bib-0259] Lieberman, D. E. , Kistner, T. M. , Richard, D. , Lee, I.‐M. , & Baggish, A. L. (2021). The active grandparent hypothesis: Physical activity and the evolution of extended human healthspans and lifespans. Proceedings of the National Academy of Sciences, 118(50), e2107621118. 10.1073/pnas.2107621118 PMC8685690 34810239

[ajhb23811-bib-0260] Liebert, M. A. , Snodgrass, J. J. , Madimenos, F. C. , Cepon, T. J. , Blackwell, A. D. , & Sugiyama, L. S. (2013). Implications of market integration for cardiovascular and metabolic health among an indigenous Amazonian Ecuadorian population. Annals of Human Biology, 40, 228–242. 23388068 10.3109/03014460.2012.759621

[ajhb23811-bib-0261] Lin, C.‐M. , Fan, H.‐C. , Chao, T.‐Y. , Chu, D.‐M. , Lai, C.‐C. , Wang, C.‐C. , & Chen, S.‐J. (2016). Potential effects of valproate and oxcarbazepine on growth velocity and bone metabolism in epileptic children‐a medical center experience. BMC Pediatrics, 16(1), 1–6. 27142370 10.1186/s12887-016-0597-7 PMC4855910

[ajhb23811-bib-0262] Lindau ST , McDade TW . 2008. Minimally invasive and innovative methods for biomeasure collection in population‐based research. Biosocial surveys: National Academies Press.

[ajhb23811-bib-0263] Lipson, S. F. , & Ellison, P. T. (1992). Normative study of age variation in salivary progesterone profiles. Journal of Biosocial Science, 24(2), 233–244. 1583036 10.1017/s0021932000019751

[ajhb23811-bib-0264] Little, R. R. , Rohlfing, C. L. , Tennill, A. L. , Madsen, R. W. , Polonsky, K. S. , Myers, G. L. , Greenbaum, C. J. , Palmer, J. P. , Rogatsky, E. , & Stein, D. T. (2008). Standardization of C‐peptide measurements. Clinical Chemistry, 54(6), 1023–1026. 18420730 10.1373/clinchem.2007.101287

[ajhb23811-bib-0265] Lodge, E. , Ross, C. , Ortmann, S. , & MacLarnon, A. (2013). Influence of diet and stress on reproductive hormones in Nigerian olive baboons. General and Comparative Endocrinology, 191, 146–154. 23800561 10.1016/j.ygcen.2013.06.016

[ajhb23811-bib-0266] López, M. , Alvarez, C. V. , Nogueiras, R. , & Diéguez, C. (2013). Energy balance regulation by thyroid hormones at central level. Trends in Molecular Medicine, 19(7), 418–427. 23707189 10.1016/j.molmed.2013.04.004

[ajhb23811-bib-0267] López, M. , Varela, L. , Vázquez, M. J. , Rodríguez‐Cuenca, S. , González, C. R. , Velagapudi, V. R. , Morgan, D. A. , Schoenmakers, E. , Agassandian, K. , & Lage, R. (2010). Hypothalamic AMPK and fatty acid metabolism mediate thyroid regulation of energy balance. Nature Medicine, 16(9), 1001–1008. 10.1038/nm.2207 PMC2935934 20802499

[ajhb23811-bib-0268] Lu, A. , Petrullo, L. , Carrera, S. , Feder, J. , Schneider‐Crease, I. , & Snyder‐Mackler, N. (2019). Developmental responses to early‐life adversity: Evolutionary and mechanistic perspectives. Evolutionary Anthropology: Issues, News, and Reviews, 28(5), 249–266. 10.1002/evan.21791 31498945

[ajhb23811-bib-0269] Lynn, C. D. , Howells, M. , Herdrich, D. , Ioane, J. , Hudson, D. , & Fitiao, S. T. U. (2020). The evolutionary adaptation of body art: Tattooing as costly honest signaling of enhanced immune response in American Samoa. American Journal of Human Biology: The Official Journal of the Human Biology Council, 32(4), e23347. 31654543 10.1002/ajhb.23347

[ajhb23811-bib-0270] Magid, K. , Chatterton, R. T. , Ahamed, F. U. , & Bentley, G. R. (2018). Childhood ecology influences salivary testosterone, pubertal age and stature of Bangladeshi UK migrant men. Nature Ecology and Evolution, 2(7), 1146–1154. 29942016 10.1038/s41559-018-0567-6

[ajhb23811-bib-0271] Majolo, B. , Lehmann, J. , de Bortoli, V. A. , & Schino, G. (2012). Fitness‐related benefits of dominance in primates. American Journal of Physical Anthropology, 147(4), 652–660. 22331647 10.1002/ajpa.22031

[ajhb23811-bib-0272] Mantzouratou, P. , Lavecchia, A. M. , & Xinaris, C. (2022). Thyroid hormone Signalling in human evolution and disease: A novel hypothesis. Journal of Clinical Medicine, 11(1), 43. 10.3390/jcm11010043 PMC8745179 35011782

[ajhb23811-bib-0273] Marie, C. , Ali, A. , Chandwe, K. , Petri, W. A. , & Kelly, P. (2018). Pathophysiology of environmental enteric dysfunction and its impact on oral vaccine efficacy. Mucosal Immunology, 11(5), 1290–1298. 29988114 10.1038/s41385-018-0036-1

[ajhb23811-bib-0274] Marty, P. R. , Van Noordwijk, M. A. , Heistermann, M. , Willems, E. P. , Dunkel, L. P. , Cadilek, M. , Agil, M. , & Weingrill, T. (2015). Endocrinological correlates of male bimaturism in wild Bornean orangutans. American Journal of Primatology, 77(11), 1170–1178. 26235914 10.1002/ajp.22453

[ajhb23811-bib-0275] Matsukura, T. , Kagamimori, S. , Nishino, H. , Yamagami, T. , Iki, M. , Kajita, E. , Kagawa, Y. , Yoneshima, H. , Matsuzaki, T. , & Marumo, F. (2003). The characteristics of bone turnover in the second decade in relation to age and puberty development in healthy Japanese male and female subjects‐Japanese population‐based osteoporosis study. Annals of Human Biology, 30(1), 13–25. 12519652 10.1080/03014460210157411

[ajhb23811-bib-0276] Matthews, A. M. , Kaur, H. , Dodd, M. , D'Souza, J. , Liloglou, T. , Shaw, R. J. , & Risk, J. M. (2013). Saliva collection methods for DNA biomarker analysis in oral cancer patients. British Journal of Oral and Maxillofacial Surgery, 51(5), 394–398. 23068125 10.1016/j.bjoms.2012.09.017

[ajhb23811-bib-0277] Mattison, J. A. , & Vaughan, K. L. (2017). An overview of nonhuman primates in aging research. Experimental Gerontology, 94, 41–45. 27956088 10.1016/j.exger.2016.12.005 PMC5466843

[ajhb23811-bib-0278] Mazur A , Booth A . 1997. Testosterone and dominance in men. 10097017

[ajhb23811-bib-0279] Mbuya, M. N. , & Humphrey, J. H. (2016). Preventing environmental enteric dysfunction through improved water, sanitation and hygiene: An opportunity for stunting reduction in developing countries. Maternal & Child Nutrition, 12, 106–120. 10.1111/mcn.12220 PMC5019251 26542185

[ajhb23811-bib-0280] McCabe G , Emery Thompson M , Ehardt C , Gillespie T . Trade‐offs between reproduction and parasitism in wild female Sanje mangabeys (*Cercocebus sanjei*); 2016.

[ajhb23811-bib-0281] McDade, T. , Burhop, J. , & Dohnal, J. (2004). High‐sensitivity enzyme immunoassay for C‐reactive protein in dried blood spots. Clinical Chemistry, 50(3), 650–652. 14981035 10.1373/clinchem.2003.029488

[ajhb23811-bib-0282] McDade, T. W. (2003). Life history theory and the immune system: Steps toward a human ecological immunology. American Journal of Physical Anthropology, 122(S37), 100–125. 10.1002/ajpa.10398 14666535

[ajhb23811-bib-0283] Mcdade, T. W. (2013). Development and validation of assay protocols for use with dried blood spot samples. American Journal of Human Biology, 26(1), 1–9. 24130128 10.1002/ajhb.22463

[ajhb23811-bib-0284] McDade, T. W. , Miller, A. , Tran, T. T. , Borders, A. E. , & Miller, G. (2021). A highly sensitive multiplex immunoassay for inflammatory cytokines in dried blood spots. American Journal of Human Biology, 33(6), e23558. 33382166 10.1002/ajhb.23558

[ajhb23811-bib-0285] McDade, T. W. , Rutherford, J. N. , Adair, L. , & Kuzawa, C. (2008). Adiposity and pathogen exposure predict C‐reactive protein in Filipino women. The Journal of Nutrition, 138(12), 2442–2447. 19022970 10.3945/jn.108.092700 PMC2801568

[ajhb23811-bib-0286] McDade, T. W. , Ryan, C. , Jones, M. J. , MacIsaac, J. L. , Morin, A. M. , Meyer, J. M. , Borja, J. B. , Miller, G. E. , Kobor, M. S. , & Kuzawa, C. W. (2017). Social and physical environments early in development predict DNA methylation of inflammatory genes in young adulthood. Proceedings of the National Academy of Sciences, 114(29), 7611–7616. 10.1073/pnas.1620661114 PMC5530653 28673994

[ajhb23811-bib-0287] McDade, T. W. , Ryan, C. P. , Jones, M. J. , Hoke, M. K. , Borja, J. , Miller, G. E. , Kuzawa, C. W. , & Kobor, M. S. (2019). Genome‐wide analysis of DNA methylation in relation to socioeconomic status during development and early adulthood. American Journal of Physical Anthropology, 169(1), 3–11. 30771258 10.1002/ajpa.23800

[ajhb23811-bib-0288] McDade, T. W. , Williams, S. , & Snodgrass, J. J. (2007a). What a drop can do: Dried blood spots as a minimally invasive method for integrating biomarkers into population‐based research. Demography, 44(4), 899–925. 18232218 10.1353/dem.2007.0038

[ajhb23811-bib-0289] McDade, T. W. , Williams, S. A. , & Snodgrass, J. J. (2007b). What a drop can do: Dried blood spots as a minimally invasive method for integrating biomarkers into population‐based research. Demography, 44(4), 899–925. 18232218 10.1353/dem.2007.0038

[ajhb23811-bib-0290] Mendonça‐Furtado, O. , Edaes, M. , Palme, R. , Rodrigues, A. , Siqueira, J. , & Izar, P. (2014). Does hierarchy stability influence testosterone and cortisol levels of bearded capuchin monkeys (*Sapajus libidinosus*) adult males? A comparison between two wild groups. Behavioural Processes, 109, 79–88. 25239540 10.1016/j.beproc.2014.09.010

[ajhb23811-bib-0291] Metzler, I. (2010). Biomarkers and their consequences for the biomedical profession: A social science perspective. Personalized Medicine, 7(4), 407–420. 29788645 10.2217/pme.10.41

[ajhb23811-bib-0292] Miller, A. A. , Sharrock, K. C. B. , & McDade, T. W. (2006). Measurement of leptin in dried blood spot samples. American Journal of Human Biology, 18(6), 857–860. 17039473 10.1002/ajhb.20566

[ajhb23811-bib-0293] Miller, E. M. , & McConnell, D. S. (2015). Milk immunity and reproductive status among Ariaal women of northern Kenya. Annals of Human Biology, 42(1), 76–83. 25154290 10.3109/03014460.2014.941398

[ajhb23811-bib-0294] Mondal, D. , Minak, J. , Alam, M. , Liu, Y. , Dai, J. , Korpe, P. , Liu, L. , Haque, R. , & Petri, W. A., Jr. (2012). Contribution of enteric infection, altered intestinal barrier function, and maternal malnutrition to infant malnutrition in Bangladesh. Clinical Infectious Diseases: An Official Publication of the Infectious Diseases Society of America, 54(2), 185–192. 22109945 10.1093/cid/cir807 PMC3245731

[ajhb23811-bib-0295] Muehlenbein, M. P. (2006). Intestinal parasite infections and fecal steroid levels in wild chimpanzees. American Journal of Physical Anthropology, 130(4), 546–550. 16444733 10.1002/ajpa.20391

[ajhb23811-bib-0296] Muehlenbein, M. P. , & Bribiescas, R. G. (2005). Testosterone‐mediated immune functions and male life histories. American Journal of Human Biology, 17(5), 527–558. 16136532 10.1002/ajhb.20419

[ajhb23811-bib-0297] Muehlenbein, M. P. , & Bribiescas, R. G. (2010). Male reproduction: Physiology, behavior, and ecology. In M. P. Muehlenbein (Ed.), Human Evolutionary Biology (pp. 351). Cambridge University Press.

[ajhb23811-bib-0298] Muehlenbein, M. P. , Prall, S. P. , & Nagao, P. H. (2017). Immunity, hormones, and life history trade‐offs. The Arc of Life (pp. 99–120). New York, NY: Springer.

[ajhb23811-bib-0299] Muehlenbein, M. P. , & Watts, D. P. (2010). The costs of dominance: Testosterone, cortisol and intestinal parasites in wild male chimpanzees. BioPsychoSocial Medicine, 4(1), 21. 21143892 10.1186/1751-0759-4-21 PMC3004803

[ajhb23811-bib-0300] Muehlenbein, M. P. , Watts, D. P. , & Whitten, P. L. (2004). Dominance rank and fecal testosterone levels in adult male chimpanzees (*Pan troglodytes* schweinfurthii) at Ngogo, Kibale National Park, Uganda. American Journal of Primatology, 64(1), 71–82. 15356859 10.1002/ajp.20062

[ajhb23811-bib-0301] Muletz‐Wolz, C. R. , Kurata, N. P. , Himschoot, E. A. , Wenker, E. S. , Quinn, E. A. , Hinde, K. , Power, M. L. , & Fleischer, R. C. (2019). Diversity and temporal dynamics of primate milk microbiomes. American Journal of Primatology, 81(10–11), e22994. 31219214 10.1002/ajp.22994 PMC6842035

[ajhb23811-bib-0302] Muller, M. , Marlowe, F. , Bugumba, R. , & Ellison, P. (2009). Testosterone and paternal care in east African foragers and pastoralists. Proceedings of the Royal Society B: Biological Sciences, 276(1655), 347–354. 10.1098/rspb.2008.1028 PMC2674347 18826936

[ajhb23811-bib-0303] Muller, M. N. (2017). Testosterone and reproductive effort in male primates. Hormones and Behavior, 91, 36–51. 27616559 10.1016/j.yhbeh.2016.09.001 PMC5342957

[ajhb23811-bib-0304] Muller, M. N. , & Wrangham, R. W. (2004a). Dominance, aggression and testosterone in wild chimpanzees: A test of the “challenge hypothesis”. Animal Behaviour, 67(1), 113–123.

[ajhb23811-bib-0305] Muller, M. N. , & Wrangham, R. W. (2004b). Dominance, cortisol and stress in wild chimpanzees (*Pan troglodytes schweinfurthii*). Behavioral Ecology and Sociobiology, 55(4), 332–340. 10.1007/s00265-020-02872-7 PMC7990237 33776193

[ajhb23811-bib-0306] Muller, M. N. , & Wrangham, R. W. (2004c). Dominance, cortisol and stress in wild chimpanzees (*Pan troglodytes schweinfurthii*). Behavioral Ecology and Sociobiology, 55(4), 332–340. 10.1007/s00265-020-02872-7 PMC7990237 33776193

[ajhb23811-bib-0307] Muller, M. N. , & Wrangham, R. W. (2005). Testosterone and energetics in wild chimpanzees (*Pan troglodytes schweinfurthii*). American Journal of Primatology: Official Journal of the American Society of Primatologists, 66(2), 119–130. 10.1002/ajp.20132 15940710

[ajhb23811-bib-0308] Mulligan, C. J. (2016). Early environments, stress, and the epigenetics of human health. Annual Review of Anthropology, 45(1), 233–249.

[ajhb23811-bib-0309] Mulligan, C. J. , Boyer, D. M. , Turner, T. R. , Delson, E. , & Leonard, W. R. (2022). Data sharing in biological anthropology. American Journal of Biological Anthropology, 178, 26–53.

[ajhb23811-bib-0310] Murray, C. M. , Heintz, M. R. , Lonsdorf, E. V. , Parr, L. A. , & Santymire, R. M. (2013). Validation of a field technique and characterization of fecal glucocorticoid metabolite analysis in wild chimpanzees (*Pan troglodytes*). American Journal of Primatology, 75(1), 57–64. 22968979 10.1002/ajp.22078 PMC3619224

[ajhb23811-bib-0311] Murtagh, R. , Behringer, V. , & Deschner, T. (2013). LC‐MS as a method for non‐invasive measurement of steroid hormones and their metabolites in urine and faeces of animals. Veterinary Medicine Austria, 100(9–10), 247–254.

[ajhb23811-bib-0312] Mustoe, A. C. , Taylor, J. H. , Birnie, A. K. , Huffman, M. C. , & French, J. A. (2014). Gestational cortisol and social play shape development of marmosets' HPA functioning and behavioral responses to stressors. Developmental Psychobiology, 56(6), 1229–1243. 24510474 10.1002/dev.21203 PMC5996393

[ajhb23811-bib-0313] Mwape, I. , Bosomprah, S. , Mwaba, J. , Mwila‐Kazimbaya, K. , Laban, N. , Chisenga, C. , Sijumbila, G. , Simuyandi, M. , & Chilengi, R. (2017). Immunogenicity of rotavirus vaccine (RotarixTM) in infants with environmental enteric dysfunction. PLoS One, 12, e0187761. 29281659 10.1371/journal.pone.0187761 PMC5744930

[ajhb23811-bib-0314] Narayan, E. J. (2013). Non‐invasive reproductive and stress endocrinology in amphibian conservation physiology. Conservation. Physiology, 1(1), cot011. 10.1093/conphys/cot011 PMC4806611 27293595

[ajhb23811-bib-0315] Naumenko, D. J. , Watford, M. , Atmoko, S. S. U. , Erb, W. M. , & Vogel, E. R. (2020). Evaluating ketosis in primate field studies: Validation of urine test strips in wild *Bornean orangutans* (*Pongo pygmaeus wurmbii*). Folia Primatologica, 91(2), 159–168. 10.1159/000501933 31536993

[ajhb23811-bib-0316] Nepomnaschy, P. A. , Welch, K. B. , McConnell, D. S. , Low, B. S. , Strassmann, B. I. , & England, B. G. (2006). Cortisol levels and very early pregnancy loss in humans. Proceedings of the National Academy of Sciences, 103(10), 3938–3942. 10.1073/pnas.0511183103 PMC1533790 16495411

[ajhb23811-bib-0317] Newton‐Fisher, N. E. (2017). Modeling social dominance: Elo‐ratings, prior history, and the intensity of aggression. International Journal of Primatology, 38(3), 427–447. 28680188 10.1007/s10764-017-9952-2 PMC5487812

[ajhb23811-bib-0318] Nicoloff, J. T. , Fisher, D. A. , & Appleman, M. D. (1970). The role of glucocorticoids in the regulation of thyroid function in man. The Journal of Clinical Investigation, 49(10), 1922–1929. 4990073 10.1172/JCI106411 PMC322682

[ajhb23811-bib-0319] Nicolson NA . 2008. Measurement of cortisol.

[ajhb23811-bib-0320] Nieschlag, E. , Behre, H. M. , & Nieschlag, S. (2012). Testosterone: Action, deficiency, substitution. Cambridge University Press.

[ajhb23811-bib-0321] Nieuwenhuizen, A. G. , & Rutters, F. (2008). The hypothalamic‐pituitary‐adrenal‐axis in the regulation of energy balance. Physiology & Behavior, 94(2), 169–177. 18275977 10.1016/j.physbeh.2007.12.011

[ajhb23811-bib-0322] Non, A. L. , & Thayer, Z. M. (2019). Epigenetics and human variation. In D. H. O'Rourke (Ed.), A companion to anthropological genetics (pp. 293–308). John Wiley & Sons.

[ajhb23811-bib-0323] Núñez‐de la Mora, A. , Chatterton, R. T. , Choudhury, O. A. , Napolitano, D. A. , & Bentley, G. R. (2007a). Childhood conditions influence adult progesterone levels. PLoS Medicine, 4(5), e167. 17503960 10.1371/journal.pmed.0040167 PMC1868040

[ajhb23811-bib-0324] Núñez‐de la Mora, A. , Chatterton, R. T. , Choudhury, O. A. , Napolitano, D. A. , & Bentley, G. R. (2007b). Childhood conditions influence adult progesterone levels. PLoS Medicine, 4(5), e167. 17503960 10.1371/journal.pmed.0040167 PMC1868040

[ajhb23811-bib-0325] Nunez‐de‐la‐Mora A , Bentley G . 2008. Early life effects on reproductive function. New perspectives on evolutionary medicine.

[ajhb23811-bib-0326] Nyberg, C. , Tanner, S. , TW, M. , & WR, L. (2010). Elevated diurnal cortisol rythms predict growth stunting among Tsimane' children. American Journal of Human Biology, 22, 265.

[ajhb23811-bib-0327] Nyberg, C. H. (2012). Diurnal cortisol rhythms in Tsimane'Amazonian foragers: New insights into ecological HPA axis research. Psychoneuroendocrinology, 37(2), 178–190. 21719201 10.1016/j.psyneuen.2011.06.002

[ajhb23811-bib-0328] Nyberg, C. H. , Leonard, W. R. , Tanner, S. , McDade, T. , Huanca, T. , & Godoy, R. A. (2012). Diurnal cortisol rhythms and child growth: Exploring the life history consequences of HPA activation among the Tsimane. American Journal of Human Biology, 24(6), 730–738. 23042663 10.1002/ajhb.22304

[ajhb23811-bib-0329] Onyango, P. O. , Gesquiere, L. R. , Altmann, J. , & Alberts, S. C. (2013). Puberty and dispersal in a wild primate population. Hormones and Behavior, 64(2), 240–249. 23998668 10.1016/j.yhbeh.2013.02.014 PMC3764504

[ajhb23811-bib-0330] Petrullo, L. , Hinde, K. , & Lu, A. (2019). Steroid hormone concentrations in milk predict sex‐specific offspring growth in a nonhuman primate. American Journal of Human Biology, 31(6), e23315. 31468643 10.1002/ajhb.23315

[ajhb23811-bib-0331] Petrullo, L. A. , Mandalaywala, T. M. , Parker, K. J. , Maestripieri, D. , & Higham, J. P. (2016). Effects of early life adversity on cortisol/salivary alpha‐amylase symmetry in free‐ranging juvenile rhesus macaques. Hormones and Behavior, 86, 78–84. 27170429 10.1016/j.yhbeh.2016.05.004 PMC6719785

[ajhb23811-bib-0332] Picó, C. , Serra, F. , Rodríguez, A. M. , Keijer, J. , & Palou, A. (2019). Biomarkers of nutrition and health: New tools for new approaches. Nutrients, 11(5), 1092. 31100942 10.3390/nu11051092 PMC6567133

[ajhb23811-bib-0333] Poisot, T. , Bruneau, A. , Gonzalez, A. , Gravel, D. , & Peres‐Neto, P. (2019). Ecological data should not be so hard to find and reuse. Trends in Ecology & Evolution, 34(6), 494–496. 31056219 10.1016/j.tree.2019.04.005

[ajhb23811-bib-0334] Pontzer, H. (2017). The crown joules: Energetics, ecology, and evolution in humans and other primates. Evolutionary Anthropology: Issues, News, and Reviews, 26(1), 12–24. 10.1002/evan.21513 28233387

[ajhb23811-bib-0335] Pontzer, H. , Raichlen, D. A. , Wood, B. M. , Emery Thompson, M. , Racette, S. B. , Mabulla, A. Z. , & Marlowe, F. W. (2015). Energy expenditure and activity among Hadza hunter‐gatherers. American Journal of Human Biology, 27(5), 628–637. 25824106 10.1002/ajhb.22711

[ajhb23811-bib-0336] Ponzi, D. , Flinn, M. V. , Muehlenbein, M. P. , & Nepomnaschy, P. A. (2020). Hormones and human developmental plasticity. Molecular and Cellular Endocrinology, 505, 110721. 32004677 10.1016/j.mce.2020.110721

[ajhb23811-bib-0337] Prall, S. P. , & Muehlenbein, M. P. (2014). Testosterone and immune function in primates: A brief summary with methodological considerations. International Journal of Primatology, 35(3), 805–824.

[ajhb23811-bib-0338] Preis, A. , Samuni, L. , Deschner, T. , Crockford, C. , & Wittig, R. M. (2019). Urinary cortisol, aggression, dominance and competition in wild, West African male chimpanzees. Frontiers in Ecology and Evolution, 7, 107.

[ajhb23811-bib-0339] Prendergast, A. J. , Rukobo, S. , Chasekwa, B. , Mutasa, K. , Ntozini, R. , Mbuya, M. N. N. , Jones, A. , Moulton, L. H. , Stoltzfus, R. J. , & Humphrey, J. H. (2014). Stunting is characterized by chronic inflammation in Zimbabwean infants. PLoS One, 9(2), e86928. 24558364 10.1371/journal.pone.0086928 PMC3928146

[ajhb23811-bib-0340] Pride, E. R. (2005). High faecal glucocorticoid levels predict mortality in ring‐tailed lemurs (*Lemur catta*). Biology Letters, 1(1), 60–63. 17148128 10.1098/rsbl.2004.0245 PMC1965197

[ajhb23811-bib-0341] Puterman, E. , Gemmill, A. , Karasek, D. , Weir, D. , Adler, N. E. , Prather, A. A. , & Epel, E. S. (2016). Lifespan adversity and later adulthood telomere length in the nationally representative US health and retirement study. Proceedings of the National Academy of Sciences, 113(42), E6335–E6342. 10.1073/pnas.1525602113 PMC5081642 27698131

[ajhb23811-bib-0342] Qin D‐D , Rizak JD , Feng X‐L , Chu X‐X , Yang S‐C , Li C‐L , Lv L‐B , Ma Y‐Y , Hu X‐T . 2013. Social rank and cortisol among female rhesus macaques (*Macaca mulatta*). 10.3724/SP.J.1141.2013.E02E42 23572366

[ajhb23811-bib-0343] Quinn, E. A. (2021). Centering human milk composition as normal human biological variation. American Journal of Human Biology, 33(1), e23564. 33432701 10.1002/ajhb.23564

[ajhb23811-bib-0344] Raichlen, D. A. , Pontzer, H. , Harris, J. A. , Mabulla, A. Z. , Marlowe, F. W. , Josh Snodgrass, J. , Eick, G. , Colette Berbesque, J. , Sancilio, A. , & Wood, B. M. (2017). Physical activity patterns and biomarkers of cardiovascular disease risk in hunter‐gatherers. American Journal of Human Biology, 29(2), e22919. 10.1002/ajhb.22919 27723159

[ajhb23811-bib-0345] Rakotoniaina, J. H. , Kappeler, P. M. , Kaesler, E. , Hämäläinen, A. M. , Kirschbaum, C. , & Kraus, C. (2017). Hair cortisol concentrations correlate negatively with survival in a wild primate population. BMC Ecology, 17(1), 1–13. 28859635 10.1186/s12898-017-0140-1 PMC5579956

[ajhb23811-bib-0502] Rangel‐Negrín, A. ., Alfaro, J. L., Valdez, R. A., Romano, M. C., & Serio‐Silva, J. C. (2009). Stress in Yucatan spider monkeys: effects of environmental conditions on fecal cortisol levels in wild and captive populations. Animal Conservation, 12(5), 496–502.

[ajhb23811-bib-0346] Rangel‐Negrin, A. , Coyohua Fuentes, A. , de la Torre, H. A. , Cano Huertes, B. , Reynoso Cruz, E. , Ceccarelli, E. , Gomez Espinosa, E. E. , Chavira Ramirez, D. R. , Moreno Espinoza, D. E. , & Canales‐Espinosa, D. (2021). Female reproductive energetics in mantled howler monkeys (*Alouatta palliata*): A follow‐up study. American Journal of Physical Anthropology, 174(3), 396–406. 33429455 10.1002/ajpa.24222

[ajhb23811-bib-0347] Rauchenzauner, M. , Schmid, A. , Heinz‐Erian, P. , Kapelari, K. , Falkensammer, G. , Griesmacher, A. , Finkenstedt, G. , & Hogler, W. (2007). Sex‐and age‐specific reference curves for serum markers of bone turnover in healthy children from 2 months to 18 years. The Journal of Clinical Endocrinology & Metabolism, 92(2), 443–449. 17105843 10.1210/jc.2006-1706

[ajhb23811-bib-0348] Reiches, M. W. , Moore, S. E. , Prentice, A. M. , & Ellison, P. T. (2014). Endocrine responses, weight change, and energy sparing mechanisms during Ramadan among Gambian adolescent women. American Journal of Human Biology, 26(3), 395–400. 24590590 10.1002/ajhb.22531

[ajhb23811-bib-0349] Rej, P. H. , Bondy, M. H. , Lin, J. , Prather, A. A. , Kohrt, B. A. , Worthman, C. M. , & Eisenberg, D. T. A. (2021). Telomere length analysis from minimally‐invasively collected samples: Methods development and meta‐analysis of the validity of different sampling techniques. American Journal of Human Biology, 33(1), e23410. 32189404 10.1002/ajhb.23410 PMC8105084

[ajhb23811-bib-0350] Rej, P. H. , Committee, H. S. , Gravlee, C. C. , & Mulligan, C. J. (2020). Shortened telomere length is associated with unfair treatment attributed to race in African Americans living in Tallahassee. Florida. American Journal of Human Biology, 32(3), e23375. 31867825 10.1002/ajhb.23375

[ajhb23811-bib-0351] Ricklefs, R. E. , & Wikelski, M. (2002). The physiology/life‐history nexus. Trends in Ecology & Evolution, 17(10), 462–468.

[ajhb23811-bib-0352] Robbins, M. M. , & Czekala, N. M. (1997). A preliminary investigation of urinary testosterone and cortisol levels in wild male mountain gorillas. American Journal of Primatology, 43(1), 51–64. 9294641 10.1002/(SICI)1098-2345(1997)43:1<51::AID-AJP4>3.0.CO;2-X

[ajhb23811-bib-0353] Roberts, D. F. (1952). Basal metabolism, race and climate. The Journal of the Royal Anthropological Institute of Great Britain and Ireland, 82(2), 169–183.

[ajhb23811-bib-0354] Röjdmark, S. (1987). Influence of short‐term fasting on the pituitary‐testicular axis in normal men. Hormone Research in Pædiatrics, 25(3), 140–146. 10.1159/000180645 3106181

[ajhb23811-bib-0355] Rosen, H. , Moses, A. , Garber, J. , Iloputaife, I. , Ross, D. , Lee, S. , & Greenspan, S. (2000). Serum CTX: A new marker of bone resorption that shows treatment effect more often than other markers because of low coefficient of variability and large changes with bisphosphonate therapy. Calcified Tissue International, 66(2), 100–103. 10652955 10.1007/pl00005830

[ajhb23811-bib-0356] Rosenbaum, S. , Gettler, L. T. , McDade, T. W. , Belarmino, N. M. , & Kuzawa, C. W. (2018). The effects of collection and storage conditions in the field on salivary testosterone, cortisol, and sIgA values. Annals of Human Biology, 45(5), 428–434. 30326745 10.1080/03014460.2018.1495263

[ajhb23811-bib-0357] Rosner, W. , Auchus, R. J. , Azziz, R. , Sluss, P. M. , & Raff, H. (2007). Utility, limitations, and pitfalls in measuring testosterone: An Endocrine Society position statement. The Journal of Clinical Endocrinology & Metabolism, 92(2), 405–413. 17090633 10.1210/jc.2006-1864

[ajhb23811-bib-0358] Ross, C. N. , & French, J. A. (2011). Female marmosets' behavioral and hormonal responses to unfamiliar intruders. American Journal of Primatology, 73(10), 1072–1081. 21748772 10.1002/ajp.20975 PMC6000820

[ajhb23811-bib-0359] Sacco, A. J. , Granatosky, M. C. , Laird, M. F. , & Milich, K. M. (2021). Validation of a method for quantifying urinary C‐peptide in platyrrhine monkeys. General and Comparative Endocrinology, 300, 113644. 33045233 10.1016/j.ygcen.2020.113644

[ajhb23811-bib-0360] Sacco, A. J. , Mayhew, J. A. , Watsa, M. , Erkenswick, G. , & Binder, A. K. (2020). Detection of neopterin in the urine of captive and wild platyrrhines. BMC Zoology, 5(1), 2.

[ajhb23811-bib-0361] Sapolsky, R. M. (2005). The influence of social hierarchy on primate health. Science, 308(5722), 648–652. 15860617 10.1126/science.1106477

[ajhb23811-bib-0362] Sapolsky, R. M. , Romero, L. M. , & Munck, A. U. (2000a). How do glucocorticoids influence stress responses? Integrating permissive, suppressive, stimulatory, and preparative actions. Endocrine Reviews, 21(1), 55–89. 10696570 10.1210/edrv.21.1.0389

[ajhb23811-bib-0363] Sapolsky, R. M. , Romero, L. M. , & Munck, A. U. (2000b). How do glucocorticoids influence stress responses? Integrating permissive, suppressive, stimulatory, and preparative actions. Endocrine Reviews, 21(1), 55–89. 10696570 10.1210/edrv.21.1.0389

[ajhb23811-bib-0364] Saxbe, D. E. (2008). A field (researcher's) guide to cortisol: Tracking HPA axis functioning in everyday life. Health Psychology Review, 2(2), 163–190.

[ajhb23811-bib-0365] Schaebs, F. S. , Wolf, T. E. , Behringer, V. , & Deschner, T. (2016). Fecal thyroid hormones allow for the noninvasive monitoring of energy intake in capuchin monkeys. Journal of Endocrinology, 231(1), 1–10. 27460343 10.1530/JOE-16-0152

[ajhb23811-bib-0366] Schoof, V. A. , Jack, K. M. , & Ziegler, T. E. (2014). Male response to female ovulation in white‐faced capuchins (*Cebus capucinus*): Variation in fecal testosterone, dihydrotestosterone, and glucocorticoids. International Journal of Primatology, 35(3), 643–660.

[ajhb23811-bib-0367] Schoof, V. A. M. , Jack, K. M. , & Carnegie, S. D. (2012). Rise to power: A case study of male fecal androgen and cortisol levels before and after a non‐aggressive rank change in a group of wild white‐faced Capuchins (*Cebus capucinus*). Folia Primatologica, 82(6), 299–307. 10.1159/000337220 22488354

[ajhb23811-bib-0368] Schrock, A. E. , Leard, C. , Lutz, M. C. , Meyer, J. S. , & Gazes, R. P. (2019). Aggression and social support predict long‐term cortisol levels in captive tufted capuchin monkeys (*Cebus* [*Sapajus*] *apella*). American Journal of Primatology, 81(7), e23001. 31180152 10.1002/ajp.23001

[ajhb23811-bib-0369] Schurmeyer T , Nieschlag E . Salivary and serum testosterone under physiological and pharmacological conditions; 1982. p 209.

[ajhb23811-bib-0370] Seethaler, B. , Basrai, M. , Neyrinck, A. M. , Nazare, J.‐A. , Walter, J. , Delzenne, N. M. , & Bischoff, S. C. (2021). Biomarkers for assessment of intestinal permeability in clinical practice. American journal of physiology‐gastrointestinal and liver. Physiology, 321(1), G11–G17. 10.1152/ajpgi.00113.2021 34009040

[ajhb23811-bib-0371] Shakespear, R. A. , & Burke, C. W. (1976). Triiodothyronine and thyroxine in urine. I. Measurement and application. The Journal of Clinical Endocrinology & Metabolism, 42(3), 494–503. 1254690 10.1210/jcem-42-3-494

[ajhb23811-bib-0372] Sharrock, K. C. B. , Kuzawa, C. W. , Leonard, W. R. , Tanner, S. , Reyes‐García, V. E. , Vadez, V. , Huanca, T. , & McDade, T. W. (2008). Developmental changes in the relationship between leptin and adiposity among Tsimané children and adolescents. American Journal of Human Biology, 20(4), 392–398. 18348174 10.1002/ajhb.20765

[ajhb23811-bib-0373] Shattuck, E. C. , & Muehlenbein, M. P. (2015). Human sickness behavior: Ultimate and proximate explanations. American Journal of Physical Anthropology, 157(1), 1–18. 25639499 10.1002/ajpa.22698

[ajhb23811-bib-0508] Shetty, S. , Kapoor, N., Bondu, J. D., Thomas, N., & Paul, T. V. (2016). Bone turnover markers: Emerging tool in the management of osteoporosis. Indian Journal of Endocrinology and Metabolism, 20(6), 846–852. 27867890 10.4103/2230-8210.192914 PMC5105571

[ajhb23811-bib-0374] Sherman, G. D. , & Mehta, P. H. (2020). Stress, cortisol, and social hierarchy. Current Opinion in Psychology, 33, 227–232. 31765930 10.1016/j.copsyc.2019.09.013

[ajhb23811-bib-0375] Sherry, D. , & Ellison, P. (2007a). Potential applications of urinary C‐peptide of insulin for comparative energetics research. American Journal of Physical Anthropology, 133(1), 771–778. 17295295 10.1002/ajpa.20562

[ajhb23811-bib-0376] Sherry, D. S. , & Ellison, P. T. (2007b). Potential applications of urinary C‐peptide of insulin for comparative energetics research. American Journal of Physical Anthropology, 133(1), 771–778. 17295295 10.1002/ajpa.20562

[ajhb23811-bib-0377] Singer, B. D. (2019). A practical guide to the measurement and analysis of DNA methylation. American Journal of Respiratory Cell and Molecular Biology, 61(4), 417–428. 31264905 10.1165/rcmb.2019-0150TR PMC6775954

[ajhb23811-bib-0378] Sousa, M. B. C. , & Ziegler, T. E. (1998). Diurnal variation on the excretion patterns of fecal steroids in common marmoset (*Callithrix jacchus*) females. American Journal of Primatology, 46(2), 105–117. 9773674 10.1002/(SICI)1098-2345(1998)46:2<105::AID-AJP1>3.0.CO;2-#

[ajhb23811-bib-0379] Stearns, S. C. (1992). The evolution of life histories. Oxford University Press.

[ajhb23811-bib-0380] Steinert, S. , White, D. M. , Zou, Y. , Shay, J. W. , & Wright, W. E. (2002). Telomere biology and cellular aging in nonhuman primate cells. Experimental Cell Research, 272(2), 146–152. 11777339 10.1006/excr.2001.5409

[ajhb23811-bib-0381] Stout, S. A. , Lin, J. , Hernandez, N. , Davis, E. P. , Blackburn, E. , Carroll, J. E. , & Glynn, L. M. (2017). Validation of minimally‐invasive sample collection methods for measurement of telomere length. Frontiers in Aging Neuroscience, 9, 1–6. 29270121 10.3389/fnagi.2017.00397 PMC5723637

[ajhb23811-bib-0382] Stroup, G. B. , Lark, M. W. , Veber, D. F. , Bhattacharyya, A. , Blake, S. , Dare, L. C. , Erhard, K. F. , Hoffman, S. J. , James, I. E. , & Marquis, R. W. (2001). Potent and selective inhibition of human cathepsin K leads to inhibition of bone resorption in vivo in a nonhuman primate. Journal of Bone and Mineral Research, 16(10), 1739–1746. 11585335 10.1359/jbmr.2001.16.10.1739

[ajhb23811-bib-0383] Sullivan, E. C. , Hinde, K. , Mendoza, S. P. , & Capitanio, J. P. (2011). Cortisol concentrations in the milk of rhesus monkey mothers are associated with confident temperament in sons, but not daughters. Developmental Psychobiology, 53(1), 96–104. 20730788 10.1002/dev.20483 PMC3188439

[ajhb23811-bib-0384] Summers, K. , Harrison, G. , Hume, D. , & Palmer, C. (1983). Urinary hormone levels: A population study of associations between steroid and catecholamine excretion rates. Annals of Human Biology, 10(2), 99–110. 6682303 10.1080/03014468300006241

[ajhb23811-bib-0385] Surbeck, M. , Deschner, T. , Behringer, V. , & Hohmann, G. (2015). Urinary C‐peptide levels in male bonobos (*Pan paniscus*) are related to party size and rank but not to mate competition. Hormones and Behavior, 71, 22–30. 25870021 10.1016/j.yhbeh.2015.03.007

[ajhb23811-bib-0386] Surbeck, M. , Deschner, T. , Weltring, A. , & Hohmann, G. (2012). Social correlates of variation in urinary cortisol in wild male bonobos (*Pan paniscus*). Hormones and Behavior, 62(1), 27–35. 22565126 10.1016/j.yhbeh.2012.04.013

[ajhb23811-bib-0387] Szulc, P. , Seeman, E. , & Delmas, P. (2000). Biochemical measurements of bone turnover in children and adolescents. Osteoporosis International, 11(4), 281–294. 10928217 10.1007/s001980070116

[ajhb23811-bib-0388] Tanner, J. , Wilson, M. , & Rudman, C. (1990). Pubertal growth spurt in the female rhesus monkey: Relation to menarche and skeletal maturation. American Journal of Human Biology, 2(2), 101–106. 28590532 10.1002/ajhb.1310020202

[ajhb23811-bib-0389] Taraborrelli, S. (2015). Physiology, production and action of progesterone. Acta Obstetricia et Gynecologica Scandinavica, 94, 8–16. 10.1111/aogs.12771 26358238

[ajhb23811-bib-0390] Thayer, Z. M. , Feranil, A. B. , & Kuzawa, C. W. (2012). Maternal cortisol disproportionately impacts fetal growth in male offspring: Evidence from The Philippines. American Journal of Human Biology, 24(1), 1–4. 22121049 10.1002/ajhb.21226 PMC4181846

[ajhb23811-bib-0391] Thayer, Z. M. , & Kuzawa, C. W. (2011). Biological memories of past environments: Epigenetic pathways to health disparities. Epigenetics, 6(7), 798–803. 21597338 10.4161/epi.6.7.16222

[ajhb23811-bib-0392] Thompson, A. L. , Houck, K. M. , & Jahnke, J. R. (2019). Pathways linking caesarean delivery to early health in a dual burden context: Immune development and the gut microbiome in infants and children from Galápagos, Ecuador. American Journal of Human Biology: The Official Journal of the Human Biology Council, 31, e23219. 10.1002/ajhb.23219 PMC6661198 30693586

[ajhb23811-bib-0393] Thompson, A. L. , & Lampl, M. (2013). Prenatal and postnatal energetic conditions and sex steroids levels across the first year of life. American Journal of Human Biology, 25(5), 643–654. 23904043 10.1002/ajhb.22424 PMC4271319

[ajhb23811-bib-0394] Thompson, C. L. , Powell, B. L. , Williams, S. H. , Hanya, G. , Glander, K. E. , & Vinyard, C. J. (2017). Thyroid hormone fluctuations indicate a thermoregulatory function in both a tropical (*Alouatta palliata*) and seasonally cold‐habitat (*Macaca fuscata*) primate. American Journal of Primatology, 79(11), e22714. 10.1002/ajp.22714 29048740

[ajhb23811-bib-0395] Thuijls, G. , Derikx, J. P. , de Haan, J.‐J. , Grootjans, J. , de Bruïne, A. , Masclee, A. A. , Heineman, E. , & Buurman, W. A. (2010). Urine‐based detection of intestinal tight junction loss. Journal of Clinical Gastroenterology, 44(1), e14–e19. 19525861 10.1097/MCG.0b013e31819f5652

[ajhb23811-bib-0396] Tickell, K. D. , Atlas, H. E. , & Walson, J. L. (2019). Environmental enteric dysfunction: A review of potential mechanisms, consequences and management strategies. BMC Medicine, 17(1), 181. 31760941 10.1186/s12916-019-1417-3 PMC6876067

[ajhb23811-bib-0397] Tkaczynski, P. J. , Behringer, V. , Ackermann, C. Y. , Fedurek, P. , Fruth, B. , Girard‐Buttoz, C. , Hobaiter, C. , Lee, S. M. , Löhrich, T. , & Preis, A. (2020). Patterns of urinary cortisol levels during ontogeny appear population specific rather than species specific in wild chimpanzees and bonobos. Journal of Human Evolution, 147, 102869. 32866765 10.1016/j.jhevol.2020.102869

[ajhb23811-bib-0398] Touma, C. , & Palme, R. (2005). Measuring fecal glucocorticoid metabolites in mammals and birds: The importance of validation. Annals of the New York Academy of Sciences, 1046(1), 54–74. 16055843 10.1196/annals.1343.006

[ajhb23811-bib-0399] Trost, L. W. , & Mulhall, J. P. (2016). Challenges in testosterone measurement, data interpretation, and methodological appraisal of interventional trials. The Journal of Sexual Medicine, 13(7), 1029–1046. 27209182 10.1016/j.jsxm.2016.04.068 PMC5516925

[ajhb23811-bib-0400] Trumble, B. C. , Brindle, E. , Kupsik, M. , & O'Connor, K. A. (2010). Responsiveness of the reproductive axis to a single missed evening meal in young adult males. American Journal of Human Biology, 22(6), 775–781. 20721980 10.1002/ajhb.21079 PMC3111063

[ajhb23811-bib-0401] Trumble, B. C. , Cummings, D. , von Rueden, C. , O'Connor, K. A. , Smith, E. A. , Gurven, M. , & Kaplan, H. (2012). Physical competition increases testosterone among Amazonian forager‐horticulturalists: A test of the “challenge hypothesis”. Proceedings of the Royal Society B: Biological Sciences, 279(1739), 2907–2912. 10.1098/rspb.2012.0455 PMC3367794 22456888

[ajhb23811-bib-0402] Trumble, B. C. , Cummings, D. K. , O'Connor, K. A. , Holman, D. J. , Smith, E. A. , Kaplan, H. S. , & Gurven, M. D. (2013). Age‐independent increases in male salivary testosterone during horticultural activity among Tsimane forager‐farmers. Evolution and Human Behavior, 34(5), 350–357. 10.1016/j.evolhumbehav.2013.06.002 PMC3810999 24187482

[ajhb23811-bib-0403] Tung, J. , Barreiro, L. B. , Burns, M. B. , Grenier, J.‐C. , Lynch, J. , Grieneisen, L. E. , Altmann, J. , Alberts, S. C. , Blekhman, R. , & Archie, E. A. (2015). Social networks predict gut microbiome composition in wild baboons. eLife, 4, e05224. 25774601 10.7554/eLife.05224 PMC4379495

[ajhb23811-bib-0404] Tung, J. , Barreiro, L. B. , Johnson, Z. P. , Hansen, K. D. , Michopoulos, V. , Toufexis, D. , Michelini, K. , Wilson, M. E. , & Gilad, Y. (2012). Social environment is associated with gene regulatory variation in the rhesus macaque immune system. Proceedings of the National Academy of Sciences, 109(17), 6490–6495. 10.1073/pnas.1202734109 PMC3340061 22493251

[ajhb23811-bib-0405] Uddin, M. I. , Hossain, M. , Islam, S. , Akter, A. , Nishat, N. S. , Nila, T. A. , Rafique, T. A. , Leung, D. T. , Calderwood, S. B. , & Ryan, E. T. (2021). An assessment of potential biomarkers of environment enteropathy and its association with age and microbial infections among children in Bangladesh. PLoS One, 16(4), e0250446. 33886672 10.1371/journal.pone.0250446 PMC8061931

[ajhb23811-bib-0406] Urlacher, S. S. , Blackwell, A. D. , Liebert, M. A. , Madimenos, F. C. , Cepon‐Robins, T. J. , Gildner, T. E. , Snodgrass, J. J. , & Sugiyama, L. S. (2016). Physical growth of the Shuar: Height, weight, and BMI references for an indigenous Amazonian population. American Journal of Human Biology, 28(1), 16–30. 26126793 10.1002/ajhb.22747 PMC4696921

[ajhb23811-bib-0407] Urlacher, S. S. , Ellison, P. T. , Sugiyama, L. S. , Pontzer, H. , Eick, G. , Liebert, M. A. , Cepon‐Robins, T. J. , Gildner, T. E. , & Snodgrass, J. J. (2018). Tradeoffs between immune function and childhood growth among Amazonian forager‐horticulturalists. Proceedings of the National Academy of Sciences, 115(17), E3914–E3921. 10.1073/pnas.1717522115 PMC5924892 29632170

[ajhb23811-bib-0408] Urlacher, S. S. , & Kramer, K. L. (2018). Evidence for energetic tradeoffs between physical activity and childhood growth across the nutritional transition. Scientific Reports, 8(369), 1–10. 29321690 10.1038/s41598-017-18738-4 PMC5762677

[ajhb23811-bib-0409] Urlacher, S. S. , Liebert, M. A. , Josh Snodgrass, J. , Blackwell, A. D. , Cepon‐Robins, T. J. , Gildner, T. E. , Madimenos, F. C. , Amir, D. , Bribiescas, R. G. , & Sugiyama, L. S. (2016). Heterogeneous effects of market integration on sub‐adult body size and nutritional status among the Shuar of Amazonian Ecuador. Annals of Human Biology, 43(4), 316–329. 27230632 10.1080/03014460.2016.1192219 PMC4992548

[ajhb23811-bib-0410] Urlacher, S. S. , Liebert, M. A. , & Konečná, M. (2018b). Global variation in diurnal cortisol rhythms: Evidence from Garisakang forager‐horticulturalists of lowland Papua New Guinea. Stress, 21(2), 101–109. 29237322 10.1080/10253890.2017.1414798

[ajhb23811-bib-0411] Urlacher, S. S. , Liebert, M. A. , & Konečná, M. (2018c). Global variation in diurnal cortisol rhythms: Evidence from Garisakang forager‐horticulturalists of lowland Papua New Guinea. Stress, 21(2), 101–109. 29237322 10.1080/10253890.2017.1414798

[ajhb23811-bib-0412] Urlacher, S. S. , Snodgrass, J. J. , Dugas, L. R. , Madimenos, F. C. , Sugiyama, L. S. , Liebert, M. A. , Joyce, C. J. , Terán, E. , & Pontzer, H. (2021a). Childhood daily energy expenditure does not decrease with market integration and is not related to adiposity in Amazonia. The Journal of Nutrition, 195(3), 695–704. 10.1093/jn/nxaa361 33454748

[ajhb23811-bib-0413] Urlacher, S. S. , Snodgrass, J. J. , Dugas, L. R. , Madimenos, F. C. , Sugiyama, L. S. , Liebert, M. A. , Joyce, C. J. , Terán, E. , & Pontzer, H. (2021b). Childhood daily energy expenditure does not decrease with market integration and is not related to adiposity in Amazonia. The Journal of Nutrition, 151(3), 695–704. 33454748 10.1093/jn/nxaa361

[ajhb23811-bib-0414] Urlacher, S. S. , Snodgrass, J. J. , Dugas, L. R. , Sugiyama, L. S. , Liebert, M. A. , Joyce, C. J. , & Pontzer, H. (2019a). Constraint and trade‐offs regulate energy expenditure during childhood. Science Advances, 5(12), eaax1065. 32064311 10.1126/sciadv.aax1065 PMC6989306

[ajhb23811-bib-0415] Urlacher, S. S. , Snodgrass, J. J. , Dugas, L. R. , Sugiyama, L. S. , Liebert, M. A. , Joyce, C. J. , & Pontzer, H. (2019b). Constraint and trade‐offs regulate energy expenditure during childhood. Science Advances, 5(12), eaax1065. 32064311 10.1126/sciadv.aax1065 PMC6989306

[ajhb23811-bib-0416] Valeggia, C. , & Ellison, P. T. (2004). Lactational amenorrhoea in well‐nourished Toba women of Formosa, Argentina. Journal of Biosocial Science, 36(5), 573–595. 15446353 10.1017/s0021932003006382

[ajhb23811-bib-0417] Valeggia, C. , & Ellison, P. T. (2009a). Interactions between metabolic and reproductive functions in the resumption of postpartum fecundity. American Journal of Human Biology: The Official Journal of the Human Biology Council, 21(4), 559–566. 19298003 10.1002/ajhb.20907 PMC3305908

[ajhb23811-bib-0418] Valeggia, C. , & Ellison, P. T. (2009b). Interactions between metabolic and reproductive functions in the resumption of postpartum fecundity. American Journal of Human Biology, 21(4), 559–566. 19298003 10.1002/ajhb.20907 PMC3305908

[ajhb23811-bib-0419] Valeggia, C. R. (2007). Taking the lab to the field: Monitoring reproductive hormones in population research. Population and Development Review, 33(3), 525–542.

[ajhb23811-bib-0420] Valeggia, C. R. , & Ellison, P. T. (2001). Lactation, energetics, and postpartum fecundity. In P. T. Ellison (Ed.), Reproductive ecology and human evolution (pp. 85–105). Hawthorne, New York: Aldine De Gruyter.

[ajhb23811-bib-0421] van Anders, S. M. , Goldey, K. L. , & Bell, S. N. (2014). Measurement of testosterone in human sexuality research: Methodological considerations. Archives of Sexual Behavior, 43(2), 231–250. 23807216 10.1007/s10508-013-0123-z

[ajhb23811-bib-0422] Vandeleest, J. J. , Winkler, S. L. , Beisner, B. A. , Hannibal, D. L. , Atwill, E. R. , & McCowan, B. (2020). Sex differences in the impact of social status on hair cortisol concentrations in rhesus monkeys (*Macaca mulatta*). American Journal of Primatology, 82(1), e23086. 31876328 10.1002/ajp.23086 PMC6980377

[ajhb23811-bib-0423] Vitzthum, V. J. , Bentley, G. R. , Spielvogel, H. , Caceres, E. , Thornburg, J. , Jones, L. , Shore, S. , Hodges, K. R. , & Chatterton, R. T. (2002). Salivary progesterone levels and rate of ovulation are significantly lower in poorer than in better‐off urban‐dwelling Bolivian women. Human Reproduction, 17(7), 1906–1913. 12093859 10.1093/humrep/17.7.1906

[ajhb23811-bib-0424] Vitzthum, V. J. , Spielvogel, H. , & Thornburg, J. (2004). Interpopulational differences in progesterone levels during conception and implantation in humans. Proceedings of the National Academy of Sciences, 101(6), 1443–1448. 10.1073/pnas.0302640101 PMC341739 14757831

[ajhb23811-bib-0425] Vitzthum, V. J. , Worthman, C. M. , Beall, C. M. , Thornburg, J. , Vargas, E. , Villena, M. , Soria, R. , Caceres, E. , & Spielvogel, H. (2009). Seasonal and circadian variation in salivary testosterone in rural Bolivian men. American Journal of Human Biology: The Official Journal of the Human Biology Association, 21(6), 762–768. 10.1002/ajhb.20927 PMC3771338 19367574

[ajhb23811-bib-0426] Vogel, E. R. , Crowley, B. E. , Knott, C. D. , Blakely, M. D. , Larsen, M. D. , & Dominy, N. J. (2012). A noninvasive method for estimating nitrogen balance in free‐ranging primates. International Journal of Primatology, 33(3), 567–587.

[ajhb23811-bib-0427] Von Rueden, C. R. , Trumble, B. C. , Emery Thompson, M. , Stieglitz, J. , Hooper, P. L. , Blackwell, A. D. , Kaplan, H. S. , & Gurven, M. (2014). Political influence associates with cortisol and health among egalitarian forager‐farmers. Evolution, Medicine, and Public Health, 2014(1), 122–133. 25214482 10.1093/emph/eou021 PMC4178369

[ajhb23811-bib-0428] Wang, C. Y. , & Maibach, H. I. (2011). Why minimally invasive skin sampling techniques? A bright scientific future. Cutaneous and Ocular Toxicology, 30(1), 1–6. 20883150 10.3109/15569527.2010.517230

[ajhb23811-bib-0429] Wang, E. , Cho, W. C. , Wong, S. C. , & Liu, S. (2017). Disease biomarkers for precision medicine: Challenges and future opportunities. Genomics, Proteomics & Bioinformatics, 15(2), 57–58. 10.1016/j.gpb.2017.04.001 PMC5414969 28392478

[ajhb23811-bib-0430] Wasser, S. K. , Azkarate, J. C. , Booth, R. K. , Hayward, L. , Hunt, K. , Ayres, K. , Vynne, C. , Gobush, K. , Canales‐Espinosa, D. , & Rodríguez‐Luna, E. (2010). Non‐invasive measurement of thyroid hormone in feces of a diverse array of avian and mammalian species. General and Comparative Endocrinology, 168(1), 1–7. 20412809 10.1016/j.ygcen.2010.04.004

[ajhb23811-bib-0431] Weingrill, T. , Gray, D. A. , Barrett, L. , & Henzi, S. P. (2004). Fecal cortisol levels in free‐ranging female chacma baboons: Relationship to dominance, reproductive state and environmental factors. Hormones and Behavior, 45(4), 259–269. 15053942 10.1016/j.yhbeh.2003.12.004

[ajhb23811-bib-0432] Weltring, A. , Schaebs, F. S. , Perry, S. E. , & Deschner, T. (2012). Simultaneous measurement of endogenous steroid hormones and their metabolites with LC–MS/MS in faeces of a new world primate species, *Cebus capucinus* . Physiology & Behavior, 105(2), 510–521. 21945370 10.1016/j.physbeh.2011.09.004

[ajhb23811-bib-0433] West‐Eberhard, M. J. (2003a). Developmental plasticity and evolution. Oxford University Press.

[ajhb23811-bib-0434] West‐Eberhard, M. J. (2003b). Developmental plasticity and evolution (p. 815). Oxford University Press.

[ajhb23811-bib-0435] Williams, G. C. (2008). Adaptation and natural selection: A critique of some current evolutionary thought. Princeton University Press.

[ajhb23811-bib-0436] Wingfield, J. C. (2017). The challenge hypothesis: Where it began and relevance to humans. Hormones and Behavior, 92, 9–12. 27856292 10.1016/j.yhbeh.2016.11.008

[ajhb23811-bib-0437] Wingfield, J. C. , Hegner, R. E. , Dufty, A. M. , & Ball, G. F. (1990). The “challenge hypothesis”: Theoretical implications for patterns of testosterone secretion, mating systems, and breeding strategies. The American Naturalist, 136(6), 829–846.

[ajhb23811-bib-0438] Wittig, R. M. , Crockford, C. , Deschner, T. , Langergraber, K. E. , Ziegler, T. E. , & Zuberbühler, K. (2014). Food sharing is linked to urinary oxytocin levels and bonding in related and unrelated wild chimpanzees. Proceedings of the Royal Society B: Biological Sciences, 281(1778), 20133096. 10.1098/rspb.2013.3096 PMC3906952 24430853

[ajhb23811-bib-0439] Wobber, V. , Hare, B. , Maboto, J. , Lipson, S. , Wrangham, R. , & Ellison, P. T. (2010). Differential changes in steroid hormones before competition in bonobos and chimpanzees. Proceedings of the National Academy of Sciences, 107(28), 12457–12462. 10.1073/pnas.1007411107 PMC2906573 20616027

[ajhb23811-bib-0440] Worthman, C. M. , & Stallings, J. F. (1997). Hormone measures in finger‐prick blood spot samples: New field methods for reproductive endocrinology. American Journal of Physical Anthropology, 104(1), 1–21. 9331450 10.1002/(SICI)1096-8644(199709)104:1<1::AID-AJPA1>3.0.CO;2-V

[ajhb23811-bib-0441] Wu, X. , Xu, X. , Liu, Q. , Ding, J. , Liu, J. , Huang, Z. , Huang, Z. , Wu, X. , Li, R. , & Yang, Z. (2021). Unilateral cervical spinal cord injury induces bone loss and metabolic changes in non‐human primates (*Macaca fascicularis*). Journal of orthopaedic translation, 29, 113–122. 34178602 10.1016/j.jot.2021.03.006 PMC8193057

[ajhb23811-bib-0442] Xia, D.‐P. , Wang, X. , Zhang, Q.‐X. , Sun, B.‐H. , Sun, L. , Sheeran, L. K. , & Li, J.‐H. (2018). Progesterone levels in seasonally breeding, free‐ranging male *Macaca thibetana* . Mammal Research, 63(1), 99–106.

[ajhb23811-bib-0443] Yoshida, K. , Sakurada, T. , Kaise, N. , Yamamoto, M. , Kaise, K. , Saito, S. , & Yoshinaga, K. (1980). Measurement of triiodothyronine in urine. The Tohoku Journal of Experimental Medicine, 132(4), 389–395. 7256725 10.1620/tjem.132.389

[ajhb23811-bib-0444] Zambruni, M. , Ochoa, T. J. , Somasunderam, A. , Cabada, M. M. , Morales, M. L. , Mitreva, M. , Rosa, B. A. , Acosta, G. J. , Vigo, N. I. , & Riveros, M. (2019). Stunting is preceded by intestinal mucosal damage and microbiome changes and is associated with systemic inflammation in a cohort of Peruvian infants. The American Journal of Tropical Medicine and Hygiene, 101(5), 1009–1017. 31482782 10.4269/ajtmh.18-0975 PMC6838574

[ajhb23811-bib-0445] Zera, A. J. , & Harshman, L. G. (2001). The physiology of life history trade‐offs in animals. Annual Review of Ecology and Systematics, 32, 95–126.

[ajhb23811-bib-0446] Zhang, H. , Yao, Z. , Lin, L. , Sun, X. , Shi, X. , & Zhang, L. (2019). Early life stress predicts cortisol response to psychosocial stress in healthy young adults. PsyCh Journal, 8(3), 353–362. 30932372 10.1002/pchj.278

[ajhb23811-bib-0447] Zhang, L. , Wallace, C. D. , Erickson, J. E. , Nelson, C. M. , Gaudette, S. M. , Pohl, C. S. , Karsen, S. D. , Simler, G. H. , Peng, R. , & Stedman, C. A. (2020). Near infrared readouts offer sensitive and rapid assessments of intestinal permeability and disease severity in inflammatory bowel disease models. Scientific Reports, 10(1), 4696. 32170183 10.1038/s41598-020-61756-y PMC7070059

[ajhb23811-bib-0448] Zhang, S. , Cui, Z. , Zhang, Y. , Wang, B. , Zhu, M. , Lu, J. , & Wang, Z. (2018). Low‐ranking individuals present high and unstable fecal cortisol levels in provisioned free‐ranging adult male rhesus macaques (*Macaca mulatta*) during the birth season in a mountain area of northern China. Primates, 59(6), 517–522. 30298460 10.1007/s10329-018-0692-5

[ajhb23811-bib-0449] Ziegler, T. E. , Washabaugh, K. F. , & Snowdon, C. T. (2004). Responsiveness of expectant male cotton‐top tamarins, Saguinus oedipus, to mate's pregnancy. Hormones and Behavior, 45(2), 84–92. 15019794 10.1016/j.yhbeh.2003.09.003

[ajhb23811-bib-0450] Ziegler, T. E. , & Wittwer, D. J. (2005). Fecal steroid research in the field and laboratory: Improved methods for storage, transport, processing, and analysis. American Journal of Primatology, 67(1), 159–174. 16163716 10.1002/ajp.20175

[ajhb23811-bib-0451] Ziomkiewicz, A. , Sancilio, A. , Galbarczyk, A. , Klimek, M. , Jasienska, G. , & Bribiescas, R. G. (2016). Evidence for the cost of reproduction in humans: High lifetime reproductive effort is associated with greater oxidative stress in post‐menopausal women. PLoS One, 11(1), e0145753. 26761206 10.1371/journal.pone.0145753 PMC4711894

[ajhb23811-bib-0452] Zoch, M. L. , Clemens, T. L. , & Riddle, R. C. (2016). New insights into the biology of osteocalcin. Bone, 82, 42–49. 26055108 10.1016/j.bone.2015.05.046 PMC4670816

